# Supplementary material for: Investigating foods and beverages sold and advertised in deprived urban neighbourhoods in Ghana and Kenya: a cross-sectional study
Source: BMJ Open. 2020 Jun 28;10(6):e035680. doi: 10.1136/bmjopen-2019-035680 (PMC7322322; doi:10.1136/bmjopen-2019-035680)
Supplement: Supplementary data [file bmjopen-2019-035680supp001.pdf]

Supplementary Appendices

For the paper: *Investigating foods and beverages sold and advertised in deprived urban neighbourhoods in Ghana and Kenya: A cross-sectional study.*

Mark A Green PhD[1]*, Rebecca Pradeilles PhD[2], Amos Laar PhD[3], Hibbah Osei-Kwasi PhD[4], Nicolas Bricas MA[5], Nathaniel Coleman MPH[3], Senam Klomegah MPhil[6] , Milka Njeri MSc[7], Akua Tandoh MSc[3], Robert Akparibo PhD[8], Richmond Aryeetey PhD[3], Paula Griffiths PhD[2], Elizabeth Kimani PhD[7], Kobby Mensah PhD[9], Stella Muthuri PhD[10], Francis Zotor PhD[6], Michelle Holdsworth PhD[5,11].

[1] Department of Geography & Planning, University of Liverpool, Liverpool, UK
[2] School of Sport, Exercise and Health Sciences, Loughborough University, Loughborough, UK
[3] Department of Population, Family and Reproductive Health, University of Ghana, Accra, Ghana
[4] Department of Geography, University of Sheffield, Sheffield, UK
[5] UMR Moisa, Centre de Coopération Internationale en Recherche Agronomique pour le Développement (CIRAD) & University of Montpellier, Montpellier, France
[6] Department of Family and Community Health, University of Health and Allied Services, Ho, Ghana
[7] African Population and Health Research Centre, Nairobi, Kenya
[8] School of Health and Related Research (ScHARR), University of Sheffield, Sheffield, UK
[9] Business School, University of Ghana, Accra, Ghana
[10] UK Department of International Development (DFID), Nairobi, Kenya.
[11] French National Research Institute for Sustainable Development (IRD), NUTRIPASS Unit: IRD-Univ Montpellier-SupAgro, Montpellier, France

* Corresponding author: Roxby Building, Department of Geography & Planning, University of Liverpool, Liverpool, L69 7ZT, UK. Telephone: +44 151 794 2854. Email: mark.green@liverpool.ac.uk

Green MA, *et al. BMJ Open* 2020; 10:e035680. doi: 10.1136/bmjopen-2019-035680

Appendix A – Location of study sites

Low income neighbourhoods were randomly selected in each city. In Accra, the selection of a neighbourhood was informed by the Accra Poverty Mapping Exercise (CHF International, 2010; https://www.globalcommunities.org/publications/2010-accra-poverty-map.pdf). Four areas were identified as being poverty endemic. Amongst these, Ga Mashie which is made up of James Town and Ussher Town, was purposively selected. A simple random sampling exercise was then applied and James Town was selected as the neighbourhood of interest. In the city of Ho, the United Nations Human Settlements Programme (UN-HABITAT) urban profiling report informed the selection of the study site. The report highlighted that 36% of the population lived in four poor areas within the city: Bankoe, Hliha, Ahoe and Dome (UNHABITAT 2009; https://uni.unhabitat.org/wp-content/uploads/2014/07/Ghana-Ho-City-Profile.pdf). Amongst these four areas, Dome was then randomly selected. In Nairobi, we used data from the Kenya National Bureau of Statistics (KNBS) to identify the deprivation level of locations (wards), and randomly selected Makadara Constituency. Jericho, Bahati, Maringo, Hamza, Makongeni and Mbotela communities in Makadara were purposively selected as these were areas we could feasibly work in.

Figures A1-A3 visualise the data collected within our study sites. Maps were created using R and the 'ggmap' library (1). Base maps were taken from 'stamen' maps. Map tiles by Stamen Design, under CC BY 3.0. Data by OpenStreetMap, under ODbL.

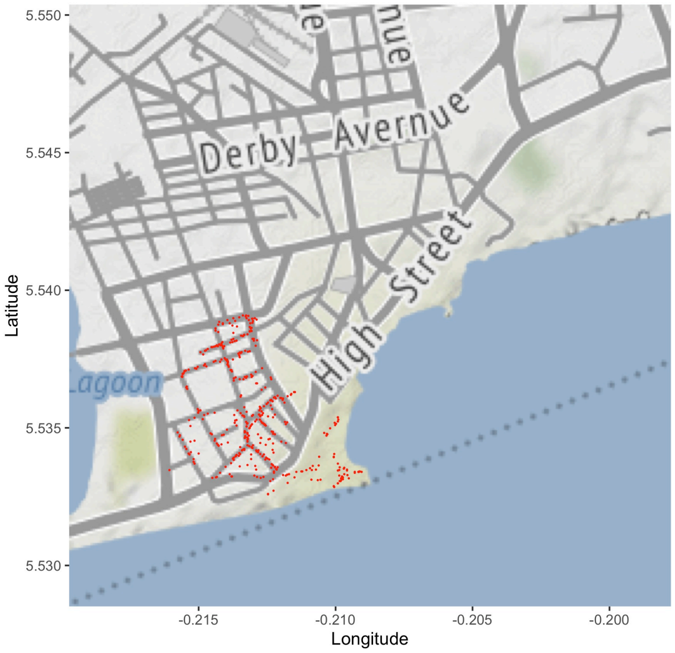

Figure A1: Location of data collection points (red points) within Accra, Ghana.

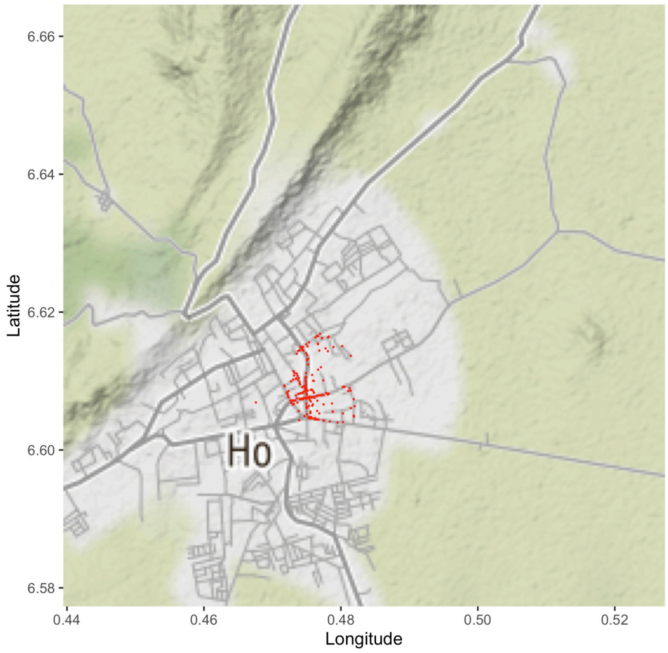

Figure A2: Location of data collection points (red points) within Ho, Ghana.

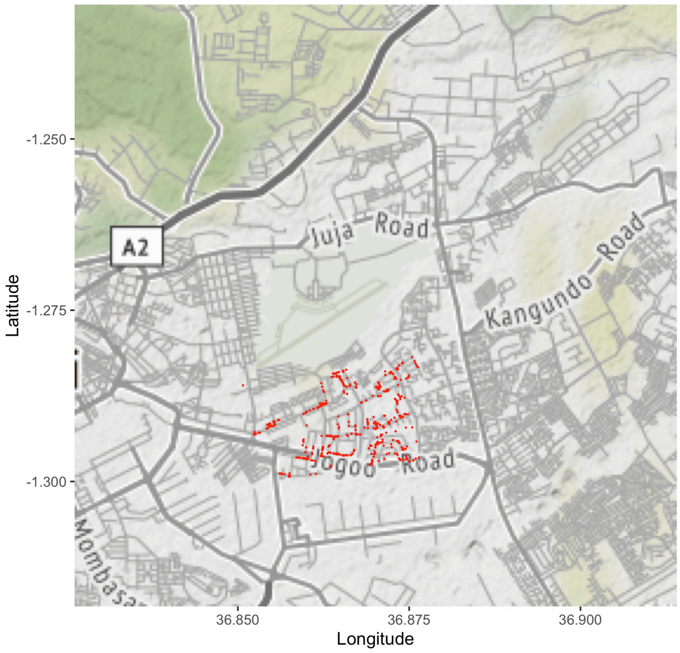

Figure A3: Location of data collection points (red points) within Nairobi, Kenya.

Green MA, *et al. BMJ Open* 2020; 10:e035680. doi: 10.1136/bmjopen-2019-035680

Appendix B – Description of outlet, advert, food and beverage types

Outlet type descriptions

Outlet and advertisement types were defined following a project meeting involving all of the international project partners, researchers and local field workers representing both Ghana and Kenya. The aim was to find consensus over our definitions based on individual expertise and evidence from the wider literature (summarised during discussions), as well as local subject knowledge. Definitions were then validated and refined during the pilot phase of our tool. We opted against using other existing classifications since they were often derived for other settings or countries that were not always relevant to the contexts we were collecting data in. Through designing our own classification, we developed a new system that was relevant to both urban Ghana and Kenya, as well as simple and efficient for data collection.

Table B1: Descriptions of each outlet type.

Outlet type	Description
Bar/pub	A formal vendor primarily selling alcohol
Kiosk	A hut or cubicle selling items
Local vendor	Informal street seller operating on the floor (off a mat) or makeshift counter
Restaurant	A formal organisation where individuals sit and consume foods and beverages on site
Shop	A small and formal building that sells many items
Supermarket	A large organisation selling a diverse set of goods
Vegetable/fruit/food stand/table top	Informal street seller operating from a single table or stand

We did not record mobile local vendors since they did not have a fixed location, though accept that their non-trivial prevalence means our audit has undercounted the number of outlets. We initially included categories for bakeries (n = 1), chop bars and cold stores (Ghana only, n = 11 and 2 respectively), markets (we record each outlet within the market, not the overall market it itself, n = 1) and an 'other' category (n = 36). We did not include these categories in the analyses due to their low prevalence and excluded the data from all analyses.

Table B2: Advertisement descriptions

Outlet type	Description
Onsite	Signs and fittings demonstrating items sold by the outlet
Poster	A small printed paper sign
Painting	The exterior of a building that includes artwork depicting a brand
Billboard	A large outdoor sign

Food and beverage type descriptions

Individual items were classified into broader groups to help improve their interpretation and minimise small number issues. We also grouped items (based on expert opinions and evidence across the literature) into whether we would expect them to increase or decrease in

Green MA, *et al. BMJ Open* 2020; 10:e035680. doi: 10.1136/bmjopen-2019-035680

consumption following the nutrition transition to situate our data within broader nutritional trends in African countries.

Table B3: Food and beverage category descriptions.

Nutrition transition	Category	Examples	Healthy**
Expect to increase	Fats/oils	Cooking oils or fats	No
	Sugar sweetened spreads	Jam, hazelnut spread	No
	Fresh meat/poultry	Raw/uncooked beef, pork, chicken	Yes
	Fresh fish/shellfish	Raw/uncooked	Yes
	Milk	Milk	Yes
	Eggs	Eggs	Yes
	Sugar sweetened beverages	Cola, soda	No
	Alcohol	Beer, vodka, wine	No
	Processed/fried foods	Fried plantain, processed meats, pies	No
	Cakes/sweets	Cake, ice cream, chocolate	No
	Modern mixed dishes	Pizza, lasagne,	No
	Condiments*	Ketchup, shito	No
Expect to decrease	Staples	Grains, cereals, roots, tubers	Yes
	Legumes/pulses	Beans, peas, lentils	Yes
	Nuts/seeds	Cashews, sesame seeds	Yes
	Traditional dishes	Jollof rice, banku, waakye	Yes
	Fruits	Mango, watermelon, oranges	Yes
	Vegetables	Peppers, onions	Yes

* Condiments could include products that are commercially processed from multinational companies, as well as those prepared at home or with local small-scale production.
** The classification of foods and beverages into "healthy" and "unhealthy" was informed by our related work on dietary intake (as part of the broader project), which used a nutrient profiling classification based on recognised methods (2) of foods and beverages consumed in the same cities. We did not classify items during data collection, however applied the classification after to aid the interpretation of our results.

Green MA, *et al. BMJ Open* 2020; 10:e035680. doi: 10.1136/bmjopen-2019-035680

Appendix C – Descriptive statistics on items sold by outlet type

Table C1 presents summary statistics on the number of items sold by outlet type. Outlet types that sold a low number of items were bars/pubs, local vendors and vegetable/fruit/food stand/table top. Restaurants, shops, and kiosks sold a greater number of items. The high proportion of vegetable/fruit/food stand/table top in James Town may help to explain the lower number of items sold on average (and lower variety as well) compared to the other locations. Formal outlets also sold a greater diversity of items than compared to informal outlets.

Table C1: Summary statistics of items sold by outlet type.

Outlet type	Mean items sold	Standard Deviation	Per cent selling 1 item	Per cent selling 2 or fewer items	Per cent selling less than 5 items
Bar/pub	2.63	1.87	17.81	75.34	86.30
Kiosk	4.27	2.84	18.95	34.68	61.69
Local vendor	2.98	2.04	26.85	57.41	77.78
Restaurant	6.60	3.39	0.00	10.00	25.00
Shop	6.78	3.55	10.00	19.50	28.00
Supermarket	10.67	3.84	0.00	0.00	11.11
Vegetable/fruit/food stand/table top	3.05	1.94	22.25	47.52	81.59
Informal	3.46	2.38	15.36	31.41	57.34
Formal	5.88	3.76	3.23	9.39	14.29

There were some noticeable differences in the number of advertisements by outlet type (Table C2). You most likely to encounter an advertisement within a supermarket (with the mean count also being three times higher than any other outlet). Bars/pubs also had high proportion of adverts within them, as did restaurants and shops. There was low prevalence in table tops and local vendors. Formal outlets were more likely to contain adverts.

Table C2: Summary statistics of items advertised by outlet type.

Outlet type	Mean items advertised	Standard Deviation	Per cent containing an advert
Bar/pub	1.01	0.84	69.86
Kiosk	0.41	1.06	24.60
Local vendor	0.44	1.30	16.67
Restaurant	1.40	2.19	55.00
Shop	0.95	1.53	48.50
Supermarket	3.44	3.13	88.89
Vegetable/fruit/food stand/table top	0.15	0.77	7.14
Informal	0.28	0.98	10.27
Formal	1.07	1.57	16.34

Green MA, *et al. BMJ Open* 2020; 10:e035680. doi: 10.1136/bmjopen-2019-035680

Table C3: Percentage of foods and beverages sold between formal and informal outlets.

	Formal	Informal
Fats/oils	46.9	16.0
Sugar sweetened spreads	45.2	12.6
Fresh meat/poultry	16.7	16.4
Fresh fish/shellfish	9.8	12.3
Milk	52.5	20.1
Eggs	49.2	30.2
Sugar sweetened beverages	75.7	22.8
Alcohol	40.0	3.5
Processed/fried foods	43.9	40.5
Cakes/sweets	53.8	22.0
Modern mixed dishes	2.3	5.4
Condiments	39.0	16.3
Staples	52.8	47.2
Legumes/pulses	24.3	14.2
Nuts/seeds	27.9	12.2
Traditional dishes	13.1	27.5
Fruits	3.0	16.7
Vegetables	19.0	31.0

Table C4: Percentage of foods and beverages advertised between formal and informal outlets.

	Formal	Informal
Fats/oils	3.6	5.5
Sugar sweetened spreads	4.1	2.7
Fresh meat/poultry	6.5	13.6
Fresh fish/shellfish	3.0	8.2
Milk	27.2	20.9
Eggs	3.0	6.4
Sugar sweetened beverages	50.3	42.7
Alcohol	42.6	5.5
Processed/fried foods	7.1	12.7
Cakes/sweets	10.1	6.4
Modern mixed dishes	3.6	4.5
Condiments	5.9	18.2
Staples	14.2	15.5
Legumes/pulses	1.8	1.8
Nuts/seeds	1.8	1.8
Traditional dishes	3.0	12.7
Fruits	1.8	0.9
Vegetables	1.8	7.3

Green MA, *et al. BMJ Open* 2020; 10:e035680. doi: 10.1136/bmjopen-2019-035680

We compared the foods and beverages being sold (Table C3) and advertised (Table C4) in formal and informal outlets. The items sold between formal and informal outlets were somewhat different. Most products were more common in formal outlets, reflecting that they sold a greater number of items (see Table C1). Noticeably, unhealthy foods were more common in formal outlets (e.g. fats/oils 46.9% vs 16.0%; sugar sweetened spreads 45.2% vs 12.6%), with similar patterns for drinks as well (i.e. sugar sweetened beverages 75.7% vs 22.8%; alcohol 40.0% vs 3.5%). Not all items were more common in formal outlets; vegetables and fruits were more common in informal outlets. Fresh meat, poultry and fish were similar between outlet type. There was little difference in the foods and beverages being advertised. Only alcohol displayed a large difference, with it being uncommon in informal outlets than compared to formal outlets where it was more prevalent.

We repeated these summary statistics by specific outlet type as well (see Tables C5 to C7). In summary, they present similar patterns to those described above. Foods and beverages sold by local vendors and stands/table tops tended to be dominated by healthier foods and raw ingredients (Table C5). Supermarkets and shops had a greater diversity of items sold. Pubs and bars mostly sold alcohol and sugar sweetened beverages, with a few selling snacks or fresh meat/poultry. The foods and beverages sold by outlets differed to those advertised (Table C6). There was less diversity with lower values reported (bar supermarkets). Many of the foods and beverages advertised represented the most common items sold (i.e. the higher values in Table C5). For advert types, drinks were most commonly advertised. These were particularly common in posters, with onsite and painting having a greater diversity of foods and beverages advertised (reflecting outlet fronts advertising what they sold).

Green MA, *et al. BMJ Open* 2020; 10:e035680. doi: 10.1136/bmjopen-2019-035680

Table C5: Percentage of foods and beverages sold by outlet type.

	Supermarket	Shop	Kiosk	Stand/table top	Local vendor	Restaurant	Bar/pub
Fats/oils	88.9	64.0	30.3	10.2	3.5	25.0	0.0
Sugar sweetened spreads	77.8	62.6	24.3	7.5	3.5	15.0	1.4
Fresh meat/poultry	44.4	6.9	16.7	14.2	22.8	80.0	23.3
Fresh fish/shellfish	22.2	5.9	9.2	12.6	18.4	65.0	4.1
Milk	88.9	72.4	34.3	15.8	2.6	25.0	0.0
Eggs	88.9	63.5	39.0	25.7	25.4	50.0	4.1
Sugar sweetened beverages	88.9	77.8	39.8	15.5	8.8	50.0	75.3
Alcohol	11.1	21.7	4.8	2.4	4.4	25.0	98.6
Processed/fried foods	88.9	51.7	36.7	39.1	53.5	75.0	8.2
Cakes/sweets	0.0	74.9	34.3	19.3	3.5	10.0	1.4
Modern mixed dishes	22.2	1.5	3.2	6.2	7.9	5.0	1.4
Condiments	88.9	53.7	26.7	10.5	12.3	10.0	0.0
Staples	77.8	62.1	51.4	42.1	54.4	95.0	12.3
Legumes/pulses	66.7	29.1	21.5	9.7	13.2	40.0	1.4
Nuts/seeds	77.8	36.9	15.1	12.3	5.3	5.0	2.7
Traditional dishes	22.2	4.4	19.5	31.6	31.6	75.0	19.2
Fruits	22.2	2.5	16.3	17.7	14.0	10.0	0.0
Vegetables	33.3	15.3	33.1	30.0	29.8	55.0	17.8

Green MA, *et al. BMJ Open* 2020; 10:e035680. doi: 10.1136/bmjopen-2019-035680

Table C6: Percentage of foods and beverages advertised by outlet type.

	Supermarket	Shop	Kiosk	Stand/table top	Local vendor	Restaurant	Bar/pub
Fats/oils	0.0	6.1	6.6	3.3	5.3	0.0	0.0
Sugar sweetened spreads	25.0	5.1	4.9	0.0	0.0	0.0	0.0
Fresh meat/poultry	0.0	5.1	6.6	13.3	36.8	45.5	2.0
Fresh fish/shellfish	0.0	3.0	3.3	6.7	26.3	18.2	0.0
Milk	50.0	42.4	32.8	6.7	5.3	0.0	0.0
Eggs	12.5	3.0	6.6	10.0	0.0	9.1	0.0
Sugar sweetened beverages	62.5	55.6	52.5	36.7	21.1	45.5	39.2
Alcohol	12.5	15.2	3.3	6.7	10.5	54.5	98.0
Processed/fried foods	37.5	6.1	4.9	20.0	26.3	27.3	0.0
Cakes/sweets	37.5	14.1	9.8	3.3	0.0	0.0	0.0
Modern mixed dishes	12.5	4.0	3.3	6.7	5.3	9.1	0.0
Condiments	12.5	9.1	6.6	30.0	36.8	0.0	0.0
Staples	50.0	18.2	9.8	16.7	31.6	18.2	0.0
Legumes/pulses	12.5	1.0	1.6	3.3	0.0	9.1	0.0
Nuts/seeds	25.0	1.0	3.3	0.0	0.0	0.0	0.0
Traditional dishes	0.0	1.0	6.6	10.0	36.8	18.2	3.9
Fruits	25.0	1.0	0.0	3.3	0.0	0.0	0.0
Vegetables	12.5	1.0	4.9	13.3	5.3	0.0	2.0

Green MA, *et al. BMJ Open* 2020; 10:e035680. doi: 10.1136/bmjopen-2019-035680

Table C7: Percentage of foods and beverages advertised by advert type.

	Billboard	Poster	Onsite	Painting
Fats/oils	22.2	3.1	3.6	5.8
Sugar sweetened spreads	11.1	3.6	3.6	1.9
Fresh meat/poultry	11.1	5.7	15.5	13.5
Fresh fish/shellfish	0.0	2.6	10.0	7.7
Milk	22.2	23.8	28.2	21.2
Eggs	11.1	4.7	2.7	1.9
Sugar sweetened beverages	44.4	57.5	49.1	28.8
Alcohol	44.4	34.2	23.6	42.3
Processed/fried foods	0.0	6.2	10.9	13.5
Cakes/sweets	22.2	9.8	10.0	9.6
Modern mixed dishes	11.1	5.7	2.7	3.8
Condiments	11.1	6.2	10.9	17.3
Staples	11.1	15.0	19.1	13.5
Legumes/pulses	0.0	2.6	3.6	0.0
Nuts/seeds	11.1	2.6	0.9	0.0
Traditional dishes	0.0	5.7	10.0	9.6
Fruits	11.1	1.0	0.9	3.8
Vegetables	11.1	3.1	2.7	9.6

Green MA, *et al. BMJ Open* 2020; 10:e035680. doi: 10.1136/bmjopen-2019-035680

Appendix D – Evaluation of the number of latent classes for each model

Latent Class Analysis is an exploratory approach used when no theoretical background can determine what the expected latent classes are. Since no previous studies have undertaken a classification of the food environment in Africa, there was no understanding of what latent classes to expect particularly how many classes best describe the data. To select the number of latent classes in our data, we ran multiple models and compared their model fit. We searched for the parsimonious solution. Each additional latent class in our model would likely result in improved model fit, however additional classes introduce added complexity to the model making it harder to interpret.

We ran latent class analyses on our data varying the number of classes between 1 and 10. We did not consider greater than 10 classes since such models would not form much of a data reduction and be difficult to interpret (making any typology less useful). We then compared three model fit statistics (AIC, BIC and G^2) to assess how well they could explain the data. We searched for distinct 'knee points' in graphs of model fit whereby additional classes did not result in significant model improvement (i.e. added complexity in additional classes resulted in minimal gains in model fit). We also compared the model results where it was not clear to aid our interpretation of which model to select.

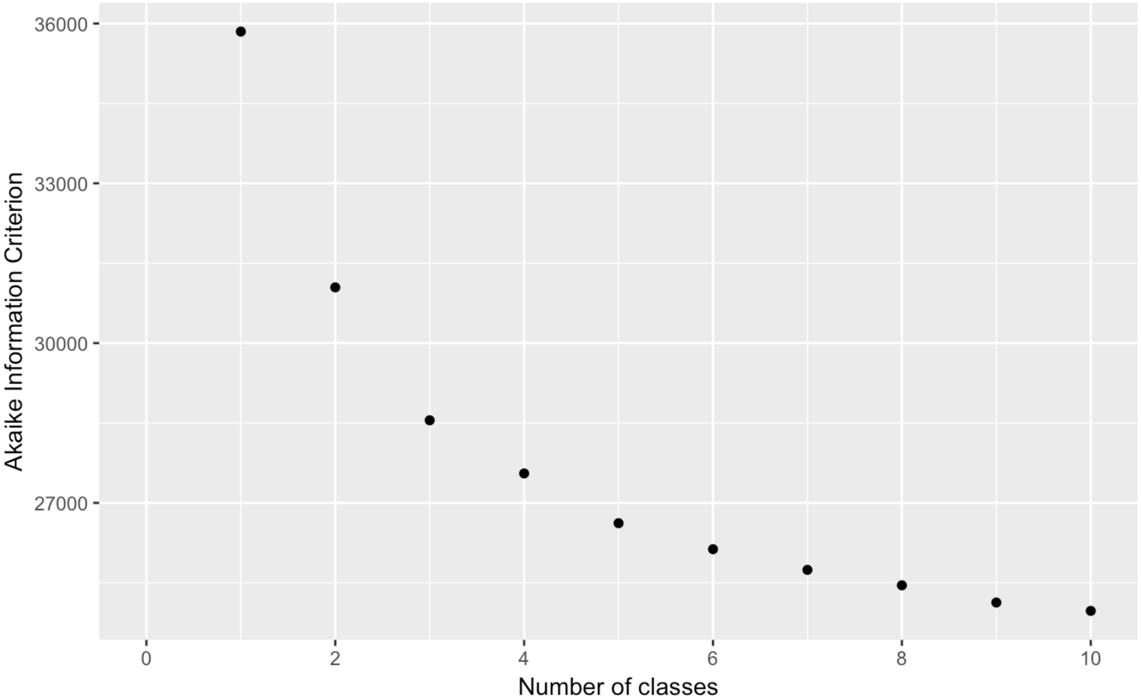

Figure D1: Comparison of Akaike Information Criterion (AIC) for latent class analyses with varying number of classes (1 to 10).

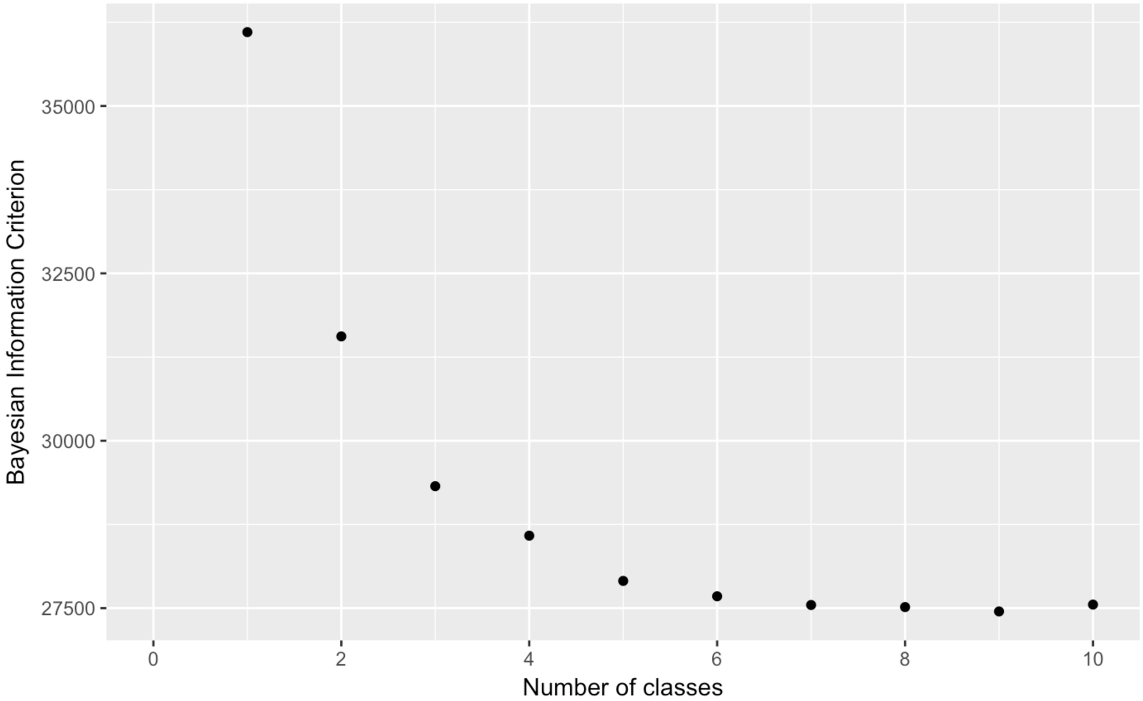

Figure D2: Comparison of Bayesian Information Criterion (BIC) for latent class analyses with varying number of classes (1 to 10).

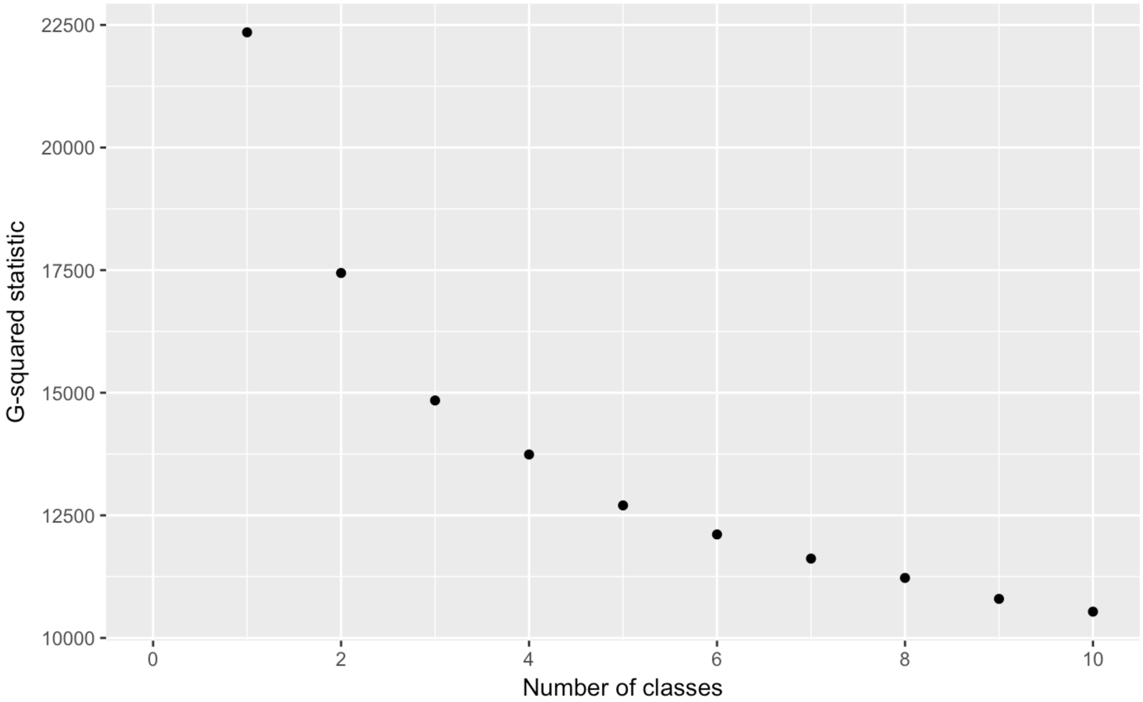

Figure D3: Comparison of G^2 statistic for latent class analyses with varying number of classes (1 to 10).

Green MA, *et al. BMJ Open* 2020; 10:e035680. doi: 10.1136/bmjopen-2019-035680

The AIC and G^2 models produced similar patterns (Figures D1 and D3). For the BIC the pattern was clearer (Figure D2). An increasing number of groups produces better fitting models, but a decreasing rate of improvement. There is no clear knee point though. Following a 5 class solution there is little improvement with subsequent additional class added. Since we are looking for the parsmonious solution, a 5 class solution might work best given that additional classes are not associated with large improvements in model fit. The results for BIC are clearer (Figure D2). We broadly see an improving solution upto 5 classes, whereby model performance is flat onwards. Based on these metrics, a 5 class solution was selected as the final model. Exploring model interpretation of additonal class solutions does not reveal any distinct classes, merely splitting up established classes into less similar classes that differ on small characteristic.

Green MA, *et al. BMJ Open* 2020; 10:e035680. doi: 10.1136/bmjopen-2019-035680

References

1. Kahle D, Wickham H. 2013. ggmap: Spatial Visualization with ggplot2. *The R Journal* 5: 144-161.
2. Drewnowski, A. 2005. Concept of a nutritious food: toward a nutrient density score. *American Journal of Clinical Nutrition* 82: 721–732.
