# Supplementary material for: Investigating foods and beverages sold and advertised in deprived urban neighbourhoods in Ghana and Kenya: a cross-sectional study
Source: BMJ Open. 2020 Jun 28;10(6):e035680. doi: 10.1136/bmjopen-2019-035680 (PMC7322322; doi:10.1136/bmjopen-2019-035680)
Supplement: Author's manuscript [file bmjopen-2019-035680.draft_revisions.pdf]

BMJ Open is committed to open peer review. As part of this commitment we make the peer review history of every article we publish publicly available.

When an article is published we post the peer reviewers' comments and the authors' responses online. We also post the versions of the paper that were used during peer review. These are the versions that the peer review comments apply to.

The versions of the paper that follow are the versions that were submitted during the peer review process. They are not the versions of record or the final published versions. They should not be cited or distributed as the published version of this manuscript.

BMJ Open is an open access journal and the full, final, typeset and author-corrected version of record of the manuscript is available on our site with no access controls, subscription charges or pay-per-view fees (http://bmjopen.bmj.com).

If you have any questions on BMJ Open's open peer review process please email info.bmjopen@bmj.com

BMJ Open

Characterising the food and advertising environments of deprived neighbourhoods in African cities

Journal:	*BMJ Open*
Manuscript ID	bmjopen-2019-035680
Article Type:	Original research
Date Submitted by the Author:	11-Nov-2019
Complete List of Authors:	Green, Mark; University of Liverpool, Geography & Planning<br>Pradeilles, Rebecca; Loughborough University<br>Laar, Amos; School of Public Health, University of Ghana, Department of Population, Family & Reproductive Health<br>Osei-Kwasi, Hibbah; University of Chester<br>Bricas, Nicolas; CIRAD<br>Coleman, Nathaniel ; University of Ghana<br>Klomegah, Senam; University of Health and Allied Sciences<br>Wanjohi, Milka ; African Population and Health Research Center<br>Tandoh, Akua; University of Ghana<br>Akparibo, Robert; The University of Sheffield, Health and Related Research<br>Aryeetey, Richmond; University of Ghana<br>Griffiths, Paula; Loughborough University<br>Kimani-Murage, Elizabeth; African Population and HEalth Research Center, Health Systems and Challenges<br>Mensah, Kobby; University of Ghana<br>Muthuri, Stella ; DFID Kenya<br>Zotor, Francis; University of Health and Allied Sciences,<br>Holdsworth, Michelle; CIRAD
Keywords:	NUTRITION & DIETETICS, PUBLIC HEALTH, STATISTICS & RESEARCH METHODS

SCHOLARONE™
Manuscripts

I, the Submitting Author has the right to grant and does grant on behalf of all authors of the Work (as defined in the below author licence), an exclusive licence and/or a non-exclusive licence for contributions from authors who are: i) UK Crown employees; ii) where BMJ has agreed a CC-BY licence shall apply, and/or iii) in accordance with the terms applicable for US Federal Government officers or employees acting as part of their official duties; on a worldwide, perpetual, irrevocable, royalty-free basis to BMJ Publishing Group Ltd ("BMJ") its licensees and where the relevant Journal is co-owned by BMJ to the co-owners of the Journal, to publish the Work in this journal and any other BMJ products and to exploit all rights, as set out in our licence.

The Submitting Author accepts and understands that any supply made under these terms is made by BMJ to the Submitting Author unless you are acting as an employee on behalf of your employer or a postgraduate student of an affiliated institution which is paying any applicable article publishing charge ("APC") for Open Access articles. Where the Submitting Author wishes to make the Work available on an Open Access basis (and intends to pay the relevant APC), the terms of reuse of such Open Access shall be governed by a Creative Commons licence – details of these licences and which Creative Commons licence will apply to this Work are set out in our licence referred to above.

Other than as permitted in any relevant BMJ Author's Self Archiving Policies, I confirm this Work has not been accepted for publication elsewhere, is not being considered for publication elsewhere and does not duplicate material already published. I confirm all authors consent to publication of this Work and authorise the granting of this licence.

For peer review only

Characterising the food and advertising environments of deprived neighbourhoods in African cities

Mark A Green PhD[1]*, Rebecca Pradeilles PhD[2], Amos Laar PhD[3], Hibbah Osei-Kwasi PhD[4], Nicolas Bricas PhD[5], Nathaniel Coleman MPH[3], Senam Klomegah MPhil[6] , Milka Njeri BSc[7], Akua Tandoh MSc[3], Robert Akparibo PhD[8], Richmond Aryeetey PhD[3], Paula Griffiths PhD[2], Elizabeth Kimani PhD[7], Kobby Mensah PhD[9], Stella Muthuri PhD[10], Francis Zotor PhD[6], Michelle Holdsworth PhD[5].

[1] Department of Geography & Planning, University of Liverpool, Liverpool, UK

[2] School of Sport, Exercise and Health Sciences, Loughborough University, Loughborough, UK

[3] Department of Population, Family and Reproductive Health, University of Ghana, Accra, Ghana

[4] Department of Clinical Sciences and Nutrition, University of Chester, Chester, UK

[5] UMR Moisa, Centre de Coopération Internationale en Recherche Agronomique pour le Développement (CIRAD) & University of Montpellier, Montpellier, France

[6] Department of Family and Community Health, University of Health and Allied Services, Ho, Ghana

[7] African Population and Health Research Centre, Nairobi, Kenya

[8] School of Health and Related Research (ScHARR), University of Sheffield, Sheffield, UK

[9] Business School, University of Ghana, Accra, Ghana

[10] UK Department of International Development (DFID), Nairobi, Kenya

* Corresponding author: Roxby Building, Department of Geography & Planning, University of Liverpool, Liverpool, L69 7ZT, UK. Telephone: +44 151 794 2854. Email: mark.green@liverpool.ac.uk

For peer review only

Abstract

Objectives: The aim of this study was to characterise the local food environment of three deprived African neighbourhoods.

Design: Cross-sectional observational study. We undertook an audit of all food outlets (outlet type and food sold) and food advertisements. Descriptive statistics were used to summarise exposures. Latent class analysis was used to explore the interactions between food advertisements, food outlet types and food type availability.

Setting: Three deprived neighbourhoods in African cities: Jamestown in Accra, Ho Dome in Ho (both Ghana) and Makadara in Nairobi (Kenya).

Main outcome measure**:** Types of foods and beverages sold and/or advertised.

Results: Jamestown (80.5%) and Makadara (70.9%) were dominated by informal vendors. There was a wide diversity of foods, with high availability of healthy (e.g. staples, vegetables) and unhealthy foods (e.g. processed/fried foods, sugar-sweetened beverages). Almost half of all advertisements were for sugar-sweetened beverages (48.3%), with higher exposure to alcohol adverts compared to other items as well (28.5%). We identified five latent classes which demonstrated the clustering of healthier foods in informal outlets, and unhealthy foods in formal outlets.

Conclusion: Our study presents one of the most detailed geospatial exploration of the urban food environment in Africa. The high exposure of sugar-sweetened beverages and alcohol both available and advertised represent changing urban food environments. The concentration of unhealthy foods and beverages in formal outlets and advertisements of unhealthy products may offer important policy opportunities for regulation and action.

Keywords: Built environment; food; adverts; sugar sweetened beverages; alcohol, Africa.

For peer review only

Strengths and Limitations of this Study

- Detailed geospatial data of three deprived neighbourhoods of African cities.
- Inclusion of foods types sold and advertised by outlet which are often not collected.
- Multidimensional analyses consider the interactions between outlet types, foods sold and advertised rather than treating factors independently.
- Limitations include descriptive analyses, lack of representativeness of cities, cross-sectional data, and lack of investigation of which environmental features matter for diet.

Word Count

3867

For peer review only

Introduction

Rising prevalence of overweight and obesity has been seen in most African countries. For instance, the prevalence of obesity in Ghanaian adults (≥20 years) in 2013 was 8.1% and 14% for males and females respectively, and 6.3% and 15.2% respectively among Kenyan adults (Ng *et al.*, 2014). This emerging issue worries policy makers because of the growing prevalence of nutrition-related non-communicable diseases (NR-NCDs) (Mbanya *et al.*, 2014).

Coinciding with these trends in obesity, many African cities have experienced rapid urban growth (Montgomery, 2008; Linard, Tatem and Gilbert, 2013). The rapid growth and upheaval of urban environments resulted in major changes to the built environment, including shops and services (Battersby, Marshak and Mngqibisa, 2016; Ofori-Asenso *et al.*, 2016; Battersby, 2017; Berger and Helvoirt, 2018). This has the potential to shape the local food environment that individuals are exposed to, defined here as the physical services, outlets and structures that promote, advertise and sell food. It includes outlets that sell fresh, pre-packaged and cooked foods such as local vendors, small shops, supermarkets, but also advertisements relating to foods. Local street food vendors are key sources for purchasing foods to cook or eat at home, as well as eat on site (Igumbor *et al.*, 2012). While there are few multinational organisations (e.g. restaurant chains) established in African cities, they are increasingly becoming interested in the African market, where eating out is a sign of affluence (Neupane, Prakash and Doku, 2016). Economic growth has resulted in greater disposable income, which is increasingly being spent on food. There has also been growth in supermarkets providing a greater variety of foods, although such stores are not commonly found within poorer neighbourhoods (Battersby, 2012, 2017; Aryeetey, Oltmans and Owusu, 2016; Berger and Helvoirt, 2018).

Our understanding of the urban African food environment is limited. There are few studies from Africa (mainly concentrated in South Africa (Lachat *et al.*, 2014)), or indeed in low- and middle-income countries in general. This is despite extensive investigation of the nature, role and impact of

For peer review only - http://bmjopen.bmj.com/site/about/guidelines.xhtml

food environments on diet and obesity in high-income countries (Feng *et al.*, 2010; Fleischhacker *et al.*, 2011; Caspi *et al.*, 2012; Cobb *et al.*, 2015). The lack of ecological and environmental research has been identified as a key barrier towards developing successful interventions by policy makers (Holdsworth *et al.*, 2015). This is important since obesity prevalence is higher in urban Africa (including in Ghana and Kenya) (Wu *et al.*, 2015; Neupane, Prakash and Doku, 2016).

Most studies in high-income countries have focused on the location of specific types of outlets (Fleischhacker *et al.*, 2011). Few studies have measured the types of foods sold by outlets that can allow generalisations about whether they are healthy or not (Kelly, Flood and Yeatman, 2011). Food advertisements are rarely incorporated into analyses despite being ever-present features of most environments. These narrow conceptualisations of food environments limit our understanding of the food environment (Rose *et al.*, 2010; Kelly, Flood and Yeatman, 2011; Caspi *et al.*, 2012). We extend previous approaches by utilising a multi-dimensional approach to measuring the food environment, measuring the location of food outlets and adverts, and what food is being sold or advertised. Exploring the interactions between these factors is important to characterise and therefore monitor the food environments populations are exposed to (Mhurchu *et al.*, 2013).

The aim of our study was to characterise the local food environment of deprived neighbourhoods within African cities.

Methodology

Data

Three residential neighbourhoods were selected: Jamestown in Accra (Accra population ~1.5m), Ho Dome in Ho (Ho population ~100,000) both in Ghana, and Makadara in Nairobi, Kenya (Nairobi population ~3.5m). Appendix A presents maps of the three study sites. They were each identified by randomly selecting one deprived neighbourhood or community in each city that was not a slum

For peer review only - http://bmjopen.bmj.com/site/about/guidelines.xhtml

based on the following sources: In Accra, we were informed by the findings of the Accra Poverty Mapping exercise (CHF International, 2010); In Ho, we used data from the United Nations Human Settlements Programme (UN-HABITAT, 2009); Finally, in Nairobi we used ward-level deprivation estimates from the Kenya National Bureau of Statistics.

We conducted a full audit of each neighbourhood. Community audit approaches are common in studies of the food environment and allow the construction of detailed and focused datasets (Caspi *et al.*, 2012). Neighbourhoods were defined through discussion with local community groups, and a 250m buffer was placed around the edge of the community to minimise edge effect issues.

The data collection method was piloted in each setting and further amendments were made to the tool. Data collection occurred between September and December 2017. Trained researchers recorded any food outlet or advert observed within each study site. We recorded the outlet type using a classification we developed during a project partners workshop (a full list of outlets including descriptions can be found in Appendix B). We defined outlet types as 'informal' if their structure of their shop (if any) was moveable, not permanent and/or a small scale operation. Items sold within outlets were selected based on a classification detailed in Appendix B. All food advertisements within an outlet were also recorded. We recorded the type of advert (see Appendix B for description) and the item it was advertising (using same categories as the items sold). We also recorded any standalone adverts including the type of advert and the food advertised. GPS coordinates of outlet and advert locations were recorded using Garmin handheld devices.

Ethical approval for the study was acquired by each institution involved in the data collection process. In Ghana, ethics approval was obtained from the Ghana Health Service Ethics Review Committee (references: GHS-ERC 07/09/16 and GHS-ERC 02/05/17). In Kenya, ethics approval was obtained from the African Medical and Research Foundation (AMREF) (reference: ESRC P365/2017). The University of Sheffield recognised both of these approvals as meeting their ethical standards, as did Loughborough University for the Ghana Health Service. Additional ethical approval was obtained

For peer review only

from the University of Liverpool (references: 1434 and 2288) and Loughborough University (reference: R17 -P142).

Statistical Analysis

Descriptive statistics were used to explore the characteristics of our samples. Latent class analysis was used to identify groups within our data to characterise the food environment. Latent class analysis is useful for identifying subgroups within categorical data that are otherwise unknown (Collins and Lanza, 2010). An exploratory approach was used in the absence of an *a priori* understanding of what groups to expect. We included the outlet type, items being sold, advert type, and items being advertised in the analytical models; although this represents a large number of variables we had no prior justification for dropping variables in this exploratory analysis. The number of latent classes were evaluated through assessing model fit (see Appendix C for details).

Results

Describing the food environments

A total of 413 food outlets or adverts were identified in Jamestown, 208 in Ho Dome, and 499 in Nairobi. We removed 53 observations that were outlets coded as other, resulting in an analytical sample of 1067. Descriptive statistics of the sample are presented in Tables 1 and 2.

Each of the neighbourhoods contained different concentrations of outlet types (Table 1). While the majority of outlets in both Jamestown (80.5%) and Makadara (70.9%) were defined as informal vendors, the nature of outlet types varied by neighbourhood. Jamestown was dominated by vegetable/fruit stand/table tops (63.9%), with low availability of all the other types of outlets apart from kiosks (15.1%). Makadara was more evenly spread between kiosks (40.0%), local vendors (13.43%), vegetable/fruit/food stand/table top (17.5%) and shops (17.9%). Ho Dome was different

For peer review only - http://bmjopen.bmj.com/site/about/guidelines.xhtml

with the majority of outlets defined as formal vendors. There was high availability of shops (43.9%), as well as local vendors (22.8%) and vegetable/fruit/food stand/table top (21.1%).

Table 1: Outlet type and items sold by neighbourhood

Groups	Measure	Jamestown	Ho Dome	Makadara	All
	Outlet type (percentage of all outlets)				
Formal	Bar/pub	8.8	5.0	6.6	7.1
	Restaurant	0.0	2.8	3.3	2.0
	Supermarket	0.5	0.6	1.3	0.9
	Shop	10.1	43.9	17.9	19.6
Informal	Kiosk	15.1	3.9	40.0	24.3
	Local vendor	1.6	22.8	13.4	10.6
	Vegetable/fruit stand/table top	63.9	21.1	17.5	35.6
	All informal* outlets	80.5	47.8	70.9	70.5
	Food sold (percentage of all outlets)				
Foods we expect to increase during the nutrition transition	Fats/oils	13.5	43.3	28.7	25.5
	Sugar sweetened spreads	6.5	49.4	25.6	22.6
	Fresh meat/poultry	16.9	22.8	14.4	16.8
	Fresh fish/shellfish	14.6	17.8	7.2	11.8
	Milk	24.9	43.3	29.3	30.1
	Eggs	27.8	53.9	37.0	36.5
	Sugar-sweetened beverages	35.8	50.0	37.4	39.0
	Alcohol	16.4	26.1	8.3	14.5
	Processed/fried foods	37.4	68.3	36.3	42.4
	Cakes/sweets	23.1	50.0	32.2	31.9
	Modern mixed dishes	8.1	4.4	1.8	4.6
	Condiments	15.1	43.3	22.5	23.4
Foods we expect to decrease	Staples	25.7	55.9	37.0	39.8
	Legumes/pulses	8.6	25.6	21.9	17.5
	Nuts/seeds	9.1	38.3	15.5	17.1
	Traditional dishes	29.9	30.0	16.2	23.8
	Fruits	2.9	6.1	24.1	12.9
	Vegetables	19.5	21.7	37.9	28.1

** Informal/formal outlets were defined by authors and relate to how to whether the establishment is a long-term structure that could be regulated or not, or was small scale in investment/organisation*

Foods sold in outlets demonstrated a good diversity of items. Outlets on average offered a mixture of healthy and unhealthy foods (Table 1). There was high availability of sugar-sweetened beverages (39%), as well as processed/fried foods (42.4%). Most items were more common in Ho Dome. This

For peer review only - http://bmjopen.bmj.com/site/about/guidelines.xhtml

reflected the differences in the prevalence of outlets types, with a higher prevalence of formal vendors that sold a greater number of items. Informal outlets had greater availability of healthier foods and fewer alcohol adverts compared to formal vendors. There was lower prevalence of alcohol sold in Makadara. Fresh meat, poultry and fish were all more common in Jamestown and Ho Dome, with fruits and vegetables more common in Makadara.

Table 2: Food advertisements observed by neighbourhood, type and items advertised

	Jamestown	Ho Dome	Makadara	All
Whether outlets contained an advert (percentage of all records)				
Outlet with advert(s)	25.3	39.1	22.7	26.6
Outlets with no advert	70.2	58.7	76.9	71.1
Standalone advert	4.5	2.2	0.4	2.3
Advert type (percentage of outlets with adverts or standalone adverts)				
Billboard	0.0	7.9	2.8	3.0
Poster	78.3	57.9	50.9	63.6
On site	24.2	36.8	50.0	36.4
Painting	19.2	23.7	10.4	17.2
Foods advertised (percentage of outlets with adverts or standalone adverts)				
Fats/oils	0.8	5.3	7.6	4.3
Sugar sweetened spreads	2.5	1.3	6.6	3.6
Fresh meat/poultry	5.8	15.8	8.5	9.3
Fresh fish/shellfish	2.5	11.8	1.9	4.6
Milk	11.7	30.3	30.2	22.9
Eggs	3.3	0.0	7.6	4.0
Sugar-sweetened beverages	57.5	34.2	48.1	48.3
Alcohol	34.2	31.6	19.8	28.5
Processed/fried foods	6.7	9.2	11.3	8.9
Cakes/sweets	1.7	4.0	18.9	8.3
Modern mixed dishes	4.2	6.6	1.9	4.0
Condiments	8.3	15.8	6.6	9.6
Staples	5.8	23.7	16.0	13.9
Legumes/pulses	0.8	1.3	2.8	1.7
Nuts/seeds	0.0	0.0	4.7	1.7
Traditional dishes	5.8	11.8	3.8	6.6
Fruits	0.0	1.3	2.8	1.3
Vegetables	4.2	2.6	4.7	4.0

A quarter of food outlets contained advertisements and was highest in Ho Dome (Table 2). Few standalone adverts were observed across each location. The low proportion of billboards reflects

For peer review only - http://bmjopen.bmj.com/site/about/guidelines.xhtml

that data collection was conducted in residential neighbourhoods, with fieldwork revealing that billboards were mainly found alongside main roads. Paintings were more commonly observed in Jamestown and Ho Dome than Makadara. Posters were the most common type of advert found (63.6%). 'On Site/Front of Outlet' advertisements were also fairly common especially in Makadara, with large scale paintings less prevalent.

On average, the types of foods advertised were mainly different from those sold by outlets. The most common item advertised was sugar-sweetened beverages (48.3%) and this was consistent across each neighbourhood (including over half of all occasions where adverts were observed in Jamestown). Milk products were also more commonly advertised than most other items. Alcohol was also widely advertised in Jamestown and Ho Dome, but not in Makadara.

For peer review only

A typology of the food environment

We sought to examine the interactions in our measures and classify the food environment. A five-class model was selected as the most appropriate typology (see Appendix C). To help our interpretation of each model, Figure 1 presents radial plots of the conditional response probabilities that describe class characteristics. Where lines reach outwards they represent higher probabilities and therefore a class being more likely to contain that particular variable. We have provided qualitative names for each class to further help their interpretation. Interpretation of the latent classes:

1. *Informal outlets selling raw ingredients*: It was the smallest class ($\gamma = 0.06$). There was an even spread of outlet types observed, although the majority were informal vendors ($\rho = 0.68$). All outlets contained adverts, with higher proportions of posters ($\rho = 0.48$), on site ($\rho = 0.38$) and paintings ($\rho = 0.28$). Foods sold were higher for traditional raw and unprocessed ingredients including staples ($\rho = 0.81$), traditional dishes ($\rho = 0.82$), fresh meat/poultry ($\rho =$

For peer review only - http://bmjopen.bmj.com/site/about/guidelines.xhtml

0.69), and eggs (ρ = 0.50) (although note the high rate of processed/fried foods as well; ρ = 0.72). Advertisements largely followed the foods sold.

2. *Formal outlets selling unhealthy foods with no adverts*: The class was fairly small (γ = 0.13). The majority of outlets were shops (ρ = 0.60), with the remaining proportion mostly made up of kiosks (ρ = 0.30). There was high availability of unhealthy foods sold particularly items typically added to the cooking process such as fats/oils (ρ = 0.83), sugar sweetened spreads (ρ = 0.83), and condiments (ρ = 0.68). Sugar-sweetened beverages (ρ = 0.84) and cakes/sweets (ρ = 0.89) were also common. There was good availability of some healthier foods such as milk (ρ = 0.85), eggs (ρ = 0.76) and staples (ρ = 0.67). There was low availability of fresh meat, poultry, fish, fruit or vegetables. There were no advertisements.
3. *Drinking establishments*: A relatively small class (γ = 0.14). Outlets were mainly formal vendors and the majority contained adverts. The class had the highest prevalence of pubs/bars (ρ = 0.39), as well as standalone adverts (ρ = 0.16). Adverts were mainly posters (ρ = 0.58), although there were above average proportions for the other advert types. The class was characterised by the highest proportion of alcohol and sugar-sweetened beverage sold and advertised. There were few other items sold or advertised.
4. *Small informal sellers*: The largest class (γ = 0.57) was mostly informal outlets (ρ = 0.95) with no adverts. It had the highest prevalence of vegetable/fruit stand/table top (ρ = 0.55). There was low availability of most items. This reflects sellers having a low diversity of items, selling only a few items.
5. *Formal outlets selling unhealthy foods with adverts*: The final class was also fairly small (γ = 0.11). The characteristics of the class largely followed class 2, with the noticeable difference being that all outlets contained adverts. Foods that were advertised matched what was being sold. It had the highest proportion of posters (ρ = 0.66) and on site advertisements (ρ = 0.45).

For peer review only - http://bmjopen.bmj.com/site/about/guidelines.xhtml

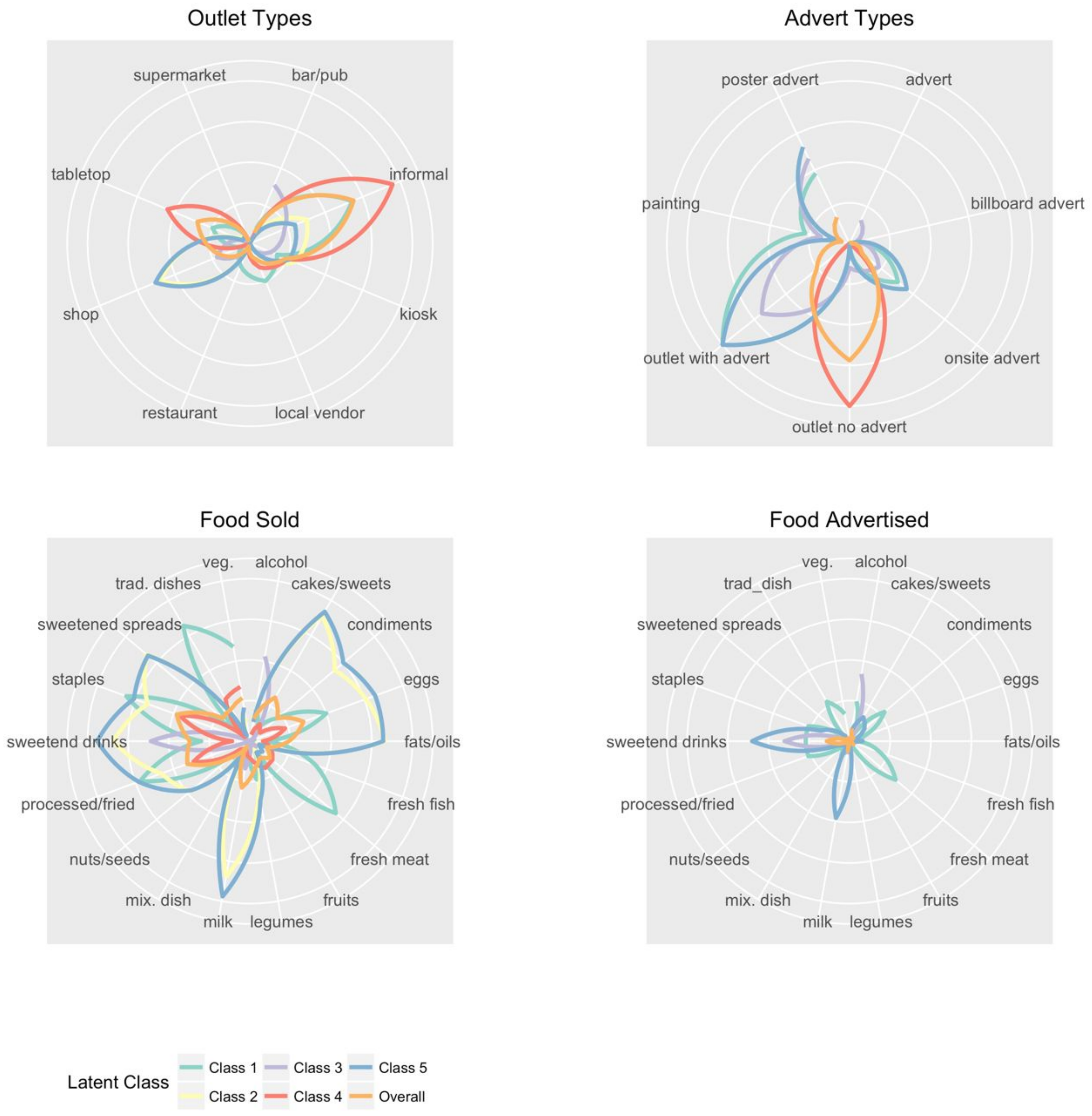

Figure 1: Conditional response probabilities of the latent class model.

We stratified our analyses by neighbourhood, however the results were similar suggesting that our classes were consistent by context (results not reported).

Discussion

Our study presents one of the most detailed investigations of accessibility and exposure to food and advertisements within deprived neighbourhoods of African cities. Our findings have relevance both

For peer review only - http://bmjopen.bmj.com/site/about/guidelines.xhtml

for understanding the food environments within the Ghanaian and Kenyan context, and could be transferable to other African contexts and beyond. We find diverse food environments across each neighbourhood, but also broad similarities. Other than in Ho Dome, outlets were mainly informal vendors (e.g. kiosks or vegetable/fruit stands/table tops). While there was high availability of many healthy foods, there was considerable availability of energy-dense nutrient-poor foods and beverages (e.g. sugar-sweetened beverages, fats/oils, processed/fried foods). Advertisements were most frequent for sodas or sweetened beverages and alcohol, with low advertising for other items (particularly healthy foods).

Our data reveal good physical availability of food across three deprived neighbourhoods. We do not find evidence of food deserts in contrast to social inequalities in high-income countries (Caspi *et al.*, 2012; Cobb *et al.*, 2015; Battersby, 2019). Availability is made possible through the informal sector; local sellers are often mobile allowing them to take opportunities of free space to sell food. The growth of informal outlets reflects the rapid urbanisation and change experienced in cities (Montgomery, 2008; Linard, Tatem and Gilbert, 2013; Battersby, Marshak and Mngqibisa, 2016), and with respect to the food environment our findings suggest that the growth of informal outlets may not necessarily be bad. The greater availability in unhealthy foods sold and advertised within formal outlets may offer potential strategies to policy makers. Formal outlets may be amenable to planning regulations and interventions to promote healthier foods. The same strategy will apply also to advertisements particularly given their focus on sugar-sweetened beverages and alcohol (WHO, 2011). Such recommendations contrast with experiences in many African countries whereby the state often regard formal actors as having positive influences on food systems and therefore are less interested in any form of regulation (Battersby, 2017, 2019).

While we find good physical availability of healthy food, this represents only one aspect of accessibility. There was good availability of energy-dense and nutrient-poor foods as well. Exploring the relationship between availability and consumption of foods represents an important future

For peer review only - http://bmjopen.bmj.com/site/about/guidelines.xhtml

angle for research (Mhurchu *et al.*, 2013; Verstraeten *et al.*, 2016). Acceptability and affordability also represent important domains of accessibility. Our data would suggest that focusing on improving accessibility to healthy foods only through physical availability may not be effective since such food is commonly available. Improving the acceptability or desirability of healthy food can only go so far as well. Affordability is paramount; for example, achieving five servings of fruit and vegetables per person per day would cost 52% of household income in low income countries (Miller *et al.*, 2016). Making healthy food affordable, as well as available, will be a key area for policy (Berger and Helvoirt, 2018; Battersby, 2019).

The higher exposure to sodas or sugar-sweetened beverages in the items sold and advertised was consistent across each location. Such products are often associated with social status, particularly if the product originates from high-income countries (Steyn and Mchiza, 2014). They also reflect considerable efforts by large multinational corporations to increase sales of sugar-sweetened beverages in African nations through investing in infrastructure including adverts (Igumbor *et al.*, 2012). Investment has been pronounced and its effectiveness, when combined alongside television and sponsorship campaigns to improve the acceptability of their products, has altered the food environments of urban areas. Countering these messages through increased education and making healthier alternatives more desirable could be effective, however public health budgets are dwarfed by the marketing budgets of such companies suggesting it may be difficult. Given the association between sugar consumption and NR-NCDs (Popkin, Adair and Ng, 2011), this area represents an urgent policy area.

Alcohol also displayed higher exposure through items sold and advertised. Africa is an emerging market for alcohol producers and companies are using advertisement campaigns to increase the exposure to alcohol impacting on the built environment (WHO, 2011, 2018). Tackling such advertisements represents one potential important area for policy. There was less alcohol available and advertised in Kenya than compared to Ghana. This was despite a similar proportion of bars/pubs

For peer review only - http://bmjopen.bmj.com/site/about/guidelines.xhtml

in each context. While alcohol consumption is far lower in Africa, recent estimates suggest it Is slightly higher in Kenya than compared to Ghana (WHO, 2018). Alcohol licensing is stricter in Kenya with establishments recently required to have a formal liquor sale licence to sell any alcohol (with no sales during the day). It suggests that regulation can be effective in minimising the exposure of alcohol, and might lead to opportunities to tackling other unhealthy items.

A strength of our study is our multi-dimensional approach for measuring the interactions across the food environment. Previous studies tend to focus on measuring single aspects of the food environment and their relationship to health outcomes. While focusing on specific factors is useful for isolating aspects that could be targeted with interventions (Feng *et al.*, 2010), they ignore how features interact together and therefore are reductionist in how they measure the food environment (Rose *et al.*, 2010; Kelly, Flood and Yeatman, 2011; Caspi *et al.*, 2012). For example, the latent classes reveal how the foods sold and advertised differ considerably by outlet type. Our approach offers one way for the effective monitoring of food environments, which is important for targeting interventions (Mhurchu *et al.*, 2013). The unstructured urban growth that most African cities have experienced has made it difficult for governments to regulate environments. Without effective monitoring and targeting systems in place, the continued unregulated growth of urban areas may act as a catalyst for rising prevalence of obesity and NR-NCDs (Ofori-Asenso *et al.*, 2016).

Although our approach provides a nuanced approach for measuring the food environment, it is not all encompassing. While we include a lot of detail in our measures, important aspects of food supply were not collected (e.g. mobile food vendors). We did not consider the role of urban farming of crops and animals (Mackay, 2018). Product placement, shelf space, price and promotional offers are each stimuli influencing food choices within outlets (Kelly, Flood and Yeatman, 2011). Data were static representing a single time point, but African food environments evolve through the day, and are vibrant and busy at night. Food hygiene and sanitation surrounding outlets is an important determinant of food borne diseases and diarrhoea. We also focussed on the local neighbourhood

For peer review only

environments, but this ignores the wider environments that individuals engage in during their days e.g. school environments (Verstraeten *et al.*, 2016). Extending our approach towards developing multi-site and multi-scale measures will help to build more nuanced measures that incorporate a greater range of contexts that may influence dietary behaviours. Our study though provides a useful starting point for developing more nuanced and detailed understanding of the food environment.

There are several limitations to our study. While our study sites provide insightful findings of the local food environment, each location is not representative of its city. The rapid growth of many African cities has been unplanned, unstructured and almost random resulting in diverse and heterogeneous communities (Doan and Oduro, 2012). Extending study sites to a greater range of neighbourhoods will help to evaluate the interpretation our findings. Our data are cross-sectional and could have been improved through longitudinal observations to explore the consistency of our findings. This is a common issue in studies of the food environment (Feng *et al.*, 2010; Fleischhacker *et al.*, 2011; Cobb *et al.*, 2015). Finally, our study is purely descriptive of food environments and future studies should extend our analyses to explore the extent that exposure to food sold and advertised influence dietary behaviours.

Conclusions

Our study presents one of the most detailed explorations of the food environment in African cities. The physical built environment is a largely ignored aspect of food policy focus, however our findings demonstrate the need to consider the geographical context relating to the foods and beverages sold and advertised within neighbourhoods. Healthy foods commonly available within each neighbourhood suggests that focusing on availability alone may not be effective. Regulating formal marketing and advertisements may be more appropriate, and understanding how to effectively intervene to reduce their exposure will be important for effective policy implementation.

For peer review only - http://bmjopen.bmj.com/site/about/guidelines.xhtml

Funding Statement

This work was supported by two funders. The ‘Dietary transitions in Ghana’ project was funded by a grant from the Drivers of Food Choice (DFC) Competitive Grants Programme [grant number OPP1110043] which is funded by the Bill & Melinda Gates Foundation and the Department for International Development (DFID), and managed by the University of South Carolina Arnold School of Public Health, USA. DFC supports new research on understanding food choice among the poor in low and middle-income countries, strengthening country-level leadership in nutrition and fostering a global community of food-choice researchers. The TACLED project was funded by a Global Challenges Research Fund (GCRF) Foundation Award led by the MRC [grant number MR/P025153/1], and supported by AHRC, BBSRC, ESRC and NERC, with the aim of improving the health and prosperity of low and middle-income countries. The funders played no role in the design of the study, data collection, data analysis, interpretation of the data, writing of the report or the decision to submit for publication.

Competing Interests Statement

None declared.

Data Sharing Statement

All analytical code can be viewed at https://github.com/markagreen/Food_environment_ghana_kenya. Data will be archived at the University of Sheffield’s Records Management Service (http://www.shef.ac.uk/cics/records). A data repository (Dataverse Network &/or ORDA) will be used for this purpose. Data will be openly available 12 months after the project completion date (June 2019). Data can be made available securely at an earlier date on reasonable request to the corresponding author.

For peer review only - http://bmjopen.bmj.com/site/about/guidelines.xhtml

Author contributions

MAG, AL, RA, PG, EK, FZ, and MH had the idea for the study. All authors were involved in designing the data collection approach and tools. NC, SK, MJ and AT collected the data under the supervision of AL, EK, SM, and FZ. MAG, RP, HOK, NB and MH undertook the statistical analyses, with all authors scrutinising the results. MAG led the writing of the paper, with all authors contributing to revising and approving the paper. The corresponding author (MAG) had full access to all the data in the study and had final responsibility for the decision to submit for publication.

Patient and Public Involvement

No involvement.

References

Aryeetey, R., Oltmans, S. and Owusu, F. (2016) 'Food retail assessment and family food purchase behavior in ashongman estates, Ghana', *African Journal of Food, Agriculture, Nutrition and Development*, 16(4), pp. 11386–11403.

Battersby, J. (2012) 'Beyond the food desert: finding ways to speak about urban food security in south africa', *Geografiska Annaler: Series B, Human Geography*, 94(2), pp. 141–159.

Battersby, J. (2017) 'Food System Transformation in the Absence of Food System Planning: The Case of Supermarket and Shopping Mall Retail Expansion in Cape Town, South Africa', *Built Environment*, 43(3), pp. 417–430.

Battersby, J. (2019) 'The Food Desert as a Concept and Policy Tool in African Cities: An Opportunity and a Risk', *Sustainability*, 11(2), p. 458. doi: 10.3390/su11020458.

Battersby, J., Marshak, M. and Mngqibisa, N. (2016) *Mapping the informal food economy of Cape*

For peer review only

Town, South Africa. Available at: http://hungrycities.net/wp-content/uploads/2017/01/HCP5-2.pdf.

Berger, M. and Helvoirt, B. Van (2018) 'Ensuring food secure cities – Retail modernization and policy implications in Nairobi, Kenya', *Food Policy*. Elsevier, 79, pp. 12–22. doi: 10.1016/j.foodpol.2018.04.004.

Caspi, C. E., Sorensen, G., Subramanian, S. V and Kawachi, I. (2012) 'The local food environment and diet: A systematic review', *Health & Place*, 18(5), pp. 1172–1187.

CHF International (2010) *Accra Poverty Map: A Guide to urban poverty reduction in Accra*.

Cobb, L. K., Appel, L. J., Franco, M., Jones-Smith, J. C., Nur, A. and Anderson, C. A. M. (2015) 'The Relationship of the Local Food Environment with Obesity: A Systematic Review of Methods, Study Quality, and Results', *Obesity*, 23(7), pp. 1331–1344. doi: 10.1002/oby.21118.

Collins, L. M. and Lanza, S. T. (2010) *Latent Class and Latent Transition Analysis*. Hoboken, New Jersey: Wiley.

Doan, P. and Oduro, C. Y. (2012) 'Patterns of Population Growth in Peri-Urban Accra, Ghana', *International Journal of Urban and Regional Research*, 36(6), pp. 1306–1325.

Feng, J., Glass, T. A., Curriero, F. C., Stewart, W. F. and Schwartz, B. S. (2010) 'The built environment and obesity: A systematic review of the epidemiologic evidence', *Health & Place*, 16(2), pp. 175–190.

Fleischhacker, S. E., Evenson, K. R., Rodriguez, D. A. and Ammerman, A. S. (2011) 'A systematic review of fast food access studies', *Obesity Reviews*, 12, pp. e460–e471.

Holdsworth, M., Kruger, A., Nago, E., Lachat, C., Mamiro, P., Smit, K., Garimoi-Orach, C., Kameli, Y., Roberfroid, D. and Kolsteren, P. (2015) 'African stakeholders' views of research options to improve nutritional status in sub-Saharan Africa', *Health Policy and Planning*, 30, pp. 863–874.

Igumbor, E. U., Sanders, D., Puoane, T. R., Tsolekile, L., Schwarz, C., Purdy, C., Durao, S. and Hawkes, C. (2012) '"'Big Food,'" the Consumer Food Environment, Health, and the Policy Response in South

For peer review only

Africa', *PLoS Medicine*, 9(7), p. e1001253.

Kelly, B., Flood, V. M. and Yeatman, H. (2011) 'Measuring local food environments: An overview of available methods and measures', *Health & Place*. Elsevier, 17(6), pp. 1284–1293.

Lachat, C., Roberfroid, D., Broeck, L. Van Den, Briel, N. Van Den, Nago, E., Kruger, A., Holdsworth, M., Orach, C. G. and Kolsteren, P. (2014) 'A decade of nutrition research in Africa: assessment of the evidence base and academic collaboration', *Public Health Nutrition*, 18(10), pp. 1890–1897.

Linard, C., Tatem, A. J. and Gilbert, M. (2013) 'Modelling spatial patterns of urban growth in Africa', *Applied Geography*, 44, pp. 23–32.

Mackay, H. (2018) 'Land Use Policy Mapping and characterising the urban agricultural landscape of two intermediate-sized Ghanaian cities', *Land Use Policy*. Elsevier, 70, pp. 182–197. doi: 10.1016/j.landusepol.2017.10.031.

Mbanya, J. C., Assah, F. K., Saji, J. and Atanga, E. N. (2014) 'Obesity and Type 2 Diabetes in Sub-Sahara Africa', *Current Diabetes Reports*, 14, p. 501.

Mhurchu, C. N., Vandevijvere, S., Waterlander, W., Thornton, L. E., Kelly, B., Cameron, A. J., Snowdon, W. and Swinburn, B. (2013) 'Monitoring the availability of healthy and unhealthy foods and non-alcoholic beverages in community and consumer retail food environments globally', *Obesity Reviews*, 14(S1), pp. S108–S119.

Miller, V., Yusuf, S., Chow, C. K., Dehghan, M., Corsi, D. J., Lock, K., Popkin, B., Rangarajan, S., Khatib, R., Lear, S. A. and Fraser, S. (2016) 'Availability, affordability, and consumption of fruits and vegetables in 18 countries across income levels : findings from the Prospective Urban Rural Epidemiology (PURE) study', *Lancet Global Health*, 4, pp. e695–e703.

Montgomery, M. R. (2008) 'The Urban Transformation of the Developing World', *Science*, 319(5864), pp. 761–764.

For peer review only

For peer review only - http://bmjopen.bmj.com/site/about/guidelines.xhtml

Neupane, S., Prakash, K. C. and Doku, D. T. (2016) 'Overweight and obesity among women: analysis of demographic and health survey data from 32 Sub-Saharan African Countries', *BMC Public Health*, 16, p. 30.

Ng, M., Fleming, T., Robinson, M., Thomson, B., Graetz, N., Margono, C., Mullany, E. C., Biryukov, S., Alfonso, R., Ali, M. K., Ali, R., Guzman, N. A., Ammar, W., Anwari, P., Banerjee, A., Chowdhury, R., Courville, K. J., Criqui, M. H., Cundiff, D. K., Dabhadkar, K. C., Dandona, L., Davis, A., Hankey, G. J., Harewood, H. C., Havmoeller, R., Hay, S., Hernandez, L., Husseini, A., Idrisov, B. T., Eldin, S., Hassan, A., Kengne, A. P., Khader, Y. S., Khang, Y., Kim, D., Kimokoti, R. W., Logroscino, G., Lotufo, P. A., Lu, Y., Ma, J., Mainoo, N. K., Mensah, G. A., Merriman, T. R., Mokdad, A. H., Nisar, M. I., Ohkubo, T., Oti, S. O., Pedroza, A., Prabhakaran, D., Roy, N., Sampson, U., Stapelberg, N. J. C., Sturua, L., Sykes, B. L., Tobias, M., Tran, B. X., Trasande, L., Toyoshima, H., Vos, T., Wang, C., Wang, X., Weiderpass, E., Werdecker, A., Wright, J. L., Yang, Y. C. and Yatsuya, H. (2014) 'Global, regional, and national prevalence of overweight and obesity in children and adults during 1980 – 2013: a systematic analysis for the Global Burden of Disease Study 2013', *The Lancet*, 384, pp. 766–781. doi: 10.1016/S0140-6736(14)60460-8.

Ofori-Asenso, R., Agyeman, A. A., Laar, A. and Boateng, D. (2016) 'Overweight and obesity epidemic in Ghana — a systematic review and meta-analysis', *BMC Public Health*, 16, p. 1239.

Popkin, B. M., Adair, L. S. and Ng, S. W. (2011) 'Global nutrition transition and the pandemic of obesity in developing countries', *Nutrition Reviews*, 70(1), pp. 3–21.

Rose, D., Bodor, J. N., Hutchinson, P. L. and Swalm, C. M. (2010) 'The Importance of a Multi-Dimensional Approach for Studying the Links between Food Access and Consumption', *The Journal of Nutrition*, 140, pp. 1170–1174.

Steyn, N. P. and Mchiza, Z. J. (2014) 'Obesity and the nutrition transition in Sub-Saharan Africa', *Annals of the New York Academy of Sciences*, 1311, pp. 88–101.

For peer review only - http://bmjopen.bmj.com/site/about/guidelines.xhtml

UN-HABITAT (2009) *Ghana: Ho City Profile*.

Verstraeten, R., Leroy, J. L., Pieniak, Z., Ochoa-Aviles, A., Holdsworth, M., Verbeke, W., Maes, L. and Kolsteren, P. (2016) 'Individual and Environmental Factors Influencing Adolescents' Dietary Behavior in Low- and Middle-Income Settings', *PLoS ONE*, 11(7), p. e0157744.

WHO (2011) *Monitoring Alcohol Marketing in Africa*.

WHO (2018) *Global status report on alcohol and health 2018*.

Wu, F., Guo, Y., Chatterji, S., Zheng, Y., Naidoo, N., Jiang, Y., Biritwum, R., Yawson, A., Minicuci, N., Salinas-Rodriguez, A., Manrique-Espinoza, B., Maximova, T., Peltzer, K., Phaswanamafuya, N., Snodgrass, J. J., Thiele, E., Ng, N. and Kowal, P. (2015) 'Common risk factors for chronic non-communicable diseases among older adults in China, Ghana, Mexico, India, Russia and South Africa: the study on global AGEing and adult health (SAGE) wave 1', *BMC Public Health*, 15, p. 88.

For peer review only

For peer review only - http://bmjopen.bmj.com/site/about/guidelines.xhtml

Supplementary Appendices

For the paper: *Characterising the food and advertising environments of deprived neighbourhoods in African Cities*

Mark A Green PhD[1]*, Rebecca Pradeilles PhD[2], Amos Laar PhD[3], Hibbah Osei-Kwasi PhD[4], Nicolas Bricas PhD[5], Nathaniel Coleman MPH[3], Senam Klomegah MPhil[6], Milka Njeri BSc[7], Akua Tandoh MSc[3], Robert Akparibo PhD[2], Richmond Aryeetey PhD[3], Paula Griffiths PhD[8], Elizabeth Kimani PhD[7], Kobby Mensah PhD[9], Stella Muthuri PhD[10], Francis Zotor PhD[6], Michelle Holdsworth PhD[2].

1 Department of Geography & Planning, University of Liverpool, Liverpool, UK
2 School of Health and Related Research (ScHARR), University of Sheffield, Sheffield, UK
3 Department of Population, Family and Reproductive Health, University of Ghana, Accra, Ghana
4 Department of Clinical Sciences and Nutrition, University of Chester, Chester, UK
5 UMR Moisa, Centre de Coopération Internationale en Recherche Agronomique pour le Développement (CIRAD) & University of Montpellier, Montpellier, France
6 Department of Family and Community Health, University of Health and Allied Services, Ho, Ghana
7 African Population and Health Research Centre, Nairobi, Kenya
8 School of Sport, Exercise and Health Sciences, Loughborough University, Loughborough, UK
9 Business School, University of Ghana, Accra, Ghana
10 UK Department of International Development (DFID), Nairobi, Kenya

For peer review only

Appendix A – Location of study sites

Maps were created using R and the 'ggmap' library (Kahle D, Wickham H. 2013. ggmap: Spatial Visualization with ggplot2. *The R Journal* 5: 144-161).

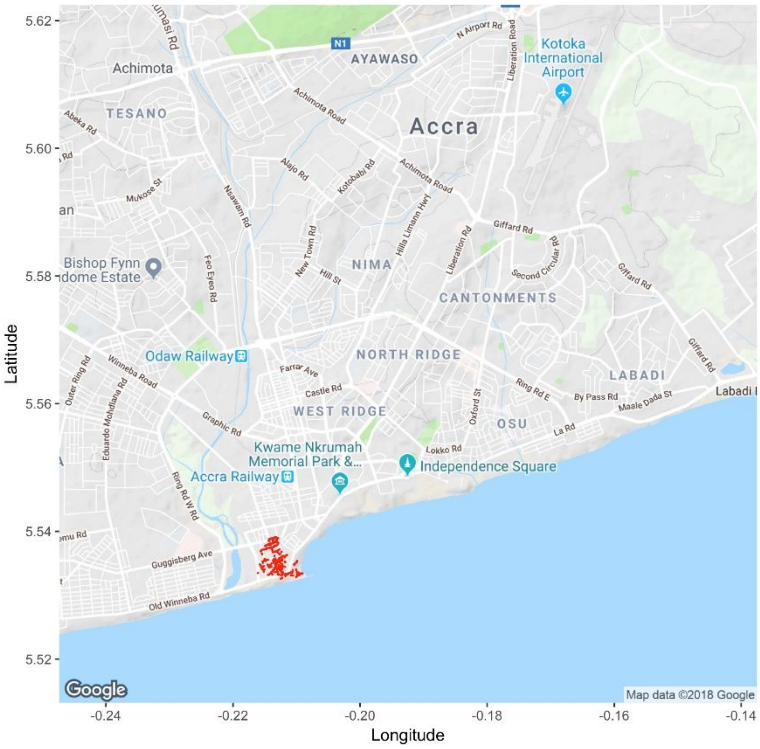

Figure A1: Location of data collection points (red points) within Accra, Ghana.

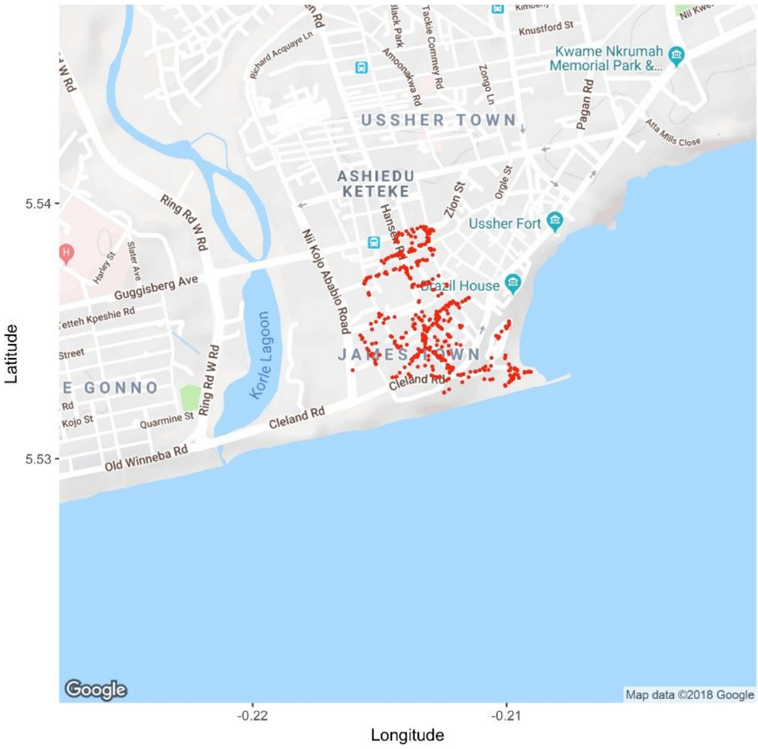

Figure A2: Location of data collection points (red points) within James Town (Accra, Ghana).

2

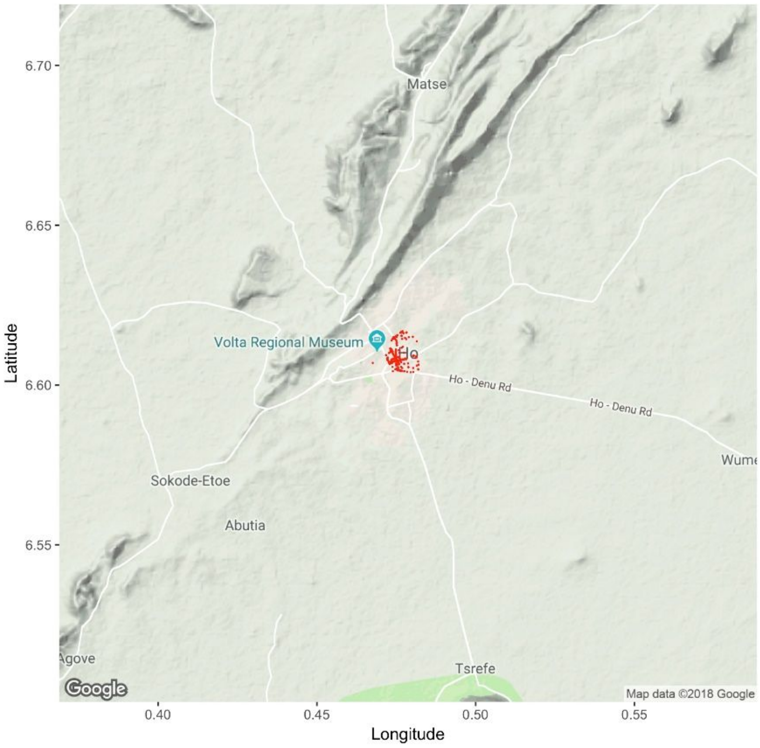

Figure A3: Location of data collection points (red points) within Ho, Ghana.

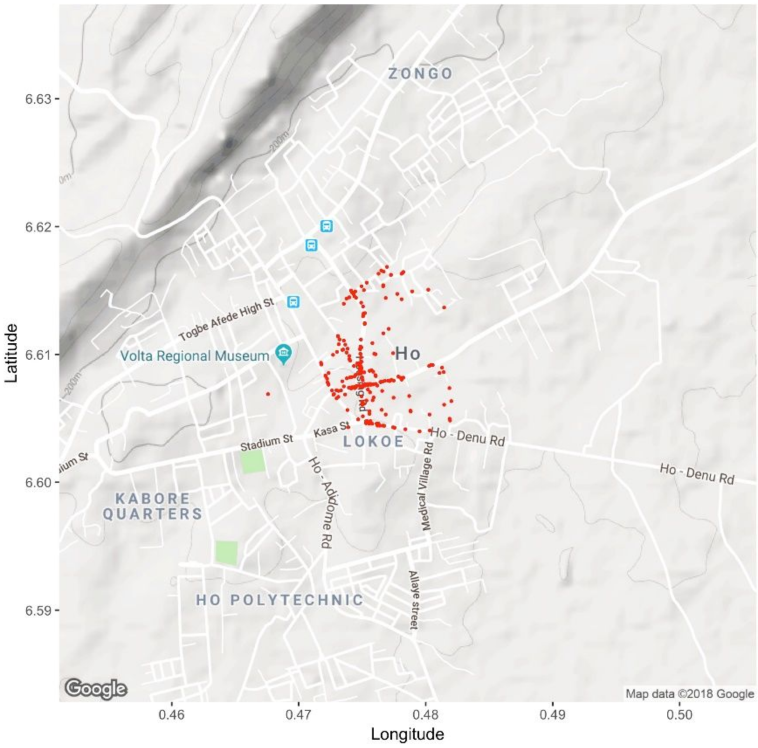

Figure A4: Location of data collection points (red points) within Ho Dome (Ho, Ghana).

For peer review only - http://bmjopen.bmj.com/site/about/guidelines.xhtml

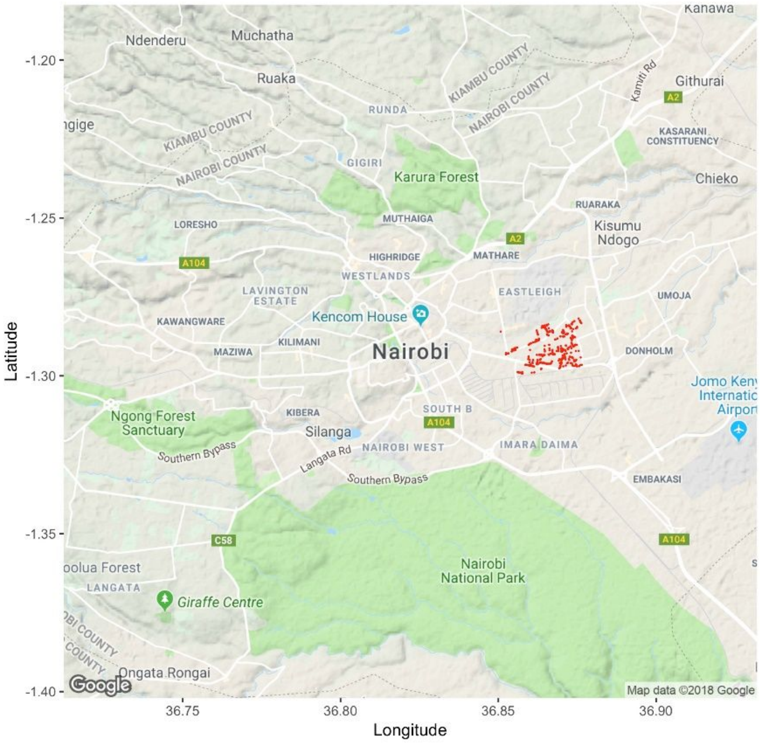

Figure A5: Location of data collection points (red points) within Nairobi, Kenya.

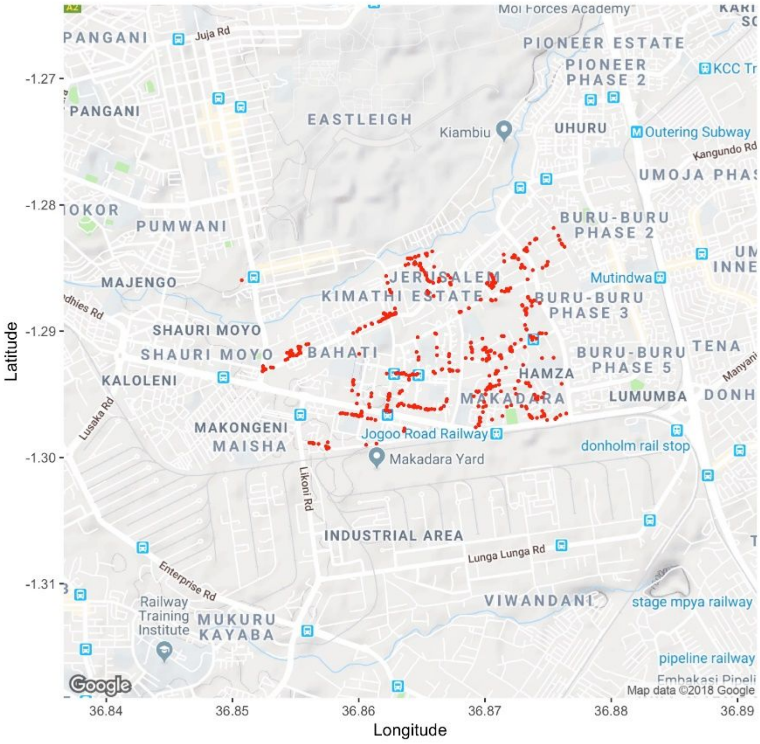

Figure A6: Location of data collection points (red points) within Makadara (Nairobi, Kenya).

For peer review only - http://bmjopen.bmj.com/site/about/guidelines.xhtml

Appendix B – Description of outlet, advert and food/drink types

Outlet type descriptions

Table B1: Descriptions of each outlet type.

Outlet type	Description
Bar/pub	A formal vendor primarily selling alcohol
Kiosk	A hut or cubicle selling items
Local vendor	Informal street seller operating on the floor (off a mat) or makeshift counter
Restaurant	A formal organisation where individuals sit and consume cooked foods on site
Shop	A small and formal building that sells many items
Supermarket	A large organisation selling a diverse set of goods
Vegetable/fruit/food stand/table top	Informal street seller operating from a single table or stand

We did not record mobile local vendors since they did not have a fixed location, though accept that their non-trivial prevalence means our audit has undercounted the number of outlets. We initially included categories for bakeries (n = 1), chop bars and cold stores (Ghana only, n = 11 and 2 respectively), markets (we record each outlet within the market, not the overall market it itself, n = 1) and an 'other' category (n = 36). We did not include these categories in the analyses due to their low prevalence and excluded the data from all analyses.

Table B2: Advertisement descriptions

Outlet type	Description
Onsite	Signs and fittings demonstrating items sold by the outlet
Poster	A small printed paper sign
Painting	The exterior of a building that includes artwork depicting a brand
Billboard	A large outdoor sign

Food type descriptions

Individual items were classified into broader food groups to help improve their interpretation and minimise small number issues. We also grouped foods into whether we would expect them to increase or decrease in consumption following the nutrition transition to situate our data within broader nutritional trends in African countries.

Table B3: Food category descriptions.

Nutrition transition	Food category	Examples
Expect to increase	Fats/oils	Cooking oils or fats
	Sugar sweetened spreads	Jam, hazelnut spread
	Fresh meat/poultry	Raw/uncooked beef, pork, chicken

For peer review only - http://bmjopen.bmj.com/site/about/guidelines.xhtml

	Fresh fish/shellfish	Raw/uncooked
	Milk	Milk
	Eggs	Eggs
	Sugar sweetened beverages	Cola, soda
	Alcohol	Beer, vodka, wine
	Processed/fried foods	Fried plantain, processed meats, pies
	Cakes/sweets	Cake, ice cream, chocolate
	Modern mixed dishes	Pizza, lasagne,
	Condiments	Ketchup, shito
Expect to decrease	Staples	Grains, cereals, roots, tubers
	Legumes/pulses	Beans, peas, lentils
	Nuts/seeds	Cashews, sesame seeds
	Traditional dishes	Jollof rice, banku, waakye
	Fruits	Mango, watermelon, oranges
	Vegetables	Peppers, onions

For peer review only

Appendix C – Evaluation of the number of latent classes for each model

Latent Class Analysis is an exploratory approach if no theoretical background can determine what the expected latent classes are. Since no previous studies have undertaken a classification of the food environment in Africa, there was no understanding of what latent classes to expect particularly how many classes best describe the data. To select the number of latent classes in our data, we ran multiple models and compared their model fit. We searched for the parsimonious solution. Each additional latent class in our model would likely result in improved model fit, however additional classes introduce added complexity to the model making it harder to interpret.

We ran latent class analyses on our data varying the number of classes between 1 and 10. We did not consider greater than 10 classes since such models would not form much of a data reduction and be difficult to interpret (making any typology less useful). We then compared three model fit statistics (AIC, BIC and G^2) to assess how well they could explain the data. We searched for distinct 'knee points' in graphs of model fit whereby additional classes did not result in significant model improvement (i.e. added complexity in additional classes resulted in minimal gains in model fit). We also compared the model results where it was not clear to aid our interpretation of which model to select.

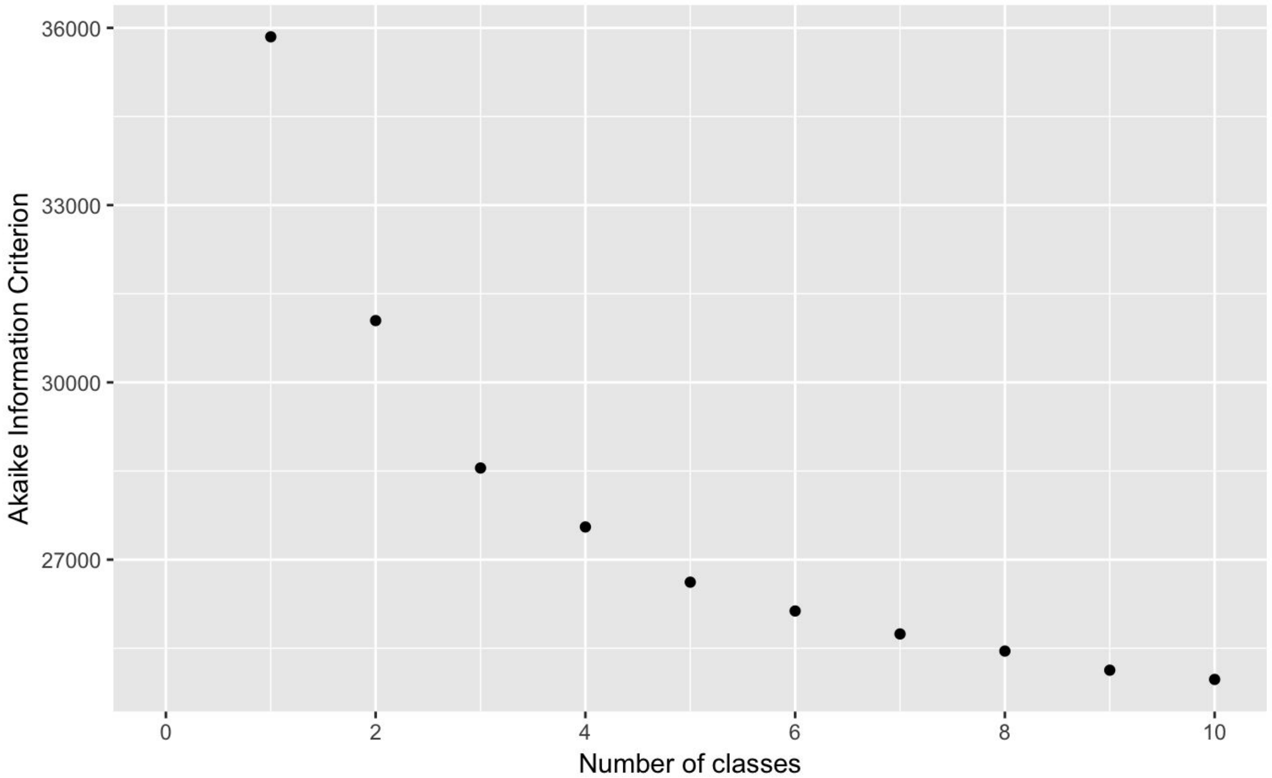

Figure C1: Comparison of Akaike Information Criterion (AIC) for latent class analyses with varying number of classes (1 to 10).

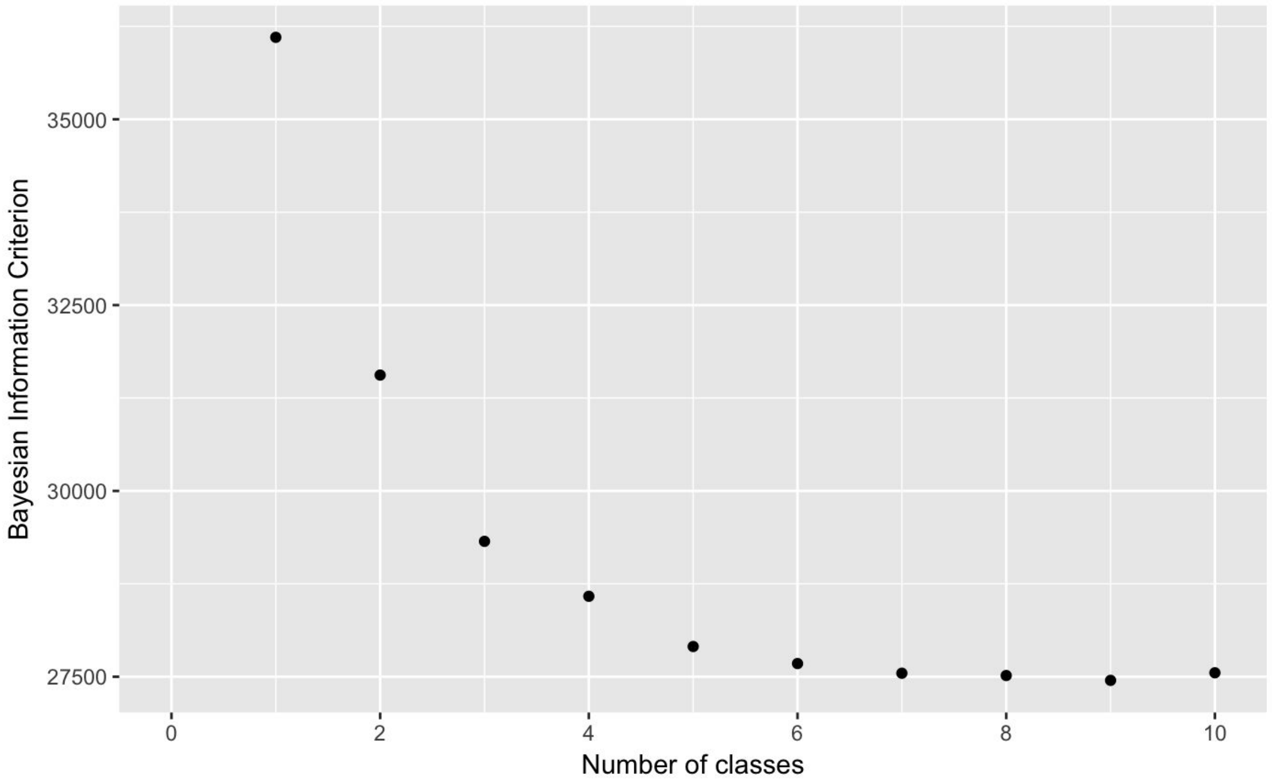

Figure C2: Comparison of Bayesian Information Criterion (BIC) for latent class analyses with varying number of classes (1 to 10).

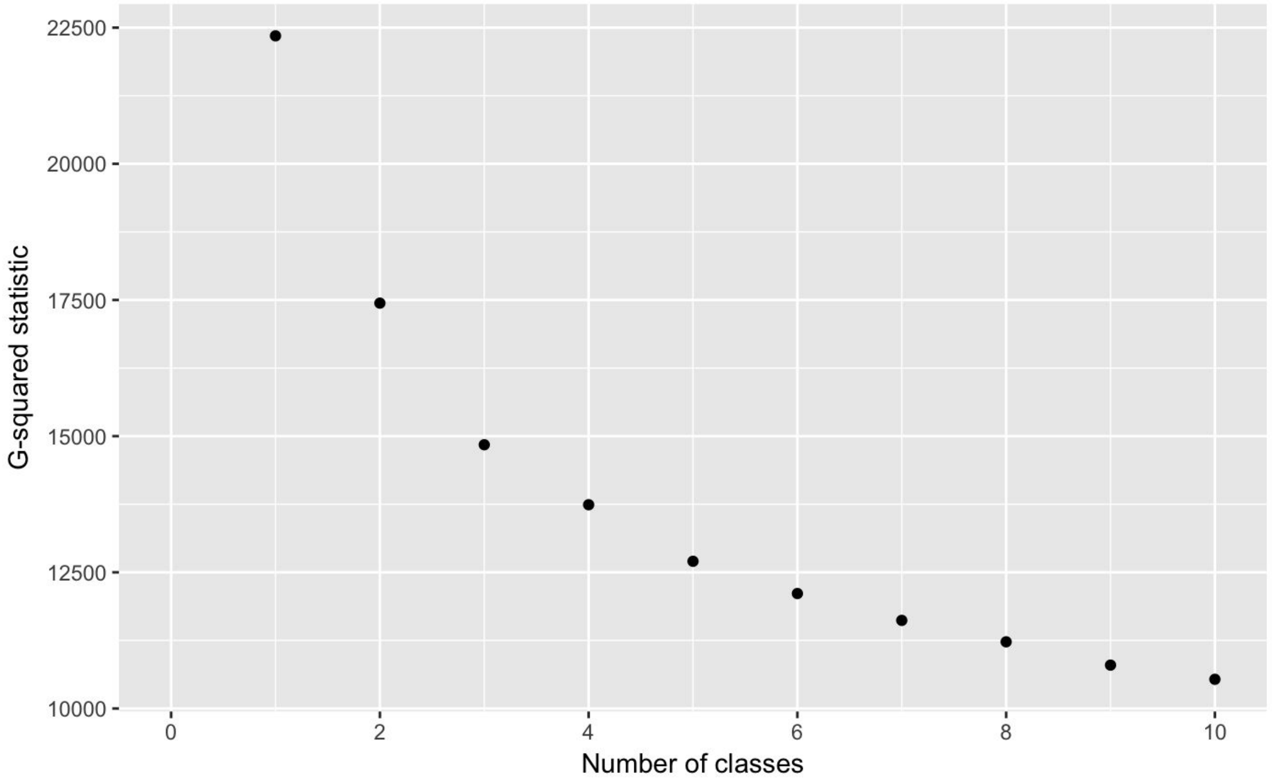

Figure C3: Comparison of G^2 statistic for latent class analyses with varying number of classes (1 to 10).

For peer review only - http://bmjopen.bmj.com/site/about/guidelines.xhtml

The AIC and G^2 models produced similar patterns (Figures C1 and C3). For the BIC the pattern was clearer (Figure C2). An increasing number of groups produces better fitting models, but a decreasing rate of improvement. There is no clear knee point though. Following a 5 class solution there is little improvement with subsequent additional class added. Since we are looking for the parsmonious solution, a 5 class solution might work best given that additional classes are not associated with large improvements in model fit. The results for BIC are clearer (Figure C2). We broadly see an improving solution upto 5 classes, whereby model performance is flat onwards. Based on these metrics, a 5 class solution was selected as the final model. Exploring model interpretation of additonal class solutions does not reveal any distinct classes, merely splitting up established classes into less similar classes that differ on small characteristic.

For peer review only

STROBE 2007 (v4) Statement—Checklist of items that should be included in reports of *cross-sectional studies*

Section/Topic	Item #	Recommendation	Reported on page #
**Title and abstract**	1	(*a*) Indicate the study's design with a commonly used term in the title or the abstract	1
		(*b*) Provide in the abstract an informative and balanced summary of what was done and what was found	2
**Introduction**			
Background/rationale	2	Explain the scientific background and rationale for the investigation being reported	4-5
Objectives	3	State specific objectives, including any prespecified hypotheses	5
**Methods**			
Study design	4	Present key elements of study design early in the paper	5-7
Setting	5	Describe the setting, locations, and relevant dates, including periods of recruitment, exposure, follow-up, and data collection	5-6
Participants	6	(*a*) Give the eligibility criteria, and the sources and methods of selection of participants	N/A
Variables	7	Clearly define all outcomes, exposures, predictors, potential confounders, and effect modifiers. Give diagnostic criteria, if applicable	6 and Appendix B
Data sources/ measurement	8*	For each variable of interest, give sources of data and details of methods of assessment (measurement). Describe comparability of assessment methods if there is more than one group	5-6
Bias	9	Describe any efforts to address potential sources of bias	None
Study size	10	Explain how the study size was arrived at	5-6
Quantitative variables	11	Explain how quantitative variables were handled in the analyses. If applicable, describe which groupings were chosen and why	5-7
Statistical methods	12	(*a*) Describe all statistical methods, including those used to control for confounding	7 and Appendix C
		(*b*) Describe any methods used to examine subgroups and interactions	N/A
		(*c*) Explain how missing data were addressed	N/A
		(*d*) If applicable, describe analytical methods taking account of sampling strategy	7 and Appendix C
		(*e*) Describe any sensitivity analyses	None
**Results**			

For peer review only

Participants	13*	(a) Report numbers of individuals at each stage of study—eg numbers potentially eligible, examined for eligibility, confirmed eligible, included in the study, completing follow-up, and analysed	7
		(b) Give reasons for non-participation at each stage	N/A
		(c) Consider use of a flow diagram	N/A
Descriptive data	14*	(a) Give characteristics of study participants (eg demographic, clinical, social) and information on exposures and potential confounders	7-9
		(b) Indicate number of participants with missing data for each variable of interest	None
Outcome data	15*	Report numbers of outcome events or summary measures	7-9
Main results	16	(*a*) Give unadjusted estimates and, if applicable, confounder-adjusted estimates and their precision (eg, 95% confidence interval). Make clear which confounders were adjusted for and why they were included	7-12
		(*b*) Report category boundaries when continuous variables were categorized	N/A
		(*c*) If relevant, consider translating estimates of relative risk into absolute risk for a meaningful time period	N/A
Other analyses	17	Report other analyses done—eg analyses of subgroups and interactions, and sensitivity analyses	None
**Discussion**			
Key results	18	Summarise key results with reference to study objectives	12-13
Limitations	19	Discuss limitations of the study, taking into account sources of potential bias or imprecision. Discuss both direction and magnitude of any potential bias	15-16
Interpretation	20	Give a cautious overall interpretation of results considering objectives, limitations, multiplicity of analyses, results from similar studies, and other relevant evidence	12-16
Generalisability	21	Discuss the generalisability (external validity) of the study results	16
**Other information**			
Funding	22	Give the source of funding and the role of the funders for the present study and, if applicable, for the original study on which the present article is based	17

*Give information separately for cases and controls in case-control studies and, if applicable, for exposed and unexposed groups in cohort and cross-sectional studies.

Note: An Explanation and Elaboration article discusses each checklist item and gives methodological background and published examples of transparent reporting. The STROBE checklist is best used in conjunction with this article (freely available on the Web sites of PLoS Medicine at http://www.plosmedicine.org/, Annals of Internal Medicine at http://www.annals.org/, and Epidemiology at http://www.epidem.com/). Information on the STROBE Initiative is available at www.strobe-statement.org.

For peer review only

BMJ Open

Investigating foods and beverages sold and advertised in deprived urban neighbourhoods in Ghana and Kenya: A cross-sectional study

Journal:	*BMJ Open*
Manuscript ID	bmjopen-2019-035680.R1
Article Type:	Original research
Date Submitted by the Author:	10-Feb-2020
Complete List of Authors:	Green, Mark; University of Liverpool, Geography & Planning<br>Pradeilles, Rebecca; Loughborough University<br>Laar, Amos; School of Public Health, University of Ghana, Department of Population, Family & Reproductive Health<br>Osei-Kwasi, Hibbah; The University of Sheffield, Geography<br>Bricas, Nicolas; CIRAD<br>Coleman, Nathaniel ; University of Ghana<br>Klomegah, Senam; University of Health and Allied Sciences<br>Wanjohi, Milka ; African Population and Health Research Center<br>Tandoh, Akua; University of Ghana<br>Akparibo, Robert; The University of Sheffield, Health and Related Research<br>Aryeetey, Richmond; University of Ghana<br>Griffiths, Paula; Loughborough University<br>Kimani-Murage, Elizabeth; African Population and HEalth Research Center, Health Systems and Challenges<br>Mensah, Kobby; University of Ghana<br>Muthuri, Stella ; DFID Kenya<br>Zotor, Francis; University of Health and Allied Sciences,<br>Holdsworth, Michelle; French Research Institute for Sustainable Development (IRD)
**Primary Subject Heading**:	Global health
Secondary Subject Heading:	Nutrition and metabolism, Public health, Epidemiology
Keywords:	NUTRITION & DIETETICS, PUBLIC HEALTH, STATISTICS & RESEARCH METHODS

SCHOLARONE™
Manuscripts

I, the Submitting Author has the right to grant and does grant on behalf of all authors of the Work (as defined in the below author licence), an exclusive licence and/or a non-exclusive licence for contributions from authors who are: i) UK Crown employees; ii) where BMJ has agreed a CC-BY licence shall apply, and/or iii) in accordance with the terms applicable for US Federal Government officers or employees acting as part of their official duties; on a worldwide, perpetual, irrevocable, royalty-free basis to BMJ Publishing Group Ltd ("BMJ") its licensees and where the relevant Journal is co-owned by BMJ to the co-owners of the Journal, to publish the Work in this journal and any other BMJ products and to exploit all rights, as set out in our licence.

The Submitting Author accepts and understands that any supply made under these terms is made by BMJ to the Submitting Author unless you are acting as an employee on behalf of your employer or a postgraduate student of an affiliated institution which is paying any applicable article publishing charge ("APC") for Open Access articles. Where the Submitting Author wishes to make the Work available on an Open Access basis (and intends to pay the relevant APC), the terms of reuse of such Open Access shall be governed by a Creative Commons licence – details of these licences and which Creative Commons licence will apply to this Work are set out in our licence referred to above.

Other than as permitted in any relevant BMJ Author's Self Archiving Policies, I confirm this Work has not been accepted for publication elsewhere, is not being considered for publication elsewhere and does not duplicate material already published. I confirm all authors consent to publication of this Work and authorise the granting of this licence.

For peer review only

Investigating foods and beverages sold and advertised in deprived urban neighbourhoods in Ghana and Kenya: A cross-sectional study

Mark A Green PhD[1]*, Rebecca Pradeilles PhD[2], Amos Laar PhD[3], Hibbah Osei-Kwasi PhD[4], Nicolas Bricas MA[5], Nathaniel Coleman MPH[3], Senam Klomegah MPhil[6] , Milka Njeri Wanjohi MSc[7], Akua Tandoh MSc[3], Robert Akparibo PhD[8], Richmond Aryeetey PhD[3], Paula Griffiths PhD[2], Elizabeth Kimani PhD[7], Kobby Mensah PhD[9], Stella Muthuri PhD[10], Francis Zotor PhD[6], Michelle Holdsworth PhD[5,11].

[1] Department of Geography & Planning, University of Liverpool, Liverpool, UK

[2] School of Sport, Exercise and Health Sciences, Loughborough University, Loughborough, UK

[3] Department of Population, Family and Reproductive Health, University of Ghana, Accra, Ghana

[4] Department of Geography, University of Sheffield, Sheffield, UK

[5] UMR Moisa, Centre de Coopération Internationale en Recherche Agronomique pour le Développement (CIRAD) & University of Montpellier, Montpellier, France

[6] Department of Family and Community Health, University of Health and Allied Services, Ho, Ghana

[7] African Population and Health Research Centre, Nairobi, Kenya

[8] School of Health and Related Research (ScHARR), University of Sheffield, Sheffield, UK

[9] Business School, University of Ghana, Accra, Ghana

[10] UK Department of International Development (DFID), Nairobi, Kenya.

[11] French National Research Institute for Sustainable Development (IRD), NUTRIPASS Unit: IRD-Univ Montpellier-SupAgro, Montpellier, France

* Corresponding author: Roxby Building, Department of Geography & Planning, University of Liverpool, Liverpool, L69 7ZT, UK. Telephone: +44 151 794 2854. Email: mark.green@liverpool.ac.uk

For peer review only

Abstract

Objectives: The aim of this study was to characterise the local foods and beverages sold and advertised in three deprived urban African neighbourhoods.

Design: Cross-sectional observational study. We undertook an audit of all food outlets (outlet type and food sold) and food advertisements. Descriptive statistics were used to summarise exposures. Latent class analysis was used to explore the interactions between food advertisements, food outlet types and food type availability.

Setting: Three deprived neighbourhoods in African cities: Jamestown in Accra, Ho Dome in Ho (both Ghana) and Makadara in Nairobi (Kenya).

Main outcome measure**:** Types of foods and beverages sold and/or advertised.

Results: Jamestown (80.5%) and Makadara (70.9%) were dominated by informal vendors. There was a wide diversity of foods, with high availability of healthy (e.g. staples, vegetables) and unhealthy foods (e.g. processed/fried foods, sugar-sweetened beverages). Almost half of all advertisements were for sugar-sweetened beverages (48.3%), with higher exposure to alcohol adverts compared to other items as well (28.5%). We identified five latent classes which demonstrated the clustering of healthier foods in informal outlets, and unhealthy foods in formal outlets.

Conclusion: Our study presents one of the most detailed geospatial exploration of the urban food environment in Africa. The high exposure of sugar-sweetened beverages and alcohol both available and advertised represent changing urban food environments. The concentration of unhealthy foods and beverages in formal outlets and advertisements of unhealthy products may offer important policy opportunities for regulation and action.

Keywords: Food environment; food; adverts; sugar sweetened beverages; alcohol, Africa.

For peer review only - http://bmjopen.bmj.com/site/about/guidelines.xhtml

Strengths and Limitations of this Study

- Detailed geospatial data of three deprived neighbourhoods of African cities.
- Inclusion of foods types sold and advertised by outlet which are often not collected.
- Multidimensional analyses consider the interactions between outlet types, foods sold and advertised rather than treating factors independently.
- Limitations include descriptive analyses, lack of representativeness of cities, cross-sectional data, and lack of investigation of which environmental features matter for diet.

For peer review only

Word Count

3637

For peer review only - http://bmjopen.bmj.com/site/about/guidelines.xhtml

Introduction

Rising prevalence of overweight and obesity has been seen in most African countries. For instance, the prevalence of obesity in Ghanaian adults (≥20 years) in 2013 was 8.1% and 14% for males and females respectively, and 6.3% and 15.2% respectively among Kenyan adults (1). This emerging issue worries policy makers because of the growing prevalence of nutrition-related non-communicable diseases (NR-NCDs).(2–4) The generalised trends in dietary changes, termed the nutrition transition, include increased consumption of fat, particularly vegetable and edible oils; increased added sugar; increased animal-source foods and decreases in cereals and fibre, specifically in coarse grains, staple cereals and pulses.(5,6) The exact nature of changes in dietary behaviours, and the foods that drive the nutrition transition, vary according to region.(7)

Coinciding with these trends in obesity, many African cities have experienced rapid urban growth.(8,9) The rapid growth and upheaval of urban environments resulted in major changes to the built environment, including shops and services.(10–13) This has the potential to shape the local food environment that individuals are exposed to, defined here as the physical services, outlets and structures that promote, advertise and sell food. It includes outlets that sell fresh, pre-packaged and cooked foods such as local vendors, small shops, supermarkets, but also advertisements relating to foods. Local street food vendors are key sources for purchasing foods to cook or eat at home, as well as eat on site.(14) While there are few multinational organisations (e.g. restaurant chains) established in African cities, they are increasingly becoming interested in the African market, where eating out is a sign of affluence.(15) Economic growth has resulted in greater disposable income, which is increasingly being spent on food. There has also been growth in supermarkets providing a greater variety of foods, although such stores are not commonly found within poorer neighbourhoods.(11,12,16,17)

For peer review only - http://bmjopen.bmj.com/site/about/guidelines.xhtml

Our understanding of the urban African food environment is limited. There are few studies from Africa (mainly concentrated in South Africa (18)), although studies are increasingly exploring urban food environments in middle-income countries which have some parallels.(19,20) This is despite extensive investigation of the nature, role and impact of food environments on diet and obesity in high-income countries.(21–24) The lack of ecological and environmental research has been identified as a key barrier towards developing successful interventions by policy makers.(25) This is important since obesity prevalence is higher in urban Africa (including in Ghana and Kenya).(15,26)

Most studies in high-income countries have focused on the location of specific types of outlets.(23) Few studies have measured the types of foods sold by outlets that can allow generalisations about whether they are healthy or not.(19,27) Food advertisements are rarely incorporated into analyses despite being ever-present features of most environments. These narrow conceptualisations of food environments limit our understanding of the food environment.(21,27,28) We extend previous approaches by utilising a multi-dimensional approach to measuring the food environment, measuring the location of food outlets and adverts, and what food is being sold or advertised. Exploring the interactions between these factors is important to characterise and therefore monitor the food environments populations are exposed to.(29)

The aim of our study was to characterise the local foods and beverages sold and advertised in three deprived urban African neighbourhoods.

For peer review only

Methodology

Data

Three residential neighbourhoods were selected: Jamestown in Accra (Accra population ~1.5m), Ho Dome in Ho (Ho population ~100,000) both in Ghana, and Makadara in Nairobi, Kenya (Nairobi population ~3.5m). Appendix A presents details and maps of the three study sites. They were each

For peer review only - http://bmjopen.bmj.com/site/about/guidelines.xhtml

identified by randomly selecting one deprived neighbourhood or community in each city that was not a slum based on the following sources: In Accra, we were informed by the findings of the Accra Poverty Mapping exercise (30); In Ho, we used data from the United Nations Human Settlements Programme (31); Finally, in Nairobi we used ward-level deprivation estimates from the Kenya National Bureau of Statistics.

We conducted a full audit of each neighbourhood. Community audit approaches are common in studies of the food environment and allow the construction of detailed and focused datasets.(21) Neighbourhoods were defined through discussion with local community groups, and a 250m buffer was placed around the edge of the community to minimise edge effect issues.

The data collection method was piloted in each setting through surveying smaller areas close to where data collection would take place. The tool was tested on each outlet types (number of occasions for each depending on prevalence in an area) and further amendments were made to the tool. Data collection occurred between September and December 2017. Trained researchers recorded any food outlet or advert observed within each study site. We recorded the outlet type using a classification we developed during a project partners workshop (a full list of outlets including descriptions can be found in Appendix B). We defined outlet types as 'informal' if their structure of their shop (if any) was moveable, not permanent and/or a small scale operation. Items sold within outlets were recorded based on a pre-defined framework of how they would be expected to change during the nutrition transition (detailed in Appendix B). All food advertisements within an outlet were also recorded. We recorded the type of advert (see Appendix B for description) and the item it was advertising (using same categories as the items sold). We also recorded any standalone adverts including the type of advert and the food advertised. GPS coordinates of outlet and advert locations were recorded using Garmin handheld devices.

Ethical approval for the study was acquired by each institution involved in the data collection process. In Ghana, ethics approval was obtained from the Ghana Health Service Ethics Review

For peer review only

Committee (references: GHS-ERC 07/09/16 and GHS-ERC 02/05/17). In Kenya, ethics approval was obtained from the African Medical and Research Foundation (AMREF) (reference: ESRC P365/2017). The University of Sheffield recognised both of these approvals as meeting their ethical standards, as did Loughborough University for the Ghana Health Service. Additional ethical approval was obtained from the University of Liverpool (references: 1434 and 2288) and Loughborough University (reference: R17 -P142).

Statistical Analysis

Descriptive statistics were used to explore the characteristics of our samples. Latent class analysis was used to identify groups within our data to characterise the food environment. Latent class analysis is useful for identifying subgroups within categorical data that are otherwise unknown.(32) An exploratory approach was used in the absence of an *a priori* understanding of what groups to expect. We included the outlet type, items being sold, advert type, and items being advertised in the analytical models; although this represents a large number of variables we had no prior justification for dropping variables in this exploratory analysis. The number of latent classes were evaluated through assessing model fit. Analyses were completed using R statistical software.

Results

Describing the food environments

A total of 413 food outlets or adverts were identified in Jamestown, 208 in Ho Dome, and 499 in Nairobi. We removed 53 observations that were outlets coded as other, resulting in an analytical sample of 1067. Descriptive statistics of the sample are presented in Tables 1 and 2.

Each of the neighbourhoods contained different concentrations of outlet types (Table 1). While the majority of outlets in both Jamestown (80.5%) and Makadara (70.9%) were defined as informal

For peer review only

vendors, the nature of outlet types varied by neighbourhood. Jamestown was dominated by vegetable/fruit stand/table tops (63.9%), with low availability of all the other types of outlets apart from kiosks (15.1%). Makadara was more evenly spread between kiosks (40.0%), local vendors (13.43%), vegetable/fruit/food stand/table top (17.5%) and shops (17.9%). Ho Dome was different with the majority of outlets defined as formal vendors. There was high availability of shops (43.9%), as well as local vendors (22.8%) and vegetable/fruit/food stand/table top (21.1%).

Table 1: Outlet type and items sold by neighbourhood

Groups	Measure	Jamestown	Ho Dome	Makadara	All
	Outlet type (percentage of all outlets)				
Formal	Bar/pub	8.8	5.0	6.6	7.1
	Restaurant	0.0	2.8	3.3	2.0
	Supermarket	0.5	0.6	1.3	0.9
	Shop	10.1	43.9	17.9	19.6
	All formal outlets	19.4	52.3	29.1	29.6
Informal	Kiosk	15.1	3.9	40.0	24.3
	Local vendor	1.6	22.8	13.4	10.6
	Vegetable/fruit stand/table top	63.9	21.1	17.5	35.6
	All informal* outlets	80.5	47.8	70.9	70.5
	Foods and beverages sold (percentage of all outlets)				
Expected to increase during the nutrition transition	Fats/oils	13.5	43.3	28.7	25.5
	Sugar sweetened spreads	6.5	49.4	25.6	22.6
	Fresh meat/poultry	16.9	22.8	14.4	16.8
	Fresh fish/shellfish	14.6	17.8	7.2	11.8
	Milk	24.9	43.3	29.3	30.1
	Eggs	27.8	53.9	37.0	36.5
	Sugar-sweetened beverages	35.8	50.0	37.4	39.0
	Alcohol	16.4	26.1	8.3	14.5
	Processed/fried foods	37.4	68.3	36.3	42.4
	Cakes/sweets	23.1	50.0	32.2	31.9
	Modern mixed dishes	8.1	4.4	1.8	4.6
	Condiments	15.1	43.3	22.5	23.4
Expected to decrease	Staples	25.7	55.9	37.0	39.8
	Legumes/pulses	8.6	25.6	21.9	17.5
	Nuts/seeds	9.1	38.3	15.5	17.1
	Traditional dishes	29.9	30.0	16.2	23.8
	Fruits	2.9	6.1	24.1	12.9
	Vegetables	19.5	21.7	37.9	28.1

For peer review only - http://bmjopen.bmj.com/site/about/guidelines.xhtml

** Informal/formal outlets were defined by authors and relate to whether the establishment is a long-term structure that could be regulated or not, or was small scale in investment/organisation*

Foods and beverages sold in outlets demonstrated a good diversity of items. Outlets on average offered a mixture of healthy and unhealthy foods (Table 1; see Appendix C for more detail). There was high availability of sugar-sweetened beverages (39%), as well as processed/fried foods (42.4%). Most items were more common in Ho Dome. This reflected the differences in the prevalence of outlets types, with a higher prevalence of formal vendors that sold a greater number of items. Informal outlets had greater availability of healthier foods and fewer alcohol adverts compared to formal vendors. There was lower prevalence of alcohol sold in Makadara. Fresh meat, poultry and fish were all more common in Jamestown and Ho Dome, with fruits and vegetables more common in Makadara.

Table 2: Food advertisements observed by neighbourhood, type and items advertised

	Jamestown	Ho Dome	Makadara	All
Whether outlets contained an advert (percentage of all records)				
Outlet with advert(s)	25.3	39.1	22.7	26.6
Outlets with no advert	70.2	58.7	76.9	71.1
Standalone advert	4.5	2.2	0.4	2.3
Advert type (percentage of outlets with adverts or standalone adverts)				
Billboard	0.0	7.9	2.8	3.0
Poster	78.3	57.9	50.9	63.6
On site	24.2	36.8	50.0	36.4
Painting	19.2	23.7	10.4	17.2
Foods and beverages advertised (percentage of outlets with adverts or standalone adverts)				
Fats/oils	0.8	5.3	7.6	4.3
Sugar sweetened spreads	2.5	1.3	6.6	3.6
Fresh meat/poultry	5.8	15.8	8.5	9.3
Fresh fish/shellfish	2.5	11.8	1.9	4.6
Milk	11.7	30.3	30.2	22.9
Eggs	3.3	0.0	7.6	4.0
Sugar-sweetened beverages	57.5	34.2	48.1	48.3
Alcohol	34.2	31.6	19.8	28.5
Processed/fried foods	6.7	9.2	11.3	8.9
Cakes/sweets	1.7	4.0	18.9	8.3
Modern mixed dishes	4.2	6.6	1.9	4.0
Condiments	8.3	15.8	6.6	9.6

For peer review only - http://bmjopen.bmj.com/site/about/guidelines.xhtml

Staples	5.8	23.7	16.0	13.9
Legumes/pulses	0.8	1.3	2.8	1.7
Nuts/seeds	0.0	0.0	4.7	1.7
Traditional dishes	5.8	11.8	3.8	6.6
Fruits	0.0	1.3	2.8	1.3
Vegetables	4.2	2.6	4.7	4.0

A quarter of food outlets contained advertisements and was highest in Ho Dome (Table 2; see Appendix C for more detail). Few standalone adverts were observed across each location. The low proportion of billboards reflects that data collection was conducted in residential neighbourhoods, with fieldwork revealing that billboards were mainly found alongside main roads. Paintings were more commonly observed in Jamestown and Ho Dome than Makadara. Posters were the most common type of advert found (63.6%). 'On Site/Front of Outlet' advertisements were also fairly common especially in Makadara, with large scale paintings less prevalent.

On average, the types of foods and beverages advertised were mainly different from those sold by outlets. The most common item advertised was sugar-sweetened beverages (48.3%) and this was consistent across each neighbourhood (including over half of all occasions where adverts were observed in Jamestown). Milk products were also more commonly advertised than most other items. Alcohol was also widely advertised in Jamestown and Ho Dome, but not in Makadara.

A typology of the food environment

We sought to examine the interactions in our measures and classify the food environment. A five-class model was selected as the most appropriate typology (see Appendix D). To help our interpretation of each model, Figure 1 presents radial plots of the conditional response probabilities that describe class characteristics. Where lines reach outwards they represent higher probabilities and therefore a class being more likely to contain that particular variable. We have provided qualitative names for each class to further help their interpretation. Interpretation of the latent classes:

For peer review only - http://bmjopen.bmj.com/site/about/guidelines.xhtml

1. *Informal outlets selling raw ingredients*: It was the smallest class (γ = 0.06). There was an even spread of outlet types observed, although the majority were informal vendors (ρ = 0.68). All outlets contained adverts, with higher proportions of posters (ρ = 0.48), on site (ρ = 0.38) and paintings (ρ = 0.28). Foods sold were higher for traditional raw and unprocessed ingredients including staples (ρ = 0.81), traditional dishes (ρ = 0.82), fresh meat/poultry (ρ = 0.69), and eggs (ρ = 0.50) (although note the high rate of processed/fried foods as well; ρ = 0.72). Advertisements largely followed the foods sold.
2. *Formal outlets selling unhealthy foods with no adverts*: The class was fairly small (γ = 0.13). The majority of outlets were shops (ρ = 0.60), with the remaining proportion mostly made up of kiosks (ρ = 0.30). There was high availability of unhealthy foods sold particularly items typically added to the cooking process such as fats/oils (ρ = 0.83), sugar sweetened spreads (ρ = 0.83), and condiments (ρ = 0.68). Sugar-sweetened beverages (ρ = 0.84) and cakes/sweets (ρ = 0.89) were also common. There was good availability of some healthier foods such as milk (ρ = 0.85), eggs (ρ = 0.76) and staples (ρ = 0.67). There was low availability of fresh meat, poultry, fish, fruit or vegetables. There were no advertisements.
3. *Drinking establishments*: A relatively small class (γ = 0.14). Outlets were mainly formal vendors and the majority contained adverts. The class had the highest prevalence of pubs/bars (ρ = 0.39), as well as standalone adverts (ρ = 0.16). Adverts were mainly posters (ρ = 0.58), although there were above average proportions for the other advert types. The class was characterised by the highest proportion of alcohol and sugar-sweetened beverage sold and advertised. There were few other items sold or advertised.
4. *Small informal sellers*: The largest class (γ = 0.57) was mostly informal outlets (ρ = 0.95) with no adverts. It had the highest prevalence of vegetable/fruit stand/table top (ρ = 0.55). There was low availability of most items. This reflects sellers having a low diversity of items, selling only a few items.

For peer review only - http://bmjopen.bmj.com/site/about/guidelines.xhtml

5. *Formal outlets selling unhealthy foods with adverts*: The final class was also fairly small (γ = 0.11). The characteristics of the class largely followed class 2, with the noticeable difference being that all outlets contained adverts. Foods that were advertised matched what was being sold. It had the highest proportion of posters (ρ = 0.66) and on site advertisements (ρ = 0.45).

Figure 1: Conditional response probabilities of the latent class model.

We stratified our analyses by neighbourhood, however the results were similar suggesting that our classes were consistent by context (results not reported).

Discussion

Our study presents one of the most detailed investigations of accessibility and exposure to food and beverages sold and advertised within deprived neighbourhoods of African cities. Our findings have relevance both for understanding the food environments within the Ghanaian and Kenyan context, and could be transferable to other African contexts and beyond. We find diverse food environments across each neighbourhood, but also broad similarities. Other than in Ho Dome, outlets were mainly informal vendors (e.g. kiosks or vegetable/fruit stands/table tops). While there was high availability of many healthy foods, there was considerable availability of unhealthy, so called energy-dense nutrient-poor foods and beverages (e.g. sugar-sweetened beverages, fats/oils, processed/fried foods). Advertisements were most frequent for sodas or sweetened beverages, alcohol and milk, with low advertising for other items (particularly healthy foods). These findings require attention since the availability and promotion of unhealthy energy-dense nutrient poor foods within deprived urban African environments may place considerable burdens on health systems. Poor dietary intake and alcohol abuse are key drivers of NCDs accounting for 73.4% of all deaths globally.(4) With ~80%

For peer review only - http://bmjopen.bmj.com/site/about/guidelines.xhtml

of NCDs occurring in low and middle income countries and an increasing burden in sub-Saharan Africa,(3) the consequences of unhealthy environments are potentially considerable.

Our data reveal good physical availability of food and beverages across three deprived neighbourhoods. We do not find evidence of food deserts in contrast to social inequalities in high-income countries.(21,24,33) Availability is made possible through the informal sector; local sellers are often mobile allowing them to take opportunities of free space to sell food. The growth of informal outlets reflects the rapid urbanisation and change experienced in cities,(8,9,13) and with respect to the food environment our findings suggest that the growth of informal outlets may not necessarily be bad. The greater availability in unhealthy foods sold and advertised within formal outlets may offer potential strategies to policy makers. Formal outlets may be amenable to planning regulations and interventions to promote healthier foods.(34) The same strategy will apply also to advertisements particularly given their focus on sugar-sweetened beverages and alcohol.(35) Such recommendations contrast with experiences in many African countries whereby the state often regard formal actors as having positive influences on food systems and therefore are less interested in any form of regulation.(11,33)

While we find good physical availability of healthy food, this represents only one aspect of accessibility. There was good availability of unhealthy foods that are energy-dense and nutrient poor nutrient as well. Exploring the relationship between availability and consumption of foods represents an important future angle for research.(20,29,36) Acceptability and affordability also represent important domains of accessibility. Our data would suggest that focusing on improving accessibility to healthy foods only through physical availability may not be effective since such food is commonly available. Improving the acceptability or desirability of healthy food can only go so far as well. Affordability is paramount; for example, achieving five servings of fruit and vegetables per person per day would cost 52% of household income in low income countries.(37) Making healthy food affordable, as well as available, will be a key area for policy.(12,33)

For peer review only - http://bmjopen.bmj.com/site/about/guidelines.xhtml

The higher exposure to sodas or sugar-sweetened beverages in the items sold and advertised was consistent across each location. Such products are often associated with social status, particularly if the product originates from high-income countries.(38) They also reflect considerable efforts by large multinational corporations to increase sales of sugar-sweetened beverages in African nations through investing in infrastructure including adverts.(14) Investment has been pronounced and its effectiveness, when combined alongside television and sponsorship campaigns to improve the acceptability of their products, has altered the food environments of urban areas.(39) Countering these messages through increased education and making healthier alternatives more desirable could be effective, however public health budgets are dwarfed by the marketing budgets of such companies suggesting it may be difficult. Given the association between sugar consumption and NR-NCDs,(5,40) this area represents an urgent policy area.

Alcohol also displayed higher exposure through items sold and advertised. Africa is an emerging market for alcohol producers and companies are using advertisement campaigns to increase the exposure to alcohol impacting on the built environment.(35,41) Tackling such advertisements represents one potential important area for policy. There was less alcohol available and advertised in Kenya than compared to Ghana. This was despite a similar proportion of bars/pubs in each context. While alcohol consumption is far lower in Africa than Europe, for example, recent estimates suggest it Is slightly higher in Kenya compared to Ghana.(41) Alcohol licensing laws are stricter in Kenya, with establishments recently required to have a formal liquor sale licence to sell any alcohol (with no sales during the day). It suggests that regulation can be effective in minimising exposure to alcohol, and might lead to opportunities to tackling other unhealthy food items.

A strength of our study is our multi-dimensional approach for measuring the interactions across the food environment. Previous studies tend to focus on measuring single aspects of the food environment and their relationship to health outcomes. While focusing on specific factors is useful for isolating dietary components that could be targeted with interventions,(22) they ignore how

For peer review only - http://bmjopen.bmj.com/site/about/guidelines.xhtml

features interact together and therefore are reductionist in how they measure the food environment.(21,27,28) For example, the latent classes reveal how the foods sold and advertised differ considerably by outlet type. Our approach offers one way for the effective monitoring of food environments, which is important for targeting interventions.(29) The unstructured urban growth that most African cities are experiencing has made it difficult for governments to regulate environments. Without effective monitoring and targeting systems in place, the continued unregulated growth of urban areas may act as a catalyst for rising prevalence of obesity and NR-NCDs.(10)

There are several limitations to our study. Although our approach provides a nuanced way to measure the food environment, it is not all encompassing. While we included a lot of detail in our measures, important aspects of food supply were not collected (e.g. mobile food vendors). We did not consider the role of urban farming of crops and animals.(42) Product placement, shelf space, price and promotional offers are each stimuli influencing food choices within outlets.(27) Data were static representing a single time point, but African food environments evolve through the day, and are vibrant and busy at night. Food hygiene and sanitation surrounding outlets is an important determinant of food borne diseases and diarrhoea. We also focussed on the local neighbourhood environments, but this ignores the wider environments that individuals engage in during their days e.g. school environments.(36) Extending our approach towards developing multi-site and multi-scale measures will help to build more nuanced measures that incorporate a greater range of contexts that may influence dietary behaviours. Our study though provides a useful starting point for developing more nuanced and detailed understanding of the food environment.

A further limitation is that while our study sites provide insightful findings of the local food environment, each location is not representative of its city. The rapid growth of many African cities has been unplanned, unstructured and almost random resulting in diverse and heterogeneous communities.(43) Extending study sites to a greater range of neighbourhoods will help to evaluate

For peer review only

the interpretation our findings. Our data are cross-sectional and could have been improved through longitudinal observations to explore the consistency of our findings. This is a common issue in studies of the food environment.(22–24) Finally, our study is purely descriptive of food environments and future studies should extend our analyses to explore the extent that exposure to food sold and advertised influence dietary behaviours.

Conclusions

Our study presents one of the most detailed explorations of the food environment in African cities. The physical built environment is a largely ignored aspect of food policy focus in the urban African context, however our findings demonstrate the need to consider the geographical context relating to the foods and beverages sold and advertised within neighbourhoods. Healthy foods commonly available within each neighbourhood suggests that focusing on availability alone may not be effective. Regulating formal marketing and advertisements may be more appropriate, and understanding how to effectively intervene to reduce their exposure will be important for effective policy implementation.

Funding Statement

This work was supported by two funders. The 'Dietary transitions in Ghana' project was funded by a grant from the Drivers of Food Choice (DFC) Competitive Grants Programme [grant number OPP1110043] which is funded by the Bill & Melinda Gates Foundation and the Department for International Development (DFID), and managed by the University of South Carolina Arnold School of Public Health, USA. DFC supports new research on understanding food choice among the poor in low and middle-income countries, strengthening country-level leadership in nutrition and fostering a global community of food-choice researchers. The TACLED project was funded by a Global

For peer review only - http://bmjopen.bmj.com/site/about/guidelines.xhtml

Challenges Research Fund (GCRF) Foundation Award led by the MRC [grant number MR/P025153/1], and supported by AHRC, BBSRC, ESRC and NERC, with the aim of improving the health and prosperity of low and middle-income countries. The funders played no role in the design of the study, data collection, data analysis, interpretation of the data, writing of the report or the decision to submit for publication.

Competing Interests Statement

None declared.

Patient and Public Involvement

No involvement in the specific study.

Data Sharing Statement

All analytical code can be viewed at https://github.com/markagreen/Food_environment_ghana_kenya. Data will be archived at the University of Sheffield's Records Management Service (http://www.shef.ac.uk/cics/records). A data repository (Dataverse Network &/or ORDA) will be used for this purpose. Data will be openly available 12 months after the project completion date (June 2019). Data can be made available securely at an earlier date on reasonable request to the corresponding author.

For peer review only

Author contributions

MAG, AL, RA, PG, EK, KM, FZ, and MH had the idea for the study. All authors were involved in designing the data collection approach and tools. NC, SK, MNW and AT collected the data under the supervision of AL, EK, SM, and FZ. MAG, RP, HOK, NB and MH undertook the statistical analyses, with all authors scrutinising the results. MAG led the writing of the paper, with all authors contributing to revising and approving the paper. The corresponding author (MAG) had full access to all the data in the study and had final responsibility for the decision to submit for publication.

Patient and Public Involvement

No involvement.

References

1. Ng M, Fleming T, Robinson M, Thomson B, Graetz N, Margono C, et al. Global, regional, and national prevalence of overweight and obesity in children and adults during 1980 – 2013: a systematic analysis for the Global Burden of Disease Study 2013. Lancet. 2014;384:766–81.

2. Mbanya JC, Assah FK, Saji J, Atanga EN. Obesity and Type 2 Diabetes in Sub-Sahara Africa. Curr Diab Rep. 2014;14:501.

3. Gouda H, Charlson F, Sorsdahl K, Ahmadzada S, Ferrari A, Erskine H, et al. Burden of non-communicable diseases in sub-Saharan Africa, 1990–2017: results from the Global Burden of Disease Study 2017. Lancet Glob Heal. 2019;7(10):E1375–87.

4. GBD 2017 Causes of Death Collaborators. Global, regional, and national age-sex-specific mortality for 282 causes of death in 195 countries and territories, 1980–2017: a systematic analysis for the Global Burden of Disease Study 2017. Lancet. 2018;392(10159):1736–88.

For peer review only

5. Popkin BM, Adair LS, Ng SW. Global nutrition transition and the pandemic of obesity in developing countries. Nutr Rev. 2011;70(1):3–21.

6. Vorster H, Kruger A, Margetts B. The nutrition transition in Africa: Can it be steered into a more positive direction? Nutrients. 2011;3:429–41.

7. Rousham E, Pradeilles R, Akparibo R, Aryeetey R, Bash K, Booth A, et al. Dietary behaviours in the context of nutrition transition: a systematic review and meta-analysis in two African countries. Public Health Nutr.

8. Montgomery MR. The Urban Transformation of the Developing World. Science (80-). 2008;319(5864):761–4.

9. Linard C, Tatem AJ, Gilbert M. Modelling spatial patterns of urban growth in Africa. Appl Geogr. 2013;44:23–32.

10. Ofori-Asenso R, Agyeman AA, Laar A, Boateng D. Overweight and obesity epidemic in Ghana — a systematic review and meta-analysis. BMC Public Health. 2016;16:1239.

11. Battersby J. Food System Transformation in the Absence of Food System Planning: The Case of Supermarket and Shopping Mall Retail Expansion in Cape Town, South Africa. Built Environ. 2017;43(3):417–30.

12. Berger M, Helvoirt B Van. Ensuring food secure cities – Retail modernization and policy implications in Nairobi, Kenya. Food Policy. 2018;79:12–22.

13. Battersby J, Marshak M, Mngqibisa N. Mapping the informal food economy of Cape Town, South Africa. 2016.

14. Igumbor EU, Sanders D, Puoane TR, Tsolekile L, Schwarz C, Purdy C, et al. '"Big Food,"' the Consumer Food Environment, Health, and the Policy Response in South Africa. PLoS Med. 2012;9(7):e1001253.

For peer review only

For peer review only - http://bmjopen.bmj.com/site/about/guidelines.xhtml

15. Neupane S, Prakash KC, Doku DT. Overweight and obesity among women: analysis of demographic and health survey data from 32 Sub-Saharan African Countries. BMC Public Health. 2016;16:30.

16. Aryeetey R, Oltmans S, Owusu F. Food retail assessment and family food purchase behavior in ashongman estates, Ghana. African J Food, Agric Nutr Dev. 2016;16(4):11386–403.

17. Battersby J. Beyond the food desert: finding ways to speak about urban food security in south africa. Geogr Ann Ser B, Hum Geogr. 2012;94(2):141–59.

18. Lachat C, Roberfroid D, Broeck L Van Den, Briel N Van Den, Nago E, Kruger A, et al. A decade of nutrition research in Africa: assessment of the evidence base and academic collaboration. Public Health Nutr. 2014;18(10):1890–7.

19. Duran A, Diez Roux A, Latorre Mdo R, Jaime P. Neighborhood socioeconomic characteristics and differences in the availability of healthy food stores and restaurants in Sao Paulo, Brazil. Health Place. 2013;23:39–47.

20. Leite F, de Carvalho Cremm E, de Abreu D, Oliveira M, Budd N, Martins P. Association of neighbourhood food availability with the consumption of processed and ultra-processed food products by children in a city of Brazil: a multilevel analysis. Public Health Nutr. 2018;21(1):189–200.

21. Caspi CE, Sorensen G, Subramanian S V, Kawachi I. The local food environment and diet: A systematic review. Health Place. 2012;18(5):1172–87.

22. Feng J, Glass TA, Curriero FC, Stewart WF, Schwartz BS. The built environment and obesity: A systematic review of the epidemiologic evidence. Health Place. 2010;16(2):175–90.

23. Fleischhacker SE, Evenson KR, Rodriguez DA, Ammerman AS. A systematic review of fast food access studies. Obes Rev. 2011;12:e460–71.

For peer review only

For peer review only - http://bmjopen.bmj.com/site/about/guidelines.xhtml

24. Cobb LK, Appel LJ, Franco M, Jones-Smith JC, Nur A, Anderson CAM. The Relationship of the Local Food Environment with Obesity: A Systematic Review of Methods, Study Quality, and Results. Obesity. 2015;23(7):1331–44.

25. Holdsworth M, Kruger A, Nago E, Lachat C, Mamiro P, Smit K, et al. African stakeholders' views of research options to improve nutritional status in sub-Saharan Africa. Health Policy Plan. 2015;30:863–74.

26. Wu F, Guo Y, Chatterji S, Zheng Y, Naidoo N, Jiang Y, et al. Common risk factors for chronic non-communicable diseases among older adults in China, Ghana, Mexico, India, Russia and South Africa: the study on global AGEing and adult health (SAGE) wave 1. BMC Public Health. 2015;15:88.

27. Kelly B, Flood VM, Yeatman H. Measuring local food environments: An overview of available methods and measures. Health Place. 2011;17(6):1284–93.

28. Rose D, Bodor JN, Hutchinson PL, Swalm CM. The Importance of a Multi-Dimensional Approach for Studying the Links between Food Access and Consumption. J Nutr. 2010;140:1170–4.

29. Mhurchu CN, Vandevijvere S, Waterlander W, Thornton LE, Kelly B, Cameron AJ, et al. Monitoring the availability of healthy and unhealthy foods and non-alcoholic beverages in community and consumer retail food environments globally. Obes Rev. 2013;14(S1):S108–19.

30. CHF International. Accra Poverty Map: A Guide to urban poverty reduction in Accra. 2010.

31. UN-HABITAT. Ghana: Ho City Profile. 2009.

32. Collins LM, Lanza ST. Latent Class and Latent Transition Analysis. Hoboken, New Jersey: Wiley; 2010.

33. Battersby J. The Food Desert as a Concept and Policy Tool in African Cities: An Opportunity

For peer review only

and a Risk. Sustainability. 2019;11(2):458.

34. Gittelsohn J, Trude AC, Poirier L, Ross A, Ruggiero C, Schwendler T, et al. The Impact of a Multi-Level Multi-Component Childhood Obesity Prevention Intervention on Healthy Food Availability, Sales, and Purchasing in a Low-Income Urban Area. Int J Environ Res Public Health. 2017;14(11):1371.

35. WHO. Monitoring Alcohol Marketing in Africa. 2011.

36. Verstraeten R, Leroy JL, Pieniak Z, Ochoa-Aviles A, Holdsworth M, Verbeke W, et al. Individual and Environmental Factors Influencing Adolescents' Dietary Behavior in Low- and Middle-Income Settings. PLoS One. 2016;11(7):e0157744.

37. Miller V, Yusuf S, Chow CK, Dehghan M, Corsi DJ, Lock K, et al. Availability, affordability, and consumption of fruits and vegetables in 18 countries across income levels : findings from the Prospective Urban Rural Epidemiology (PURE) study. Lancet Glob Heal. 2016;4:e695–703.

38. Steyn NP, Mchiza ZJ. Obesity and the nutrition transition in Sub-Saharan Africa. Ann N Y Acad Sci. 2014;1311:88–101.

39. Taylor A, Jacobson M. Carbonating the World: The Marketing and Health Impact of Sugar Drinks in Low- and Middle-income Countries. 2016.

40. Evans CEL. Sugars and health: a review of current evidence and future policy. Proc Nutr Soc. 2017;76(3):400–407.

41. WHO. Global status report on alcohol and health 2018. 2018.

42. Mackay H. Land Use Policy Mapping and characterising the urban agricultural landscape of two intermediate-sized Ghanaian cities. Land use policy. 2018;70:182–97.

43. Doan P, Oduro CY. Patterns of Population Growth in Peri-Urban Accra, Ghana. Int J Urban Reg Res. 2012;36(6):1306–25.

For peer review only

For peer review only - http://bmjopen.bmj.com/site/about/guidelines.xhtml

For peer review only

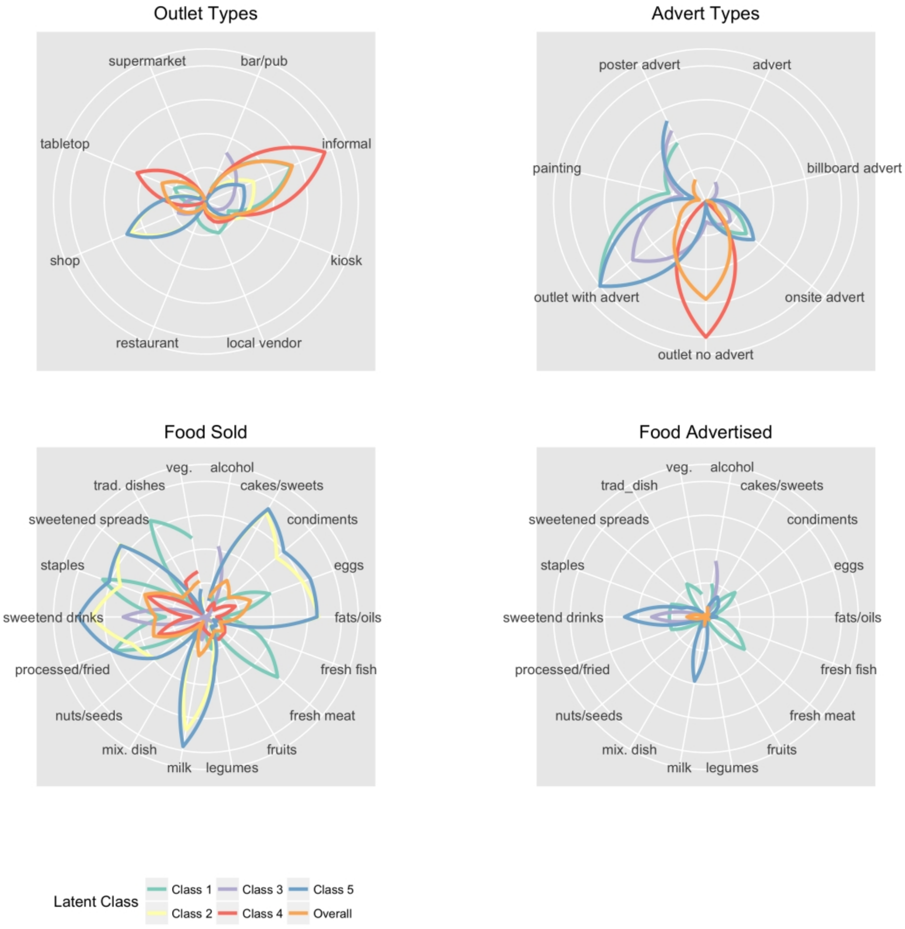

Figure 1

1058x1058mm (72 x 72 DPI)

Supplementary Appendices

For the paper: *Investigating foods and beverages sold and advertised in deprived urban neighbourhoods in Ghana and Kenya: A cross-sectional study.*

Mark A Green PhD[1]*, Rebecca Pradeilles PhD[2], Amos Laar PhD[3], Hibbah Osei-Kwasi PhD[4], Nicolas Bricas MA[5], Nathaniel Coleman MPH[3], Senam Klomegah MPhil[6] , Milka Njeri MSc[7], Akua Tandoh MSc[3], Robert Akparibo PhD[8], Richmond Aryeetey PhD[3], Paula Griffiths PhD[2], Elizabeth Kimani PhD[7], Kobby Mensah PhD[9], Stella Muthuri PhD[10], Francis Zotor PhD[6], Michelle Holdsworth PhD[5,11].

[1] Department of Geography & Planning, University of Liverpool, Liverpool, UK
[2] School of Sport, Exercise and Health Sciences, Loughborough University, Loughborough, UK
[3] Department of Population, Family and Reproductive Health, University of Ghana, Accra, Ghana
[4] Department of Geography, University of Sheffield, Sheffield, UK
[5] UMR Moisa, Centre de Coopération Internationale en Recherche Agronomique pour le Développement (CIRAD) & University of Montpellier, Montpellier, France
[6] Department of Family and Community Health, University of Health and Allied Services, Ho, Ghana
[7] African Population and Health Research Centre, Nairobi, Kenya
[8] School of Health and Related Research (ScHARR), University of Sheffield, Sheffield, UK
[9] Business School, University of Ghana, Accra, Ghana
[10] UK Department of International Development (DFID), Nairobi, Kenya.
[11] French National Research Institute for Sustainable Development (IRD), NUTRIPASS Unit: IRD-Univ Montpellier-SupAgro, Montpellier, France

* Corresponding author: Roxby Building, Department of Geography & Planning, University of Liverpool, Liverpool, L69 7ZT, UK. Telephone: +44 151 794 2854. Email: mark.green@liverpool.ac.uk

For peer review only

Appendix A – Location of study sites

Low income neighbourhoods were randomly selected in each city. In Accra, the selection of a neighbourhood was informed by the Accra Poverty Mapping Exercise (CHF International, 2010; https://www.globalcommunities.org/publications/2010-accra-poverty-map.pdf). Four areas were identified as being poverty endemic. Amongst these, Ga Mashie which is made up of James Town and Ussher Town, was purposively selected. A simple random sampling exercise was then applied and James Town was selected as the neighbourhood of interest. In the city of Ho, the United Nations Human Settlements Programme (UN-HABITAT) urban profiling report informed the selection of the study site. The report highlighted that 36% of the population lived in four poor areas within the city: Bankoe, Hliha, Ahoe and Dome (UNHABITAT 2009; https://uni.unhabitat.org/wp-content/uploads/2014/07/Ghana-Ho-City-Profile.pdf). Amongst these four areas, Dome was then randomly selected. In Nairobi, we used data from the Kenya National Bureau of Statistics (KNBS) to identify the deprivation level of locations (wards), and randomly selected Makadara Constituency. Jericho, Bahati, Maringo, Hamza, Makongeni and Mbotela communities in Makadara were purposively selected as these were areas we could feasibly work in.

Figures A1-A3 visualise the data collection within our study sites. Maps were created using R and the ‘ggmap’ library (1). Base maps were taken from ‘stamen’ maps. Map tiles by Stamen Design, under CC BY 3.0. Data by OpenStreetMap, under ODbL.

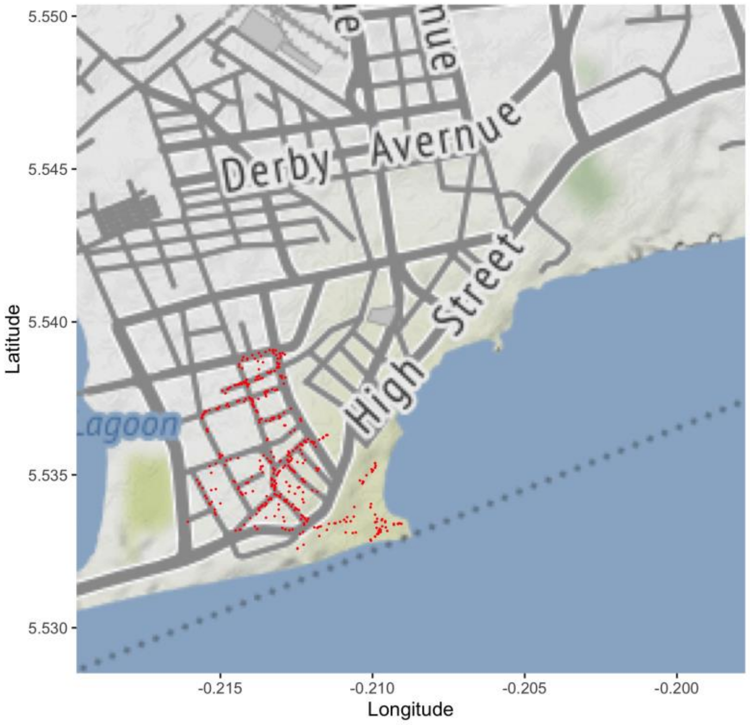

Figure A1: Location of data collection points (red points) within Accra, Ghana.

For peer review only - http://bmjopen.bmj.com/site/about/guidelines.xhtml

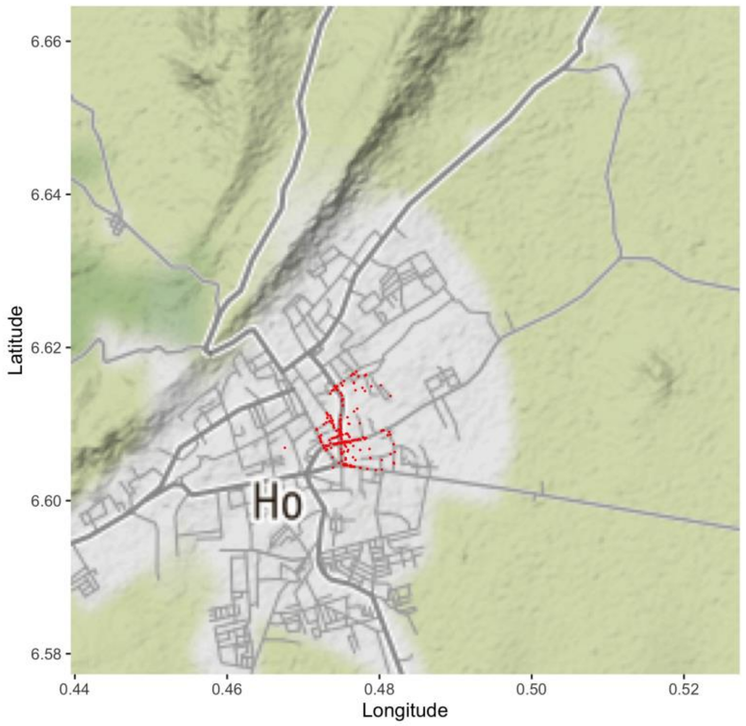

Figure A2: Location of data collection points (red points) within Ho, Ghana.

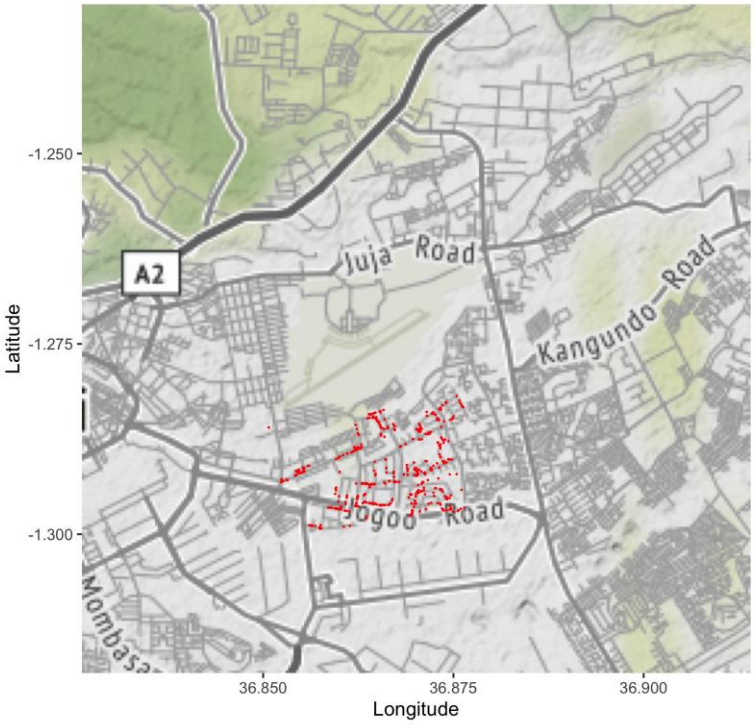

Figure A3: Location of data collection points (red points) within Nairobi, Kenya.

For peer review only - http://bmjopen.bmj.com/site/about/guidelines.xhtml

Appendix B – Description of outlet, advert and food/drink types

Outlet type descriptions

Outlet and advertisement types were defined following a project meeting involving all of the international project partners, researchers and local field workers representing both Ghana and Kenya. The aim was to find consensus over our definitions based on individual expertise and evidence from the wider literature (summarised during discussions), as well as local subject knowledge. Definitions were then validated and refined during the pilot phase of our tool. We opted against using other existing classifications since they were often derived for other settings or countries that were not always relevant to the contexts we were collecting data in. Through designing our own classification, we developed a new system that was relevant to urban Ghana and Kenya, simple and efficient for data collection, and comparable for both countries.

Table B1: Descriptions of each outlet type.

Outlet type	Description
Bar/pub	A formal vendor primarily selling alcohol
Kiosk	A hut or cubicle selling items
Local vendor	Informal street seller operating on the floor (off a mat) or makeshift counter
Restaurant	A formal organisation where individuals sit and consume cooked foods on site
Shop	A small and formal building that sells many items
Supermarket	A large organisation selling a diverse set of goods
Vegetable/fruit/food stand/table top	Informal street seller operating from a single table or stand

We did not record mobile local vendors since they did not have a fixed location, though accept that their non-trivial prevalence means our audit has undercounted the number of outlets. We initially included categories for bakeries (n = 1), chop bars and cold stores (Ghana only, n = 11 and 2 respectively), markets (we record each outlet within the market, not the overall market it itself, n = 1) and an ‘other’ category (n = 36). We did not include these categories in the analyses due to their low prevalence and excluded the data from all analyses.

Table B2: Advertisement descriptions

Outlet type	Description
Onsite	Signs and fittings demonstrating items sold by the outlet
Poster	A small printed paper sign
Painting	The exterior of a building that includes artwork depicting a brand
Billboard	A large outdoor sign

Food type descriptions

Individual items were classified into broader food groups to help improve their interpretation and minimise small number issues. We also grouped foods (based on expert opinions and

For peer review only - http://bmjopen.bmj.com/site/about/guidelines.xhtml

evidence across the literature) into whether we would expect them to increase or decrease in consumption following the nutrition transition to situate our data within broader nutritional trends in African countries.

Table B3: Food category descriptions.

Nutrition transition	Food category	Examples	Healthy**
Expect to increase	Fats/oils	Cooking oils or fats	No
	Sugar sweetened spreads	Jam, hazelnut spread	No
	Fresh meat/poultry	Raw/uncooked beef, pork, chicken	Yes
	Fresh fish/shellfish	Raw/uncooked	Yes
	Milk	Milk	Yes
	Eggs	Eggs	Yes
	Sugar sweetened beverages	Cola, soda	No
	Alcohol	Beer, vodka, wine	No
	Processed/fried foods	Fried plantain, processed meats, pies	No
	Cakes/sweets	Cake, ice cream, chocolate	No
	Modern mixed dishes	Pizza, lasagne,	No
	Condiments*	Ketchup, shito	No
Expect to decrease	Staples	Grains, cereals, roots, tubers	Yes
	Legumes/pulses	Beans, peas, lentils	Yes
	Nuts/seeds	Cashews, sesame seeds	Yes
	Traditional dishes	Jollof rice, banku, waakye	Yes
	Fruits	Mango, watermelon, oranges	Yes
	Vegetables	Peppers, onions	Yes

* Condiments could include products that are commercially processed from multinational companies, as well as those prepared at home or with local small-scale production.
** The classification of foods into “healthy” and “unhealthy” was informed by our related work on dietary intake (as part of the broader project), which used a nutrient profiling classification based on recognised methods (2) of foods consumed in the same cities. We did not classify foods during data collection, however applied the classification after to aid the interpretation of our results.

For peer review only - http://bmjopen.bmj.com/site/about/guidelines.xhtml

Appendix C – Descriptive statistics on items sold by outlet type

Table C1 presents summary statistics on the number of items sold by outlet type. Outlet types that sold a low number of items were bars/pubs, local vendors and vegetable/fruit/food stand/table top. Restaurants, shops, and kiosks sold a greater number of items. The high proportion of vegetable/fruit/food stand/table top in James Town may help to explain the lower number of items sold on average (and lower variety of foods sold) compared to the other locations. Formal outlets also sold a greater diversity of items than compared to informal outlets.

Table C1: Summary statistics of items sold by outlet type.

Outlet type	Mean items sold	Standard Deviation	Per cent selling 1 item	Per cent selling 2 or fewer items	Per cent selling less than 5 items
Bar/pub	2.63	1.87	17.81	75.34	86.30
Kiosk	4.27	2.84	18.95	34.68	61.69
Local vendor	2.98	2.04	26.85	57.41	77.78
Restaurant	6.60	3.39	0.00	10.00	25.00
Shop	6.78	3.55	10.00	19.50	28.00
Supermarket	10.67	3.84	0.00	0.00	11.11
Vegetable/fruit/food stand/table top	3.05	1.94	22.25	47.52	81.59
Informal	3.46	2.38	15.36	31.41	57.34
Formal	5.88	3.76	3.23	9.39	14.29

There were some noticeable differences in the number of advertisements by outlet type (Table C2). You most likely to encounter an advertisement within a supermarket (with the mean count also being three times higher than any other outlet). Bars/pubs also had high proportion of adverts within them, as did restaurants and shops. There was low prevalence in table tops and local vendors. Formal outlets were more likely to contain adverts.

Table C2: Summary statistics of items advertised by outlet type.

Outlet type	Mean items advertised	Standard Deviation	Per cent containing an advert
Bar/pub	1.01	0.84	69.86
Kiosk	0.41	1.06	24.60
Local vendor	0.44	1.30	16.67
Restaurant	1.40	2.19	55.00
Shop	0.95	1.53	48.50
Supermarket	3.44	3.13	88.89
Vegetable/fruit/food stand/table top	0.15	0.77	7.14
Informal	0.28	0.98	10.27
Formal	1.07	1.57	16.34

For peer review only - http://bmjopen.bmj.com/site/about/guidelines.xhtml

Table C3: Percentage of foods sold between formal and informal outlets.

	Formal	Informal
Fats/oils	46.9	16.0
Sugar sweetened spreads	45.2	12.6
Fresh meat/poultry	16.7	16.4
Fresh fish/shellfish	9.8	12.3
Milk	52.5	20.1
Eggs	49.2	30.2
Sugar sweetened beverages	75.7	22.8
Alcohol	40.0	3.5
Processed/fried foods	43.9	40.5
Cakes/sweets	53.8	22.0
Modern mixed dishes	2.3	5.4
Condiments	39.0	16.3
Staples	52.8	47.2
Legumes/pulses	24.3	14.2
Nuts/seeds	27.9	12.2
Traditional dishes	13.1	27.5
Fruits	3.0	16.7
Vegetables	19.0	31.0

Table C4: Percentage of foods advertised between formal and informal outlets.

	Formal	Informal
Fats/oils	3.6	5.5
Sugar sweetened spreads	4.1	2.7
Fresh meat/poultry	6.5	13.6
Fresh fish/shellfish	3.0	8.2
Milk	27.2	20.9
Eggs	3.0	6.4
Sugar sweetened beverages	50.3	42.7
Alcohol	42.6	5.5
Processed/fried foods	7.1	12.7
Cakes/sweets	10.1	6.4
Modern mixed dishes	3.6	4.5
Condiments	5.9	18.2
Staples	14.2	15.5
Legumes/pulses	1.8	1.8
Nuts/seeds	1.8	1.8
Traditional dishes	3.0	12.7
Fruits	1.8	0.9
Vegetables	1.8	7.3

For peer review only

We compared the foods being sold (Table C3) and advertised (Table C4) in formal and informal outlets. The foods sold between formal and informal outlets were somewhat different. Most foods were more common in formal outlets, reflecting that they sold a greater number of items (see Table C1). Noticeably, unhealthy foods displayed were more common in formal outlets (e.g. fats/oils 46.9% vs 16.0%; sugar sweetened spreads 45.2% vs 12.6%), with similar patterns for drinks as well (i.e. sugar sweetened beverages 75.7% vs 22.8%; alcohol 40.0% vs 3.5%). Not all items were more common in formal outlets; vegetables and fruits were more common in informal outlets. Fresh meat, poultry and fish were similar between outlet type. There was little difference in the foods being advertised. Only alcohol displayed a large difference, with it being uncommon in informal outlets than compared to formal outlets where it was more prevalent.

We repeated these summary statistics by specific outlet type as well (see Tables C5 to C7). In summary, they present similar patterns to those described above. Foods sold by local vendors and stands/table tops tended to be dominated by healthier foods and raw ingredients (Table C5). Supermarkets and shops had a greater diversity of foods sold. Pubs and bars mostly sold alcohol and sugar sweetened beverages, with a few selling snacks or fresh meat/poultry. The foods sold by outlets differed to those advertised (Table C6). There was less diversity with lower values reported (bar supermarkets). Many of the foods advertised represented the most common items sold (i.e. the higher values in Table C5). For advert types, drinks were most commonly advertised. These were particularly common in posters, with onsite and painting tending to have a greater diversity of foods advertised (reflecting outlets fronts advertising what they sold).

For peer review only

Table C5: Percentage of foods sold by outlet type.

	Supermarket	Shop	Kiosk	Stand/table top	Local vendor	Restaurant	Bar/pub
Fats/oils	88.9	64.0	30.3	10.2	3.5	25.0	0.0
Sugar sweetened spreads	77.8	62.6	24.3	7.5	3.5	15.0	1.4
Fresh meat/poultry	44.4	6.9	16.7	14.2	22.8	80.0	23.3
Fresh fish/shellfish	22.2	5.9	9.2	12.6	18.4	65.0	4.1
Milk	88.9	72.4	34.3	15.8	2.6	25.0	0.0
Eggs	88.9	63.5	39.0	25.7	25.4	50.0	4.1
Sugar sweetened beverages	88.9	77.8	39.8	15.5	8.8	50.0	75.3
Alcohol	11.1	21.7	4.8	2.4	4.4	25.0	98.6
Processed/fried foods	88.9	51.7	36.7	39.1	53.5	75.0	8.2
Cakes/sweets	0.0	74.9	34.3	19.3	3.5	10.0	1.4
Modern mixed dishes	22.2	1.5	3.2	6.2	7.9	5.0	1.4
Condiments	88.9	53.7	26.7	10.5	12.3	10.0	0.0
Staples	77.8	62.1	51.4	42.1	54.4	95.0	12.3
Legumes/pulses	66.7	29.1	21.5	9.7	13.2	40.0	1.4
Nuts/seeds	77.8	36.9	15.1	12.3	5.3	5.0	2.7
Traditional dishes	22.2	4.4	19.5	31.6	31.6	75.0	19.2
Fruits	22.2	2.5	16.3	17.7	14.0	10.0	0.0
Vegetables	33.3	15.3	33.1	30.0	29.8	55.0	17.8

For peer review only

Table C6: Percentage of foods advertised by outlet type.

	Supermarket	Shop	Kiosk	Stand/table top	Local vendor	Restaurant	Bar/pub
Fats/oils	0.0	6.1	6.6	3.3	5.3	0.0	0.0
Sugar sweetened spreads	25.0	5.1	4.9	0.0	0.0	0.0	0.0
Fresh meat/poultry	0.0	5.1	6.6	13.3	36.8	45.5	2.0
Fresh fish/shellfish	0.0	3.0	3.3	6.7	26.3	18.2	0.0
Milk	50.0	42.4	32.8	6.7	5.3	0.0	0.0
Eggs	12.5	3.0	6.6	10.0	0.0	9.1	0.0
Sugar sweetened beverages	62.5	55.6	52.5	36.7	21.1	45.5	39.2
Alcohol	12.5	15.2	3.3	6.7	10.5	54.5	98.0
Processed/fried foods	37.5	6.1	4.9	20.0	26.3	27.3	0.0
Cakes/sweets	37.5	14.1	9.8	3.3	0.0	0.0	0.0
Modern mixed dishes	12.5	4.0	3.3	6.7	5.3	9.1	0.0
Condiments	12.5	9.1	6.6	30.0	36.8	0.0	0.0
Staples	50.0	18.2	9.8	16.7	31.6	18.2	0.0
Legumes/pulses	12.5	1.0	1.6	3.3	0.0	9.1	0.0
Nuts/seeds	25.0	1.0	3.3	0.0	0.0	0.0	0.0
Traditional dishes	0.0	1.0	6.6	10.0	36.8	18.2	3.9
Fruits	25.0	1.0	0.0	3.3	0.0	0.0	0.0
Vegetables	12.5	1.0	4.9	13.3	5.3	0.0	2.0

For peer review only

Table C7: Percentage of foods advertised by advert type.

	Billboard	Poster	Onsite	Painting
Fats/oils	22.2	3.1	3.6	5.8
Sugar sweetened spreads	11.1	3.6	3.6	1.9
Fresh meat/poultry	11.1	5.7	15.5	13.5
Fresh fish/shellfish	0.0	2.6	10.0	7.7
Milk	22.2	23.8	28.2	21.2
Eggs	11.1	4.7	2.7	1.9
Sugar sweetened beverages	44.4	57.5	49.1	28.8
Alcohol	44.4	34.2	23.6	42.3
Processed/fried foods	0.0	6.2	10.9	13.5
Cakes/sweets	22.2	9.8	10.0	9.6
Modern mixed dishes	11.1	5.7	2.7	3.8
Condiments	11.1	6.2	10.9	17.3
Staples	11.1	15.0	19.1	13.5
Legumes/pulses	0.0	2.6	3.6	0.0
Nuts/seeds	11.1	2.6	0.9	0.0
Traditional dishes	0.0	5.7	10.0	9.6
Fruits	11.1	1.0	0.9	3.8
Vegetables	11.1	3.1	2.7	9.6

For peer review only

Appendix D – Evaluation of the number of latent classes for each model

Latent Class Analysis is an exploratory approach if no theoretical background can determine what the expected latent classes are. Since no previous studies have undertaken a classification of the food environment in Africa, there was no understanding of what latent classes to expect particularly how many classes best describe the data. To select the number of latent classes in our data, we ran multiple models and compared their model fit. We searched for the parsimonious solution. Each additional latent class in our model would likely result in improved model fit, however additional classes introduce added complexity to the model making it harder to interpret.

We ran latent class analyses on our data varying the number of classes between 1 and 10. We did not consider greater than 10 classes since such models would not form much of a data reduction and be difficult to interpret (making any typology less useful). We then compared three model fit statistics (AIC, BIC and G^2) to assess how well they could explain the data. We searched for distinct 'knee points' in graphs of model fit whereby additional classes did not result in significant model improvement (i.e. added complexity in additional classes resulted in minimal gains in model fit). We also compared the model results where it was not clear to aid our interpretation of which model to select.

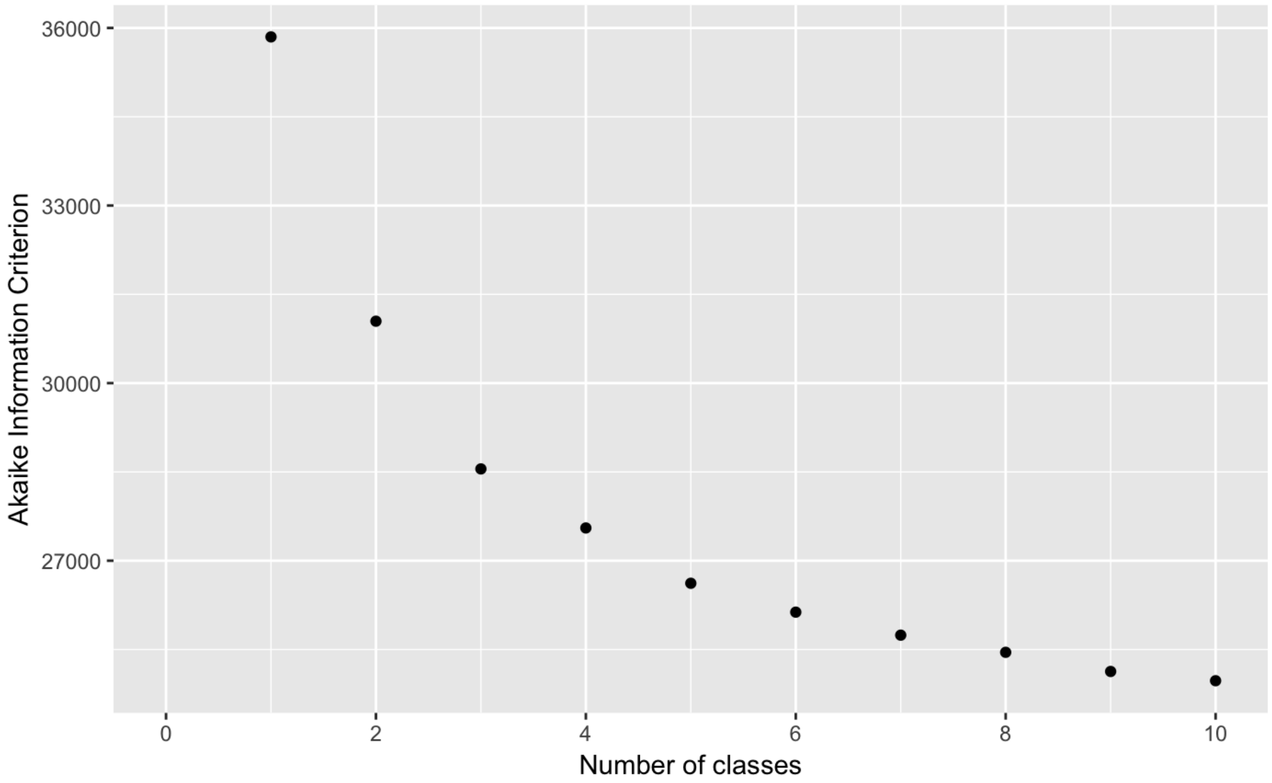

Figure D1: Comparison of Akaike Information Criterion (AIC) for latent class analyses with varying number of classes (1 to 10).

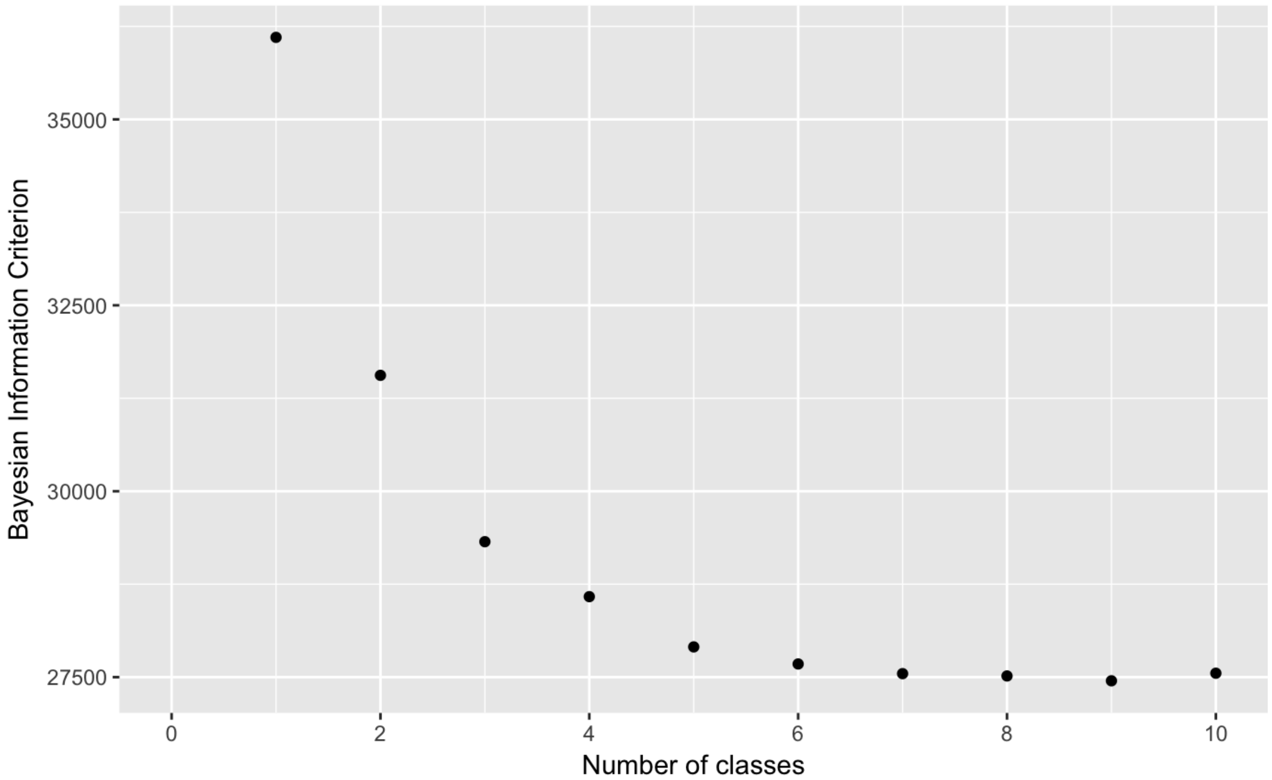

Figure D2: Comparison of Bayesian Information Criterion (BIC) for latent class analyses with varying number of classes (1 to 10).

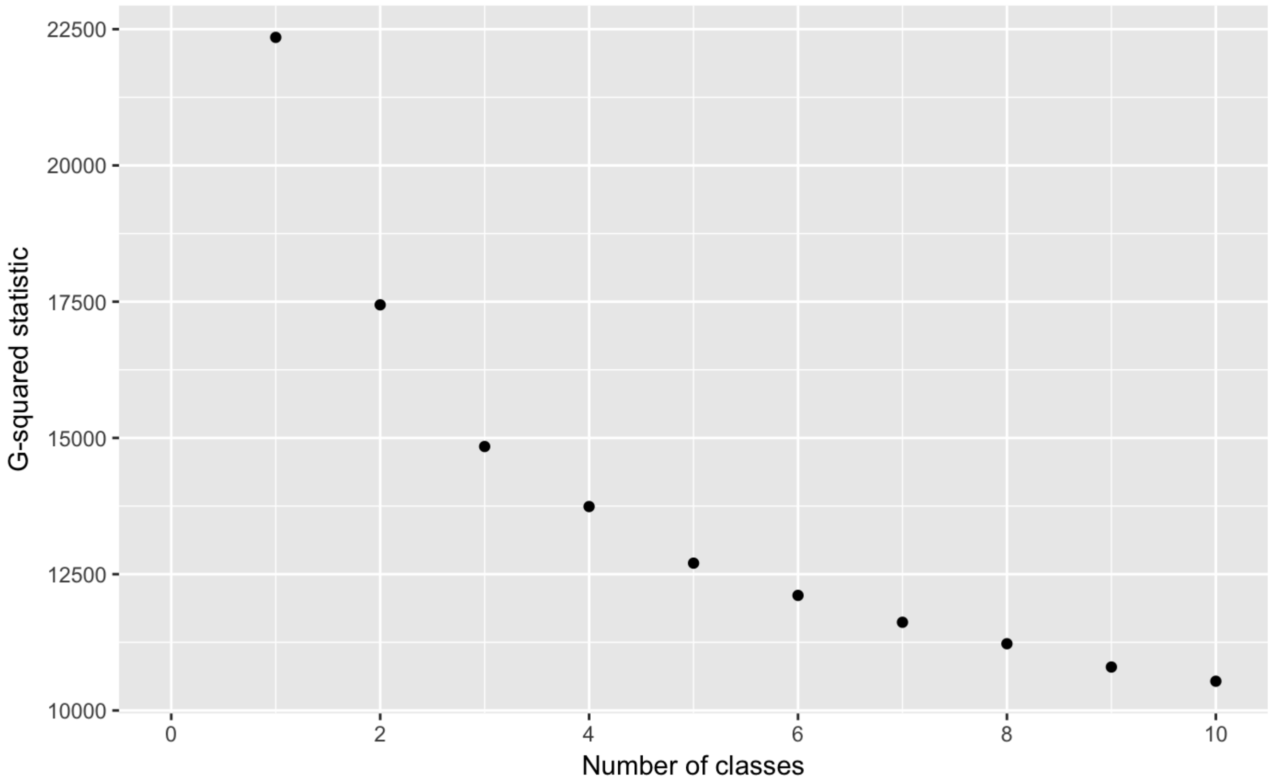

Figure D3: Comparison of G^2 statistic for latent class analyses with varying number of classes (1 to 10).

The AIC and G^2 models produced similar patterns (Figures D1 and D3). For the BIC the pattern was clearer (Figure D2). An increasing number of groups produces better fitting models, but a decreasing rate of improvement. There is no clear knee point though. Following a 5 class solution there is little improvement with subsequent additional class added. Since we are looking for the parsmonious solution, a 5 class solution might work best given that additional classes are not associated with large improvements in model fit. The results for BIC are clearer (Figure D2). We broadly see an improving solution upto 5 classes, whereby model performance is flat onwards. Based on these metrics, a 5 class solution was selected as the final model. Exploring model interpretation of additonal class solutions does not reveal any distinct classes, merely splitting up established classes into less similar classes that differ on small characteristic.

For peer review only

References

1. Kahle D, Wickham H. 2013. ggmap: Spatial Visualization with ggplot2. *The R Journal* 5: 144-161.
2. Drewnowski, A. 2005. Concept of a nutritious food: toward a nutrient density score. *American Journal of Clinical Nutrition* 82: 721–732.

For peer review only

STROBE 2007 (v4) Statement—Checklist of items that should be included in reports of *cross-sectional studies*

Section/Topic	Item #	Recommendation	Reported on page #
**Title and abstract**	1	(*a*) Indicate the study's design with a commonly used term in the title or the abstract	1
		(*b*) Provide in the abstract an informative and balanced summary of what was done and what was found	2
**Introduction**			
Background/rationale	2	Explain the scientific background and rationale for the investigation being reported	4-5
Objectives	3	State specific objectives, including any prespecified hypotheses	5
**Methods**			
Study design	4	Present key elements of study design early in the paper	5-7 and Appendix B
Setting	5	Describe the setting, locations, and relevant dates, including periods of recruitment, exposure, follow-up, and data collection	5-6 and Appendix A-B
Participants	6	(*a*) Give the eligibility criteria, and the sources and methods of selection of participants	N/A
Variables	7	Clearly define all outcomes, exposures, predictors, potential confounders, and effect modifiers. Give diagnostic criteria, if applicable	6 and Appendix B
Data sources/ measurement	8*	For each variable of interest, give sources of data and details of methods of assessment (measurement). Describe comparability of assessment methods if there is more than one group	5-7 and Appendix B
Bias	9	Describe any efforts to address potential sources of bias	None
Study size	10	Explain how the study size was arrived at	5-6
Quantitative variables	11	Explain how quantitative variables were handled in the analyses. If applicable, describe which groupings were chosen and why	5-7
Statistical methods	12	(*a*) Describe all statistical methods, including those used to control for confounding	7 and Appendix C
		(*b*) Describe any methods used to examine subgroups and interactions	N/A
		(*c*) Explain how missing data were addressed	N/A
		(*d*) If applicable, describe analytical methods taking account of sampling strategy	7 and Appendix C
		(*e*) Describe any sensitivity analyses	None
**Results**			

For peer review only

Participants	13*	(a) Report numbers of individuals at each stage of study—eg numbers potentially eligible, examined for eligibility, confirmed eligible, included in the study, completing follow-up, and analysed	7
		(b) Give reasons for non-participation at each stage	N/A
		(c) Consider use of a flow diagram	N/A
Descriptive data	14*	(a) Give characteristics of study participants (eg demographic, clinical, social) and information on exposures and potential confounders	7-10
		(b) Indicate number of participants with missing data for each variable of interest	None
Outcome data	15*	Report numbers of outcome events or summary measures	7-10
Main results	16	(*a*) Give unadjusted estimates and, if applicable, confounder-adjusted estimates and their precision (eg, 95% confidence interval). Make clear which confounders were adjusted for and why they were included	7-12
		(*b*) Report category boundaries when continuous variables were categorized	N/A
		(*c*) If relevant, consider translating estimates of relative risk into absolute risk for a meaningful time period	N/A
Other analyses	17	Report other analyses done—eg analyses of subgroups and interactions, and sensitivity analyses	None
**Discussion**			
Key results	18	Summarise key results with reference to study objectives	12-13
Limitations	19	Discuss limitations of the study, taking into account sources of potential bias or imprecision. Discuss both direction and magnitude of any potential bias	15-16
Interpretation	20	Give a cautious overall interpretation of results considering objectives, limitations, multiplicity of analyses, results from similar studies, and other relevant evidence	12-16
Generalisability	21	Discuss the generalisability (external validity) of the study results	16
**Other information**			
Funding	22	Give the source of funding and the role of the funders for the present study and, if applicable, for the original study on which the present article is based	16-17

*Give information separately for cases and controls in case-control studies and, if applicable, for exposed and unexposed groups in cohort and cross-sectional studies.

Note: An Explanation and Elaboration article discusses each checklist item and gives methodological background and published examples of transparent reporting. The STROBE checklist is best used in conjunction with this article (freely available on the Web sites of PLoS Medicine at http://www.plosmedicine.org/, Annals of Internal Medicine at http://www.annals.org/, and Epidemiology at http://www.epidem.com/). Information on the STROBE Initiative is available at www.strobe-statement.org.

For peer review only

BMJ Open

Investigating foods and beverages sold and advertised in deprived urban neighbourhoods in Ghana and Kenya: A cross-sectional study

Journal:	*BMJ Open*
Manuscript ID	bmjopen-2019-035680.R2
Article Type:	Original research
Date Submitted by the Author:	11-Mar-2020
Complete List of Authors:	Green, Mark; University of Liverpool, Geography & Planning<br>Pradeilles, Rebecca; Loughborough University<br>Laar, Amos; School of Public Health, University of Ghana, Department of Population, Family & Reproductive Health<br>Osei-Kwasi, Hibbah; The University of Sheffield, Geography<br>Bricas, Nicolas; CIRAD<br>Coleman, Nathaniel ; University of Ghana<br>Klomegah, Senam; University of Health and Allied Sciences<br>Wanjohi, Milka ; African Population and Health Research Center<br>Tandoh, Akua; University of Ghana<br>Akparibo, Robert; The University of Sheffield, Health and Related Research<br>Aryeetey, Richmond; University of Ghana<br>Griffiths, Paula; Loughborough University<br>Kimani-Murage, Elizabeth; African Population and HEalth Research Center, Health Systems and Challenges<br>Mensah, Kobby; University of Ghana<br>Muthuri, Stella ; DFID Kenya<br>Zotor, Francis; University of Health and Allied Sciences,<br>Holdsworth, Michelle; French Research Institute for Sustainable Development (IRD)
**Primary Subject Heading**:	Global health
Secondary Subject Heading:	Nutrition and metabolism, Public health, Epidemiology
Keywords:	NUTRITION & DIETETICS, PUBLIC HEALTH, STATISTICS & RESEARCH METHODS

SCHOLARONE™
Manuscripts

I, the Submitting Author has the right to grant and does grant on behalf of all authors of the Work (as defined in the below author licence), an exclusive licence and/or a non-exclusive licence for contributions from authors who are: i) UK Crown employees; ii) where BMJ has agreed a CC-BY licence shall apply, and/or iii) in accordance with the terms applicable for US Federal Government officers or employees acting as part of their official duties; on a worldwide, perpetual, irrevocable, royalty-free basis to BMJ Publishing Group Ltd ("BMJ") its licensees and where the relevant Journal is co-owned by BMJ to the co-owners of the Journal, to publish the Work in this journal and any other BMJ products and to exploit all rights, as set out in our licence.

The Submitting Author accepts and understands that any supply made under these terms is made by BMJ to the Submitting Author unless you are acting as an employee on behalf of your employer or a postgraduate student of an affiliated institution which is paying any applicable article publishing charge ("APC") for Open Access articles. Where the Submitting Author wishes to make the Work available on an Open Access basis (and intends to pay the relevant APC), the terms of reuse of such Open Access shall be governed by a Creative Commons licence – details of these licences and which Creative Commons licence will apply to this Work are set out in our licence referred to above.

Other than as permitted in any relevant BMJ Author's Self Archiving Policies, I confirm this Work has not been accepted for publication elsewhere, is not being considered for publication elsewhere and does not duplicate material already published. I confirm all authors consent to publication of this Work and authorise the granting of this licence.

For peer review only

Investigating foods and beverages sold and advertised in deprived urban neighbourhoods in Ghana and Kenya: A cross-sectional study

Mark A Green PhD[1]*, Rebecca Pradeilles PhD[2], Amos Laar PhD[3], Hibbah Osei-Kwasi PhD[4], Nicolas Bricas MA[5], Nathaniel Coleman MPH[3], Senam Klomegah MPhil[6] , Milka Njeri Wanjohi MSc[7], Akua Tandoh MSc[3], Robert Akparibo PhD[8], Richmond Aryeetey PhD[3], Paula Griffiths PhD[2], Elizabeth Kimani PhD[7], Kobby Mensah PhD[9], Stella Muthuri PhD[10], Francis Zotor PhD[6], Michelle Holdsworth PhD[5,11].

[1] Department of Geography & Planning, University of Liverpool, Liverpool, UK

[2] School of Sport, Exercise and Health Sciences, Loughborough University, Loughborough, UK

[3] Department of Population, Family and Reproductive Health, University of Ghana, Accra, Ghana

[4] Department of Geography, University of Sheffield, Sheffield, UK

[5] UMR Moisa, Centre de Coopération Internationale en Recherche Agronomique pour le Développement (CIRAD) & University of Montpellier, Montpellier, France

[6] Department of Family and Community Health, University of Health and Allied Services, Ho, Ghana

[7] African Population and Health Research Centre, Nairobi, Kenya

[8] School of Health and Related Research (ScHARR), University of Sheffield, Sheffield, UK

[9] Business School, University of Ghana, Accra, Ghana

[10] UK Department of International Development (DFID), Nairobi, Kenya.

[11] French National Research Institute for Sustainable Development (IRD), NUTRIPASS Unit: IRD-Univ Montpellier-SupAgro, Montpellier, France

* Corresponding author: Roxby Building, Department of Geography & Planning, University of Liverpool, Liverpool, L69 7ZT, UK. Telephone: +44 151 794 2854. Email: mark.green@liverpool.ac.uk

For peer review only

Abstract

Objectives: The aim of this study was to characterise the local foods and beverages sold and advertised in three deprived urban African neighbourhoods.

Design: Cross-sectional observational study. We undertook an audit of all food outlets (outlet type and food sold) and food advertisements. Descriptive statistics were used to summarise exposures. Latent class analysis was used to explore the interactions between food advertisements, food outlet types and food type availability.

Setting: Three deprived neighbourhoods in African cities: Jamestown in Accra, Ho Dome in Ho (both Ghana) and Makadara in Nairobi (Kenya).

Main outcome measure**:** Types of foods and beverages sold and/or advertised.

Results: Jamestown (80.5%) and Makadara (70.9%) were dominated by informal vendors. There was a wide diversity of foods, with high availability of healthy (e.g. staples, vegetables) and unhealthy foods (e.g. processed/fried foods, sugar-sweetened beverages). Almost half of all advertisements were for sugar-sweetened beverages (48.3%), with higher exposure to alcohol adverts compared to other items as well (28.5%). We identified five latent classes which demonstrated the clustering of healthier foods in informal outlets, and unhealthy foods in formal outlets.

Conclusion: Our study presents one of the most detailed geospatial exploration of the urban food environment in Africa. The high exposure of sugar-sweetened beverages and alcohol both available and advertised represent changing urban food environments. The concentration of unhealthy foods and beverages in formal outlets and advertisements of unhealthy products may offer important policy opportunities for regulation and action.

Keywords: Food environment; food; adverts; sugar sweetened beverages; alcohol, Africa.

For peer review only - http://bmjopen.bmj.com/site/about/guidelines.xhtml

Strengths and Limitations of this Study

- Detailed geospatial data of three deprived neighbourhoods of African cities.
- Inclusion of food and beverages sold and advertised by outlet which are often not collected.
- Multidimensional analyses consider the interactions between outlet types, food and beverages sold and advertised rather than treating factors independently.
- Limitations include descriptive analyses, lack of representativeness of cities, cross-sectional data, and lack of investigation of which environmental features matter for diet.

Word Count

3637

For peer review only

Introduction

Rising prevalence of overweight and obesity has been seen in most African countries. For instance, the prevalence of obesity in Ghanaian adults (≥20 years) in 2013 was 8.1% and 14% for males and females respectively, and 6.3% and 15.2% respectively among Kenyan adults (1). This emerging issue worries policy makers because of the growing prevalence of nutrition-related non-communicable diseases (NR-NCDs).(2–4) The generalised trends in dietary changes, termed the nutrition transition, include increased consumption of fat, particularly vegetable and edible oils; increased added sugar; increased animal-source foods and decreases in cereals and fibre, specifically in coarse grains, staple cereals and pulses.(5,6) The exact nature of changes in dietary behaviours, and the foods that drive the nutrition transition, vary according to region.(7)

Coinciding with these trends in obesity, many African cities have experienced rapid urban growth.(8,9) The rapid growth and upheaval of urban environments has resulted in major changes to the built environment including the provision of shops and services.(10–13) This has the potential to shape the local food environment that individuals are exposed to, defined here as the physical services, outlets and structures that promote, advertise and sell foods and beverages. It includes outlets that sell fresh, pre-packaged and cooked foods and beverages such as local vendors, small shops, supermarkets, but also advertisements relating to foods and beverages. Local street vendors are key sources for purchasing foods and beverages to cook or eat at home, as well as to eat on site.(14) While there are few multinational organisations (e.g. restaurant chains) established in African cities, they are increasingly becoming interested in the African market where eating out is a sign of affluence.(15) Economic growth has resulted in greater disposable income, which is increasingly being spent on foods and beverages. There has also been growth in supermarkets providing a greater variety of foods and beverages, although such stores are not commonly found within poorer neighbourhoods.(11,12,16,17)

For peer review only

For peer review only - http://bmjopen.bmj.com/site/about/guidelines.xhtml

Our understanding of the urban African food environment is limited. There are few studies from Africa (mainly concentrated in South Africa (18)), although studies are increasingly exploring urban food environments in middle-income countries which have some parallels.(19,20) This is despite extensive investigation of the nature, role and impact of food environments on diet and obesity in high-income countries.(21–24) The lack of ecological and environmental research has been identified as a key barrier towards developing successful interventions by policy makers.(25) This is important since obesity prevalence is higher in urban Africa (including in Ghana and Kenya).(15,26)

Most studies in high-income countries have focused on the location of specific types of outlets.(23) Few studies have measured the types of food and beverages sold by outlets that can allow generalisations about whether they are healthy or not.(19,27) Advertisements are rarely incorporated into analyses despite being ever-present features of most environments. Only focusing on outlet type alone and ignoring these broader characteristics limits our ability to build detailed measures of food environments to truly assess their influences on people.(21,27,28) We extend previous approaches by utilising a multi-dimensional approach to measure the location of food outlets and adverts, as well as what food and beverages are being sold or advertised. Exploring the interactions between these factors is important to characterise and therefore monitor the food environments populations are exposed to.(29)

The aim of our study was to characterise the local foods and beverages sold and advertised in three deprived urban African neighbourhoods.

Methodology

Data

Three residential neighbourhoods were selected: Jamestown in Accra (Accra population ~1.5m), Ho Dome in Ho (Ho population ~100,000) both in Ghana, and Makadara in Nairobi, Kenya (Nairobi

For peer review only

population ~3.5m). Appendix A presents details and maps of the three study sites. They were each identified by randomly selecting one deprived neighbourhood or community in each city that was not a slum based on the following sources: In Accra, we were informed by the findings of the Accra Poverty Mapping exercise (30); In Ho, we used data from the United Nations Human Settlements Programme (31); Finally, in Nairobi we used ward-level deprivation estimates from the Kenya National Bureau of Statistics.

We conducted a full audit of each neighbourhood. Community audit approaches are common in studies of the food environment and allow the construction of detailed and focused datasets.(21) Neighbourhoods were defined through discussion with local community groups, and a 250m buffer was placed around the edge of the community to minimise edge effect issues.

The data collection method was piloted in each setting through surveying smaller areas close to where data collection would take place. The tool was tested on each outlet types (number of occasions for each depending on prevalence in an area) and further amendments were made to the tool. Data collection occurred between September and December 2017. Trained researchers recorded any food outlet or advert observed within each study site. We recorded the outlet type using a classification we developed during a project partners workshop (a full list of outlets including descriptions can be found in Appendix B). We defined outlet types as 'informal' if the structure of their shop (if any) was moveable, not permanent and/or a small scale operation. Items sold within outlets were recorded based on a pre-defined framework of how they would be expected to change during the nutrition transition (detailed in Appendix B). All food advertisements within an outlet were also recorded. We recorded the type of advert (see Appendix B for description) and the item it was advertising (using same categories as the items sold). We also recorded any standalone adverts including the type of advert and the food advertised. GPS coordinates of outlet and advert locations were recorded using Garmin handheld devices.

For peer review only - http://bmjopen.bmj.com/site/about/guidelines.xhtml

Ethical approval for the study was acquired by each institution involved in the data collection process. In Ghana, ethics approval was obtained from the Ghana Health Service Ethics Review Committee (references: GHS-ERC 07/09/16 and GHS-ERC 02/05/17). In Kenya, ethics approval was obtained from the African Medical and Research Foundation (AMREF) (reference: ESRC P365/2017). The University of Sheffield recognised both of these approvals as meeting their ethical standards, as did Loughborough University for the Ghana Health Service. Additional ethical approval was obtained from the University of Liverpool (references: 1434 and 2288) and Loughborough University (reference: R17-P142).

Statistical Analysis

Descriptive statistics were used to explore the characteristics of our samples. Latent class analysis was used to identify groups within our data to characterise the food environment. Latent class analysis is useful for identifying subgroups within categorical data that are otherwise unknown.(32) An exploratory approach was used in the absence of an *a priori* understanding of what groups to expect. We included the outlet type, items being sold, advert type, and items being advertised in the analytical models; although this represents a large number of variables we had no prior justification for dropping variables in this exploratory analysis. The number of latent classes were evaluated through assessing model fit. Analyses were completed using R statistical software (version 3.6.2).(33)

Results

Describing the food environments

A total of 413 food outlets or adverts were identified in Jamestown, 208 in Ho Dome, and 499 in Nairobi. We removed 53 observations that were outlets coded as other, resulting in an analytical sample of 1067. Descriptive statistics of the sample are presented in Tables 1 and 2.

For peer review only - http://bmjopen.bmj.com/site/about/guidelines.xhtml

Each of the neighbourhoods contained different concentrations of outlet types (Table 1). While the majority of outlets in both Jamestown (80.5%) and Makadara (70.9%) were defined as informal vendors, the nature of outlet types varied by neighbourhood. Jamestown was dominated by vegetable/fruit stand/table tops (63.9%), with low availability of all the other types of outlets apart from kiosks (15.1%). Makadara was more evenly spread between kiosks (40.0%), local vendors (13.43%), vegetable/fruit/food stand/table top (17.5%) and shops (17.9%). Ho Dome was different with the majority of outlets defined as formal vendors. There was high availability of shops (43.9%), as well as local vendors (22.8%) and vegetable/fruit/food stand/table top (21.1%).

Table 1: Outlet type and items sold by neighbourhood

Groups	Measure	Jamestown	Ho Dome	Makadara	All
	Outlet type (percentage of all outlets)				
Formal	Bar/pub	8.8	5.0	6.6	7.1
	Restaurant	0.0	2.8	3.3	2.0
	Supermarket	0.5	0.6	1.3	0.9
	Shop	10.1	43.9	17.9	19.6
	All formal outlets	19.4	52.3	29.1	29.6
Informal	Kiosk	15.1	3.9	40.0	24.3
	Local vendor	1.6	22.8	13.4	10.6
	Vegetable/fruit stand/table top	63.9	21.1	17.5	35.6
	All informal* outlets	80.5	47.8	70.9	70.5
	Foods and beverages sold (percentage of all outlets)				
Expected to increase during the nutrition transition	Fats/oils	13.5	43.3	28.7	25.5
	Sugar sweetened spreads	6.5	49.4	25.6	22.6
	Fresh meat/poultry	16.9	22.8	14.4	16.8
	Fresh fish/shellfish	14.6	17.8	7.2	11.8
	Milk	24.9	43.3	29.3	30.1
	Eggs	27.8	53.9	37.0	36.5
	Sugar-sweetened beverages	35.8	50.0	37.4	39.0
	Alcohol	16.4	26.1	8.3	14.5
	Processed/fried foods	37.4	68.3	36.3	42.4
	Cakes/sweets	23.1	50.0	32.2	31.9
	Modern mixed dishes	8.1	4.4	1.8	4.6
	Condiments	15.1	43.3	22.5	23.4
Expected to decrease	Staples	25.7	55.9	37.0	39.8
	Legumes/pulses	8.6	25.6	21.9	17.5
	Nuts/seeds	9.1	38.3	15.5	17.1
	Traditional dishes	29.9	30.0	16.2	23.8

For peer review only - http://bmjopen.bmj.com/site/about/guidelines.xhtml

Fruits	2.9	6.1	24.1	12.9
Vegetables	19.5	21.7	37.9	28.1

** Informal/formal outlets were defined by authors and relate to whether the establishment is a long-term structure that could be regulated or not, or was small scale in investment/organisation*

Foods and beverages sold in outlets demonstrated a good diversity of items. Outlets on average offered a mixture of healthy and unhealthy items (see Appendix C for more detail). There was high availability of sugar-sweetened beverages (39%), as well as processed/fried foods (42.4%). Most items were more common in Ho Dome. This reflected the differences in the prevalence of outlets types, with a higher prevalence of formal vendors that sold a greater number of items. Informal outlets had greater availability of healthier foods and fewer alcohol adverts compared to formal vendors. There was lower prevalence of alcohol sold in Makadara. Fresh meat, poultry and fish were all more common in Jamestown and Ho Dome, with fruits and vegetables more common in Makadara.

Table 2: Food and beverage advertisements observed by neighbourhood, type and items advertised

	Jamestown	Ho Dome	Makadara	All
Whether outlets contained an advert (percentage of all records)				
Outlet with advert(s)	25.3	39.1	22.7	26.6
Outlets with no advert	70.2	58.7	76.9	71.1
Standalone advert	4.5	2.2	0.4	2.3
Advert type (percentage of outlets with adverts or standalone adverts)				
Billboard	0.0	7.9	2.8	3.0
Poster	78.3	57.9	50.9	63.6
On site	24.2	36.8	50.0	36.4
Painting	19.2	23.7	10.4	17.2
Foods and beverages advertised (percentage of outlets with adverts or standalone adverts)				
Fats/oils	0.8	5.3	7.6	4.3
Sugar sweetened spreads	2.5	1.3	6.6	3.6
Fresh meat/poultry	5.8	15.8	8.5	9.3
Fresh fish/shellfish	2.5	11.8	1.9	4.6
Milk	11.7	30.3	30.2	22.9
Eggs	3.3	0.0	7.6	4.0
Sugar-sweetened beverages	57.5	34.2	48.1	48.3
Alcohol	34.2	31.6	19.8	28.5
Processed/fried foods	6.7	9.2	11.3	8.9

For peer review only - http://bmjopen.bmj.com/site/about/guidelines.xhtml

Cakes/sweets	1.7	4.0	18.9	8.3
Modern mixed dishes	4.2	6.6	1.9	4.0
Condiments	8.3	15.8	6.6	9.6
Staples	5.8	23.7	16.0	13.9
Legumes/pulses	0.8	1.3	2.8	1.7
Nuts/seeds	0.0	0.0	4.7	1.7
Traditional dishes	5.8	11.8	3.8	6.6
Fruits	0.0	1.3	2.8	1.3
Vegetables	4.2	2.6	4.7	4.0

A quarter of outlets contained advertisements and with the highest percentage in Ho Dome (Table 2; see Appendix C for more detail). Few standalone adverts were observed across each location. The low proportion of billboards reflects that data collection was conducted in residential neighbourhoods, with fieldwork revealing that billboards were mainly found alongside main roads. Paintings were more commonly observed in Jamestown and Ho Dome than Makadara. Posters were the most common type of advert found (63.6%). 'On Site/Front of Outlet' advertisements were also fairly common especially in Makadara, with large scale paintings less prevalent.

On average, the types of foods and beverages advertised were mainly different from those sold by outlets. The most common item advertised was sugar-sweetened beverages (48.3%) and this was consistent across each neighbourhood (including over half of all occasions where adverts were observed in Jamestown). Milk products were also more commonly advertised than most other items. Alcohol was also widely advertised in Jamestown and Ho Dome, but not in Makadara.

A typology of the food environment

We sought to examine the interactions in our measures and classify the food environment. A five-class model was selected as the most appropriate typology (see Appendix D). To help our interpretation of each model, Figure 1 presents radial plots of the conditional response probabilities that describe class characteristics. Where lines reach outwards they represent higher probabilities and therefore a class being more likely to contain that particular variable. We have provided

For peer review only - http://bmjopen.bmj.com/site/about/guidelines.xhtml

qualitative names for each class to further help their interpretation. Interpretation of the latent classes:

1. *Informal outlets selling raw ingredients*: It was the smallest class ($\gamma = 0.06$). There was an even spread of outlet types observed, although the majority were informal vendors ($\rho = 0.68$). All outlets contained adverts, with higher proportions of posters ($\rho = 0.48$), on site ($\rho = 0.38$) and paintings ($\rho = 0.28$). Foods sold were higher for traditional raw and unprocessed ingredients including staples ($\rho = 0.81$), traditional dishes ($\rho = 0.82$), fresh meat/poultry ($\rho = 0.69$), and eggs ($\rho = 0.50$) (although note the high rate of processed/fried foods as well; $\rho = 0.72$). Advertisements largely followed the foods and beverages sold.
2. *Formal outlets selling unhealthy foods and beverages with no adverts*: The class was fairly small ($\gamma = 0.13$). The majority of outlets were shops ($\rho = 0.60$), with the remaining proportion mostly made up of kiosks ($\rho = 0.30$). There was high availability of unhealthy foods sold particularly items typically added to the cooking process such as fats/oils ($\rho = 0.83$), sugar sweetened spreads ($\rho = 0.83$), and condiments ($\rho = 0.68$). Sugar-sweetened beverages ($\rho = 0.84$) and cakes/sweets ($\rho = 0.89$) were also common. There was good availability of some healthier foods such as milk ($\rho = 0.85$), eggs ($\rho = 0.76$) and staples ($\rho = 0.67$). There was low availability of fresh meat, poultry, fish, fruit or vegetables. There were no advertisements.
3. *Drinking establishments*: A relatively small class ($\gamma = 0.14$). Outlets were mainly formal vendors and the majority contained adverts. The class had the highest prevalence of pubs/bars ($\rho = 0.39$), as well as standalone adverts ($\rho = 0.16$). Adverts were mainly posters ($\rho = 0.58$), although there were above average proportions for the other advert types. The class was characterised by the highest proportion of alcohol and sugar-sweetened beverage sold and advertised. There were few other items sold or advertised.
4. *Small informal sellers*: The largest class ($\gamma = 0.57$) was mostly informal outlets ($\rho = 0.95$) with no adverts. It had the highest prevalence of vegetable/fruit stand/table top ($\rho = 0.55$). There

For peer review only - http://bmjopen.bmj.com/site/about/guidelines.xhtml

was low availability of most items. This reflects sellers having a low diversity of items, selling only a few items.

5. *Formal outlets selling unhealthy foods and beverages with adverts*: The final class was also fairly small (γ = 0.11). The characteristics of the class largely followed class 2, with the noticeable difference being that all outlets contained adverts. Foods and beverages that were advertised matched what was being sold. It had the highest proportion of posters (ρ = 0.66) and on site advertisements (ρ = 0.45).

Figure 1: Conditional response probabilities of the latent class model.

We stratified our analyses by neighbourhood, however the results were similar suggesting that our classes were consistent by context (results not reported).

Discussion

Our study presents one of the most detailed investigations of accessibility and exposure to food and beverages sold and advertised within deprived neighbourhoods of African cities. Our findings have relevance both for understanding the food environments within the Ghanaian and Kenyan context, and could be transferable to other African contexts and beyond. We find diverse food environments across each neighbourhood, but also broad similarities. Other than in Ho Dome, outlets were mainly informal vendors (e.g. kiosks or vegetable/fruit stands/table tops). While there was high availability of many healthy foods, there was considerable availability of unhealthy, so called energy-dense nutrient-poor foods and beverages (e.g. sugar-sweetened beverages, fats/oils, processed/fried foods). Advertisements were most frequent for sodas or sweetened beverages, alcohol and milk, with low advertising for other items (particularly healthy foods and beverages). These findings require attention since the availability and promotion of unhealthy energy-dense nutrient poor foods and beverages within deprived urban African environments may place considerable burdens

For peer review only

on health systems. Poor dietary intake and alcohol abuse are key drivers of NCDs accounting for 73.4% of all deaths globally.(4) With ~80% of NCDs occurring in low and middle income countries and an increasing burden in sub-Saharan Africa,(3) the consequences of unhealthy environments are potentially considerable.

Our data reveal good physical availability of food and beverages across three deprived neighbourhoods. We do not find evidence of food deserts in contrast to social inequalities in high-income countries.(21,24,34) Availability is made possible through the informal sector; local sellers are often mobile allowing them to take opportunities of free space to sell items. The growth of informal outlets reflects the rapid urbanisation and change experienced in cities,(8,9,13) and with respect to the food environment our findings suggest that the growth of informal outlets may not necessarily be bad. The greater availability in unhealthy foods and beverage sold and advertised within formal outlets may offer potential strategies to policy makers. Given that they have physical structures (unlike informal outlets), formal outlets may be amenable to planning regulations (e.g. restricting location, rent subsidies) and interventions to promote healthier foods (e.g. price subsidies, promoting healthier products).(35) The same strategy will apply also to advertisements particularly given their focus on sugar-sweetened beverages and alcohol.(36) Such recommendations contrast with experiences in many African countries whereby the state often regard formal actors as having positive influences on food systems and therefore are less interested in any form of regulation.(11,34)

While we find good physical availability of healthy foods, this represents only one aspect of accessibility. There was good availability of unhealthy foods and beverages that are energy-dense and nutrient poor nutrient as well. Exploring the relationship between availability and consumption of foods and beverages represents an important future angle for research.(20,29,37) Acceptability and affordability also represent important domains of accessibility. Our data would suggest that focusing on improving accessibility to healthy foods and beverages only through physical availability

For peer review only - http://bmjopen.bmj.com/site/about/guidelines.xhtml

may not be effective since such items are commonly available. Improving the acceptability or desirability of healthy foods and beverages can only go so far as well. Affordability is paramount; for example, achieving five servings of fruit and vegetables per person per day would cost 52% of household income in low income countries.(38) Making healthy food affordable, as well as available, will be a key area for policy.(12,34)

The higher exposure to sodas or sugar-sweetened beverages in the items sold and advertised was consistent across each location. Such products are often associated with social status, particularly if the product originates from high-income countries.(39) They also reflect considerable efforts by large multinational corporations to increase sales of sugar-sweetened beverages in African nations through investing in infrastructure including adverts.(14) Investment has been pronounced and its effectiveness, when combined alongside television and sponsorship campaigns to improve the acceptability of their products, has altered the food environments of urban areas.(40) Countering these messages through increased education and making healthier alternatives more desirable could be effective, however public health budgets are dwarfed by the marketing budgets of such companies suggesting it may be difficult. Given the association between sugar consumption and NR-NCDs,(5,41) this area represents an urgent policy area.

Alcohol also displayed higher exposure through items sold and advertised. Africa is an emerging market for alcohol producers and companies are using advertisement campaigns to increase the exposure to alcohol impacting on the built environment.(36,42) Tackling such advertisements represents one potential important area for policy. There was less alcohol available and advertised in Kenya than compared to Ghana. This was despite a similar proportion of bars/pubs in each context. While alcohol consumption is far lower in Africa than Europe, for example, recent estimates suggest it Is slightly higher in Kenya compared to Ghana.(42) Alcohol licensing laws are stricter in Kenya, with establishments recently required to have a formal liquor sale licence to sell any alcohol

For peer review only

(with no sales during the day). It suggests that regulation can be effective in minimising exposure to alcohol, and might lead to opportunities to tackling other unhealthy food items.

A strength of our study is our multi-dimensional approach for measuring the interactions across the food environment. Previous studies tend to focus on measuring single aspects of the food environment and their relationship to health outcomes independently. While focusing on specific factors is useful for isolating dietary components that could be targeted with interventions,(22) they ignore how features interact together and therefore are reductionist in how they measure the food environment.(21,27,28) For example, the latent classes reveal how the foods sold and advertised differ considerably by outlet type. Our approach offers one way for the effective monitoring of food environments, which is important for targeting interventions.(29) The unstructured urban growth that most African cities are experiencing has made it difficult for governments to regulate environments. Without effective monitoring and targeting systems in place, the continued unregulated growth of urban areas may act as a catalyst for rising prevalence of obesity and NR-NCDs.(10)

There are several limitations to our study. Although our approach provides a nuanced way to measure the food environment, it is not all encompassing. While we included a lot of detail in our measures, important aspects of food supply were not collected (e.g. mobile food vendors). We did not consider the role of urban farming of crops and animals.(43) Product placement, shelf space, price and promotional offers are each stimuli influencing food choices within outlets.(27) Data were static representing a single time point, but African food environments evolve through the day, and are vibrant and busy at night. Food hygiene and sanitation surrounding outlets is an important determinant of food borne diseases and diarrhoea. We also focussed on the local neighbourhood environments, but this ignores the wider environments that individuals engage in during their days e.g. school environments.(37) Extending our approach towards developing multi-site and multi-scale measures will help to build more nuanced measures that incorporate a greater range of contexts

For peer review only - http://bmjopen.bmj.com/site/about/guidelines.xhtml

that may influence dietary behaviours. Our study though provides a useful starting point for developing more nuanced and detailed understanding of the food environment.

A further limitation is that while our study sites provide insightful findings of the local food environment, each location is not representative of its city. The rapid growth of many African cities has been unplanned, unstructured and almost random resulting in diverse and heterogeneous communities.(44) Extending study sites to a greater range of neighbourhoods will help to evaluate the interpretation our findings. Our data are cross-sectional and could have been improved through longitudinal observations to explore the consistency of our findings. This is a common issue in studies of the food environment.(22–24) Finally, our study is purely descriptive of food environments and future studies should extend our analyses to explore the extent that exposure to food sold and advertised influence dietary behaviours.

Conclusions

Our study presents one of the most detailed explorations of the food environment in African cities. The physical built environment is a largely ignored aspect of food policy focus in the urban African context, however our findings demonstrate the need to consider the geographical context relating to the foods and beverages sold and advertised within neighbourhoods. Healthy foods commonly available within each neighbourhood suggests that focusing on availability alone may not be effective. Regulating formal marketing and advertisements may be more appropriate, and understanding how to effectively intervene to reduce their exposure will be important for effective policy implementation.

Funding Statement

For peer review only - http://bmjopen.bmj.com/site/about/guidelines.xhtml

This work was supported by two funders. The 'Dietary transitions in Ghana' project was funded by a grant from the Drivers of Food Choice (DFC) Competitive Grants Programme [grant number OPP1110043] which is funded by the Bill & Melinda Gates Foundation and the Department for International Development (DFID), and managed by the University of South Carolina Arnold School of Public Health, USA. DFC supports new research on understanding food choice among the poor in low and middle-income countries, strengthening country-level leadership in nutrition and fostering a global community of food-choice researchers. The TACLED project was funded by a Global Challenges Research Fund (GCRF) Foundation Award led by the MRC [grant number MR/P025153/1], and supported by AHRC, BBSRC, ESRC and NERC, with the aim of improving the health and prosperity of low and middle-income countries. The funders played no role in the design of the study, data collection, data analysis, interpretation of the data, writing of the report or the decision to submit for publication.

Competing Interests Statement

None declared.

Patient and Public Involvement

No involvement in the specific study.

Data Sharing Statement

All analytical code can be viewed at https://github.com/markagreen/Food_environment_ghana_kenya. Data will be archived at the University of Sheffield's Records Management Service (http://www.shef.ac.uk/cics/records). A data

For peer review only

repository (Dataverse Network &/or ORDA) will be used for this purpose. Data will be openly available 12 months after the project completion date (June 2019). Data can be made available securely at an earlier date on reasonable request to the corresponding author.

Author contributions

MAG, AL, RA, PG, EK, KM, FZ, and MH had the idea for the study. All authors were involved in designing the data collection approach and tools. NC, SK, MNW and AT collected the data under the supervision of AL, EK, SM, and FZ. MAG, RP, HOK, NB and MH undertook the statistical analyses, with all authors scrutinising the results. MAG led the writing of the paper, with all authors contributing to revising and approving the paper. The corresponding author (MAG) had full access to all the data in the study and had final responsibility for the decision to submit for publication.

Patient and Public Involvement

No involvement.

References

1. Ng M, Fleming T, Robinson M, Thomson B, Graetz N, Margono C, et al. Global, regional, and national prevalence of overweight and obesity in children and adults during 1980 – 2013: a systematic analysis for the Global Burden of Disease Study 2013. Lancet. 2014;384:766–81.

2. Mbanya JC, Assah FK, Saji J, Atanga EN. Obesity and Type 2 Diabetes in Sub-Sahara Africa.

For peer review only - http://bmjopen.bmj.com/site/about/guidelines.xhtml

Curr Diab Rep. 2014;14:501.

3. Gouda H, Charlson F, Sorsdahl K, Ahmadzada S, Ferrari A, Erskine H, et al. Burden of non-communicable diseases in sub-Saharan Africa, 1990–2017: results from the Global Burden of Disease Study 2017. Lancet Glob Heal. 2019;7(10):E1375–87.

4. GBD 2017 Causes of Death Collaborators. Global, regional, and national age-sex-specific mortality for 282 causes of death in 195 countries and territories, 1980–2017: a systematic analysis for the Global Burden of Disease Study 2017. Lancet. 2018;392(10159):1736–88.

5. Popkin BM, Adair LS, Ng SW. Global nutrition transition and the pandemic of obesity in developing countries. Nutr Rev. 2011;70(1):3–21.

6. Vorster H, Kruger A, Margetts B. The nutrition transition in Africa: Can it be steered into a more positive direction? Nutrients. 2011;3:429–41.

7. Rousham E, Pradeilles R, Akparibo R, Aryeetey R, Bash K, Booth A, et al. Dietary behaviours in the context of nutrition transition: a systematic review and meta-analysis in two African countries. Public Health Nutr.

8. Montgomery MR. The Urban Transformation of the Developing World. Science (80-). 2008;319(5864):761–4.

9. Linard C, Tatem AJ, Gilbert M. Modelling spatial patterns of urban growth in Africa. Appl Geogr. 2013;44:23–32.

10. Ofori-Asenso R, Agyeman AA, Laar A, Boateng D. Overweight and obesity epidemic in Ghana — a systematic review and meta-analysis. BMC Public Health. 2016;16:1239.

11. Battersby J. Food System Transformation in the Absence of Food System Planning: The Case of Supermarket and Shopping Mall Retail Expansion in Cape Town, South Africa. Built Environ. 2017;43(3):417–30.

For peer review only - http://bmjopen.bmj.com/site/about/guidelines.xhtml

12. Berger M, Helvoirt B Van. Ensuring food secure cities – Retail modernization and policy implications in Nairobi, Kenya. Food Policy. 2018;79:12–22.

13. Battersby J, Marshak M, Mngqibisa N. Mapping the informal food economy of Cape Town, South Africa. 2016.

14. Igumbor EU, Sanders D, Puoane TR, Tsolekile L, Schwarz C, Purdy C, et al. '"Big Food,"' the Consumer Food Environment, Health, and the Policy Response in South Africa. PLoS Med. 2012;9(7):e1001253.

15. Neupane S, Prakash KC, Doku DT. Overweight and obesity among women: analysis of demographic and health survey data from 32 Sub-Saharan African Countries. BMC Public Health. 2016;16:30.

16. Aryeetey R, Oltmans S, Owusu F. Food retail assessment and family food purchase behavior in ashongman estates, Ghana. African J Food, Agric Nutr Dev. 2016;16(4):11386–403.

17. Battersby J. Beyond the food desert: finding ways to speak about urban food security in south africa. Geogr Ann Ser B, Hum Geogr. 2012;94(2):141–59.

18. Lachat C, Roberfroid D, Broeck L Van Den, Briel N Van Den, Nago E, Kruger A, et al. A decade of nutrition research in Africa: assessment of the evidence base and academic collaboration. Public Health Nutr. 2014;18(10):1890–7.

19. Duran A, Diez Roux A, Latorre Mdo R, Jaime P. Neighborhood socioeconomic characteristics and differences in the availability of healthy food stores and restaurants in Sao Paulo, Brazil. Health Place. 2013;23:39–47.

20. Leite F, de Carvalho Cremm E, de Abreu D, Oliveira M, Budd N, Martins P. Association of neighbourhood food availability with the consumption of processed and ultra-processed food products by children in a city of Brazil: a multilevel analysis. Public Health Nutr. 2018;21(1):189–200.

For peer review only

21. Caspi CE, Sorensen G, Subramanian S V, Kawachi I. The local food environment and diet: A systematic review. Health Place. 2012;18(5):1172–87.

22. Feng J, Glass TA, Curriero FC, Stewart WF, Schwartz BS. The built environment and obesity: A systematic review of the epidemiologic evidence. Health Place. 2010;16(2):175–90.

23. Fleischhacker SE, Evenson KR, Rodriguez DA, Ammerman AS. A systematic review of fast food access studies. Obes Rev. 2011;12:e460–71.

24. Cobb LK, Appel LJ, Franco M, Jones-Smith JC, Nur A, Anderson CAM. The Relationship of the Local Food Environment with Obesity: A Systematic Review of Methods, Study Quality, and Results. Obesity. 2015;23(7):1331–44.

25. Holdsworth M, Kruger A, Nago E, Lachat C, Mamiro P, Smit K, et al. African stakeholders' views of research options to improve nutritional status in sub-Saharan Africa. Health Policy Plan. 2015;30:863–74.

26. Wu F, Guo Y, Chatterji S, Zheng Y, Naidoo N, Jiang Y, et al. Common risk factors for chronic non-communicable diseases among older adults in China, Ghana, Mexico, India, Russia and South Africa: the study on global AGEing and adult health (SAGE) wave 1. BMC Public Health. 2015;15:88.

27. Kelly B, Flood VM, Yeatman H. Measuring local food environments: An overview of available methods and measures. Health Place. 2011;17(6):1284–93.

28. Rose D, Bodor JN, Hutchinson PL, Swalm CM. The Importance of a Multi-Dimensional Approach for Studying the Links between Food Access and Consumption. J Nutr. 2010;140:1170–4.

29. Mhurchu CN, Vandevijvere S, Waterlander W, Thornton LE, Kelly B, Cameron AJ, et al. Monitoring the availability of healthy and unhealthy foods and non-alcoholic beverages in community and consumer retail food environments globally. Obes Rev. 2013;14(S1):S108–19.

For peer review only

30. CHF International. Accra Poverty Map: A Guide to urban poverty reduction in Accra. 2010.

31. UN-HABITAT. Ghana: Ho City Profile. 2009.

32. Collins LM, Lanza ST. Latent Class and Latent Transition Analysis. Hoboken, New Jersey: Wiley; 2010.

33. R Core Team. R: A language and environment for statistical computing. 2019.

34. Battersby J. The Food Desert as a Concept and Policy Tool in African Cities: An Opportunity and a Risk. Sustainability. 2019;11(2):458.

35. Gittelsohn J, Trude AC, Poirier L, Ross A, Ruggiero C, Schwendler T, et al. The Impact of a Multi-Level Multi-Component Childhood Obesity Prevention Intervention on Healthy Food Availability, Sales, and Purchasing in a Low-Income Urban Area. Int J Environ Res Public Health [Internet]. 2017;14(11):1371. Available from: https://www.mdpi.com/1660-4601/14/11/1371

36. WHO. Monitoring Alcohol Marketing in Africa. 2011.

37. Verstraeten R, Leroy JL, Pieniak Z, Ochoa-Aviles A, Holdsworth M, Verbeke W, et al. Individual and Environmental Factors Influencing Adolescents' Dietary Behavior in Low- and Middle-Income Settings. PLoS One. 2016;11(7):e0157744.

38. Miller V, Yusuf S, Chow CK, Dehghan M, Corsi DJ, Lock K, et al. Availability, affordability, and consumption of fruits and vegetables in 18 countries across income levels : findings from the Prospective Urban Rural Epidemiology (PURE) study. Lancet Glob Heal. 2016;4:e695–703.

39. Steyn NP, Mchiza ZJ. Obesity and the nutrition transition in Sub-Saharan Africa. Ann N Y Acad Sci. 2014;1311:88–101.

40. Taylor A, Jacobson M. Carbonating the World: The Marketing and Health Impact of Sugar Drinks in Low- and Middle-income Countries [Internet]. 2016. Available from:

For peer review only

https://cspinet.org/sites/default/files/attachment/Final Carbonating the World.pdf

41. Evans CEL. Sugars and health: a review of current evidence and future policy. Proc Nutr Soc. 2017;76(3):400–407.

42. WHO. Global status report on alcohol and health 2018. 2018.

43. Mackay H. Land Use Policy Mapping and characterising the urban agricultural landscape of two intermediate-sized Ghanaian cities. Land use policy. 2018;70:182–97.

44. Doan P, Oduro CY. Patterns of Population Growth in Peri-Urban Accra, Ghana. Int J Urban Reg Res. 2012;36(6):1306–25.

For peer review only

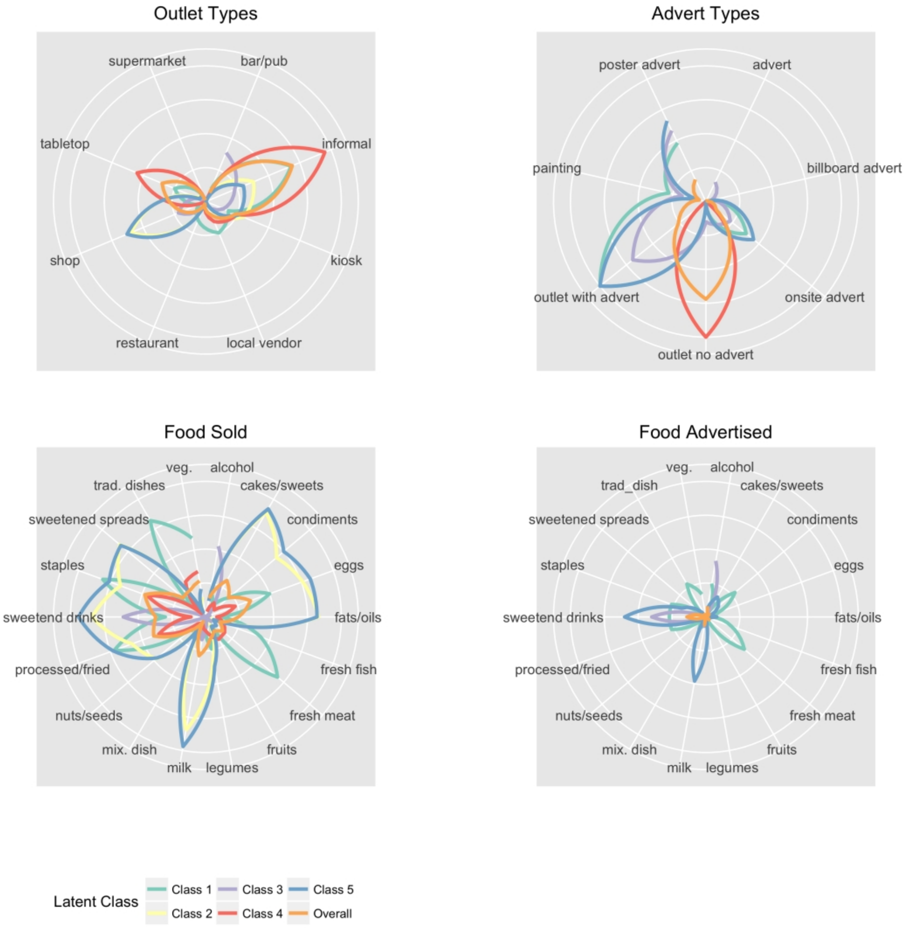

Figure 1

1058x1058mm (72 x 72 DPI)

Supplementary Appendices

For the paper: *Investigating foods and beverages sold and advertised in deprived urban neighbourhoods in Ghana and Kenya: A cross-sectional study.*

Mark A Green PhD[1]*, Rebecca Pradeilles PhD[2], Amos Laar PhD[3], Hibbah Osei-Kwasi PhD[4], Nicolas Bricas MA[5], Nathaniel Coleman MPH[3], Senam Klomegah MPhil[6] , Milka Njeri MSc[7], Akua Tandoh MSc[3], Robert Akparibo PhD[8], Richmond Aryeetey PhD[3], Paula Griffiths PhD[2], Elizabeth Kimani PhD[7], Kobby Mensah PhD[9], Stella Muthuri PhD[10], Francis Zotor PhD[6], Michelle Holdsworth PhD[5,11].

[1] Department of Geography & Planning, University of Liverpool, Liverpool, UK
[2] School of Sport, Exercise and Health Sciences, Loughborough University, Loughborough, UK
[3] Department of Population, Family and Reproductive Health, University of Ghana, Accra, Ghana
[4] Department of Geography, University of Sheffield, Sheffield, UK
[5] UMR Moisa, Centre de Coopération Internationale en Recherche Agronomique pour le Développement (CIRAD) & University of Montpellier, Montpellier, France
[6] Department of Family and Community Health, University of Health and Allied Services, Ho, Ghana
[7] African Population and Health Research Centre, Nairobi, Kenya
[8] School of Health and Related Research (ScHARR), University of Sheffield, Sheffield, UK
[9] Business School, University of Ghana, Accra, Ghana
[10] UK Department of International Development (DFID), Nairobi, Kenya.
[11] French National Research Institute for Sustainable Development (IRD), NUTRIPASS Unit: IRD-Univ Montpellier-SupAgro, Montpellier, France

* Corresponding author: Roxby Building, Department of Geography & Planning, University of Liverpool, Liverpool, L69 7ZT, UK. Telephone: +44 151 794 2854. Email: mark.green@liverpool.ac.uk

For peer review only

Appendix A – Location of study sites

Low income neighbourhoods were randomly selected in each city. In Accra, the selection of a neighbourhood was informed by the Accra Poverty Mapping Exercise (CHF International, 2010; https://www.globalcommunities.org/publications/2010-accra-poverty-map.pdf). Four areas were identified as being poverty endemic. Amongst these, Ga Mashie which is made up of James Town and Ussher Town, was purposively selected. A simple random sampling exercise was then applied and James Town was selected as the neighbourhood of interest. In the city of Ho, the United Nations Human Settlements Programme (UN-HABITAT) urban profiling report informed the selection of the study site. The report highlighted that 36% of the population lived in four poor areas within the city: Bankoe, Hliha, Ahoe and Dome (UNHABITAT 2009; https://uni.unhabitat.org/wp-content/uploads/2014/07/Ghana-Ho-City-Profile.pdf). Amongst these four areas, Dome was then randomly selected. In Nairobi, we used data from the Kenya National Bureau of Statistics (KNBS) to identify the deprivation level of locations (wards), and randomly selected Makadara Constituency. Jericho, Bahati, Maringo, Hamza, Makongeni and Mbotela communities in Makadara were purposively selected as these were areas we could feasibly work in.

Figures A1-A3 visualise the data collected within our study sites. Maps were created using R and the 'ggmap' library (1). Base maps were taken from 'stamen' maps. Map tiles by Stamen Design, under CC BY 3.0. Data by OpenStreetMap, under ODbL.

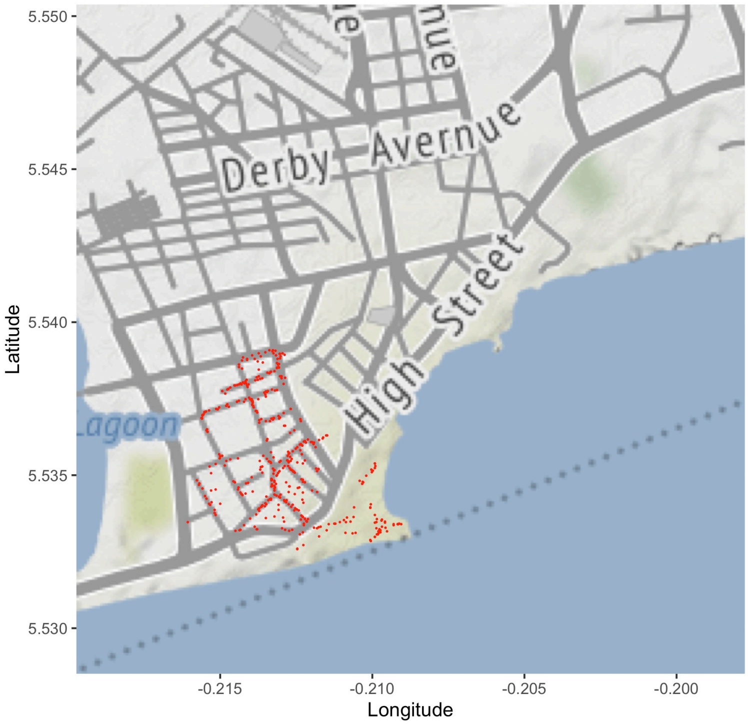

Figure A1: Location of data collection points (red points) within Accra, Ghana.

For peer review only - http://bmjopen.bmj.com/site/about/guidelines.xhtml

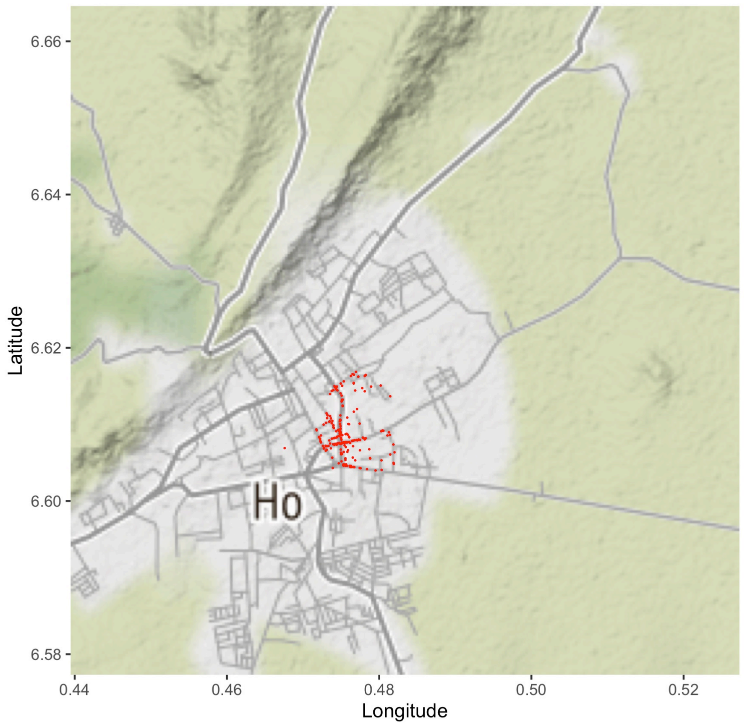

Figure A2: Location of data collection points (red points) within Ho, Ghana.

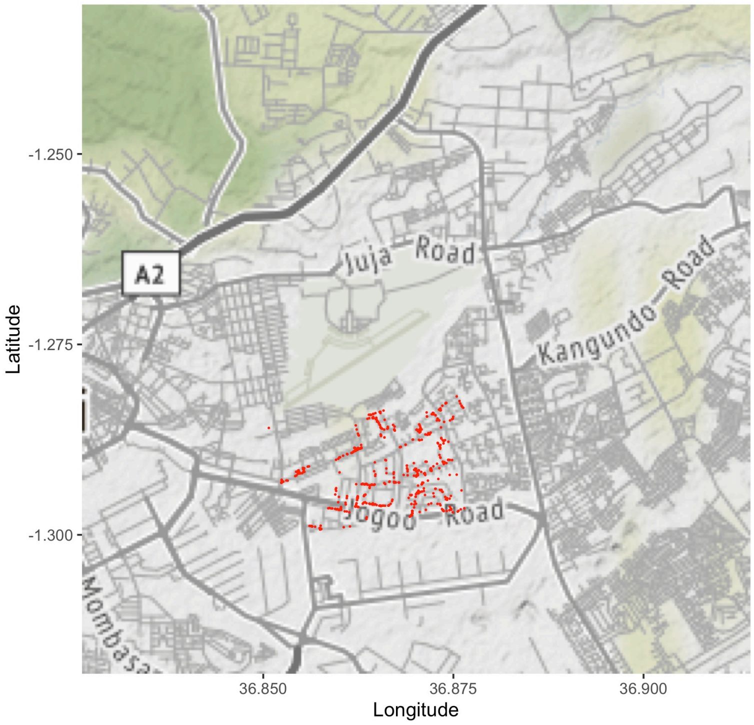

Figure A3: Location of data collection points (red points) within Nairobi, Kenya.

For peer review only - http://bmjopen.bmj.com/site/about/guidelines.xhtml

Appendix B – Description of outlet, advert, food and beverage types

Outlet type descriptions

Outlet and advertisement types were defined following a project meeting involving all of the international project partners, researchers and local field workers representing both Ghana and Kenya. The aim was to find consensus over our definitions based on individual expertise and evidence from the wider literature (summarised during discussions), as well as local subject knowledge. Definitions were then validated and refined during the pilot phase of our tool. We opted against using other existing classifications since they were often derived for other settings or countries that were not always relevant to the contexts we were collecting data in. Through designing our own classification, we developed a new system that was relevant to both urban Ghana and Kenya, as well as simple and efficient for data collection.

Table B1: Descriptions of each outlet type.

Outlet type	Description
Bar/pub	A formal vendor primarily selling alcohol
Kiosk	A hut or cubicle selling items
Local vendor	Informal street seller operating on the floor (off a mat) or makeshift counter
Restaurant	A formal organisation where individuals sit and consume foods and beverages on site
Shop	A small and formal building that sells many items
Supermarket	A large organisation selling a diverse set of goods
Vegetable/fruit/food stand/table top	Informal street seller operating from a single table or stand

We did not record mobile local vendors since they did not have a fixed location, though accept that their non-trivial prevalence means our audit has undercounted the number of outlets. We initially included categories for bakeries (n = 1), chop bars and cold stores (Ghana only, n = 11 and 2 respectively), markets (we record each outlet within the market, not the overall market it itself, n = 1) and an 'other' category (n = 36). We did not include these categories in the analyses due to their low prevalence and excluded the data from all analyses.

Table B2: Advertisement descriptions

Outlet type	Description
Onsite	Signs and fittings demonstrating items sold by the outlet
Poster	A small printed paper sign
Painting	The exterior of a building that includes artwork depicting a brand
Billboard	A large outdoor sign

Food and beverage type descriptions

Individual items were classified into broader groups to help improve their interpretation and minimise small number issues. We also grouped items (based on expert opinions and evidence across the literature) into whether we would expect them to increase or decrease in

For peer review only - http://bmjopen.bmj.com/site/about/guidelines.xhtml

consumption following the nutrition transition to situate our data within broader nutritional trends in African countries.

Table B3: Food and beverage category descriptions.

Nutrition transition	Category	Examples	Healthy**
Expect to increase	Fats/oils	Cooking oils or fats	No
	Sugar sweetened spreads	Jam, hazelnut spread	No
	Fresh meat/poultry	Raw/uncooked beef, pork, chicken	Yes
	Fresh fish/shellfish	Raw/uncooked	Yes
	Milk	Milk	Yes
	Eggs	Eggs	Yes
	Sugar sweetened beverages	Cola, soda	No
	Alcohol	Beer, vodka, wine	No
	Processed/fried foods	Fried plantain, processed meats, pies	No
	Cakes/sweets	Cake, ice cream, chocolate	No
	Modern mixed dishes	Pizza, lasagne,	No
	Condiments*	Ketchup, shito	No
Expect to decrease	Staples	Grains, cereals, roots, tubers	Yes
	Legumes/pulses	Beans, peas, lentils	Yes
	Nuts/seeds	Cashews, sesame seeds	Yes
	Traditional dishes	Jollof rice, banku, waakye	Yes
	Fruits	Mango, watermelon, oranges	Yes
	Vegetables	Peppers, onions	Yes

* Condiments could include products that are commercially processed from multinational companies, as well as those prepared at home or with local small-scale production.
** The classification of foods and beverages into "healthy" and "unhealthy" was informed by our related work on dietary intake (as part of the broader project), which used a nutrient profiling classification based on recognised methods (2) of foods and beverages consumed in the same cities. We did not classify items during data collection, however applied the classification after to aid the interpretation of our results.

For peer review only - http://bmjopen.bmj.com/site/about/guidelines.xhtml

Appendix C – Descriptive statistics on items sold by outlet type

Table C1 presents summary statistics on the number of items sold by outlet type. Outlet types that sold a low number of items were bars/pubs, local vendors and vegetable/fruit/food stand/table top. Restaurants, shops, and kiosks sold a greater number of items. The high proportion of vegetable/fruit/food stand/table top in James Town may help to explain the lower number of items sold on average (and lower variety as well) compared to the other locations. Formal outlets also sold a greater diversity of items than compared to informal outlets.

Table C1: Summary statistics of items sold by outlet type.

Outlet type	Mean items sold	Standard Deviation	Per cent selling 1 item	Per cent selling 2 or fewer items	Per cent selling less than 5 items
Bar/pub	2.63	1.87	17.81	75.34	86.30
Kiosk	4.27	2.84	18.95	34.68	61.69
Local vendor	2.98	2.04	26.85	57.41	77.78
Restaurant	6.60	3.39	0.00	10.00	25.00
Shop	6.78	3.55	10.00	19.50	28.00
Supermarket	10.67	3.84	0.00	0.00	11.11
Vegetable/fruit/food stand/table top	3.05	1.94	22.25	47.52	81.59
Informal	3.46	2.38	15.36	31.41	57.34
Formal	5.88	3.76	3.23	9.39	14.29

There were some noticeable differences in the number of advertisements by outlet type (Table C2). You most likely to encounter an advertisement within a supermarket (with the mean count also being three times higher than any other outlet). Bars/pubs also had high proportion of adverts within them, as did restaurants and shops. There was low prevalence in table tops and local vendors. Formal outlets were more likely to contain adverts.

Table C2: Summary statistics of items advertised by outlet type.

Outlet type	Mean items advertised	Standard Deviation	Per cent containing an advert
Bar/pub	1.01	0.84	69.86
Kiosk	0.41	1.06	24.60
Local vendor	0.44	1.30	16.67
Restaurant	1.40	2.19	55.00
Shop	0.95	1.53	48.50
Supermarket	3.44	3.13	88.89
Vegetable/fruit/food stand/table top	0.15	0.77	7.14
Informal	0.28	0.98	10.27
Formal	1.07	1.57	16.34

For peer review only

Table C3: Percentage of foods and beverages sold between formal and informal outlets.

	Formal	Informal
Fats/oils	46.9	16.0
Sugar sweetened spreads	45.2	12.6
Fresh meat/poultry	16.7	16.4
Fresh fish/shellfish	9.8	12.3
Milk	52.5	20.1
Eggs	49.2	30.2
Sugar sweetened beverages	75.7	22.8
Alcohol	40.0	3.5
Processed/fried foods	43.9	40.5
Cakes/sweets	53.8	22.0
Modern mixed dishes	2.3	5.4
Condiments	39.0	16.3
Staples	52.8	47.2
Legumes/pulses	24.3	14.2
Nuts/seeds	27.9	12.2
Traditional dishes	13.1	27.5
Fruits	3.0	16.7
Vegetables	19.0	31.0

Table C4: Percentage of foods and beverages advertised between formal and informal outlets.

	Formal	Informal
Fats/oils	3.6	5.5
Sugar sweetened spreads	4.1	2.7
Fresh meat/poultry	6.5	13.6
Fresh fish/shellfish	3.0	8.2
Milk	27.2	20.9
Eggs	3.0	6.4
Sugar sweetened beverages	50.3	42.7
Alcohol	42.6	5.5
Processed/fried foods	7.1	12.7
Cakes/sweets	10.1	6.4
Modern mixed dishes	3.6	4.5
Condiments	5.9	18.2
Staples	14.2	15.5
Legumes/pulses	1.8	1.8
Nuts/seeds	1.8	1.8
Traditional dishes	3.0	12.7
Fruits	1.8	0.9
Vegetables	1.8	7.3

For peer review only - http://bmjopen.bmj.com/site/about/guidelines.xhtml

We compared the foods and beverages being sold (Table C3) and advertised (Table C4) in formal and informal outlets. The items sold between formal and informal outlets were somewhat different. Most products were more common in formal outlets, reflecting that they sold a greater number of items (see Table C1). Noticeably, unhealthy foods were more common in formal outlets (e.g. fats/oils 46.9% vs 16.0%; sugar sweetened spreads 45.2% vs 12.6%), with similar patterns for drinks as well (i.e. sugar sweetened beverages 75.7% vs 22.8%; alcohol 40.0% vs 3.5%). Not all items were more common in formal outlets; vegetables and fruits were more common in informal outlets. Fresh meat, poultry and fish were similar between outlet type. There was little difference in the foods and beverages being advertised. Only alcohol displayed a large difference, with it being uncommon in informal outlets than compared to formal outlets where it was more prevalent.

We repeated these summary statistics by specific outlet type as well (see Tables C5 to C7). In summary, they present similar patterns to those described above. Foods and beverages sold by local vendors and stands/table tops tended to be dominated by healthier foods and raw ingredients (Table C5). Supermarkets and shops had a greater diversity of items sold. Pubs and bars mostly sold alcohol and sugar sweetened beverages, with a few selling snacks or fresh meat/poultry. The foods and beverages sold by outlets differed to those advertised (Table C6). There was less diversity with lower values reported (bar supermarkets). Many of the foods and beverages advertised represented the most common items sold (i.e. the higher values in Table C5). For advert types, drinks were most commonly advertised. These were particularly common in posters, with onsite and painting having a greater diversity of foods and beverages advertised (reflecting outlet fronts advertising what they sold).

For peer review only

Table C5: Percentage of foods and beverages sold by outlet type.

	Supermarket	Shop	Kiosk	Stand/table top	Local vendor	Restaurant	Bar/pub
Fats/oils	88.9	64.0	30.3	10.2	3.5	25.0	0.0
Sugar sweetened spreads	77.8	62.6	24.3	7.5	3.5	15.0	1.4
Fresh meat/poultry	44.4	6.9	16.7	14.2	22.8	80.0	23.3
Fresh fish/shellfish	22.2	5.9	9.2	12.6	18.4	65.0	4.1
Milk	88.9	72.4	34.3	15.8	2.6	25.0	0.0
Eggs	88.9	63.5	39.0	25.7	25.4	50.0	4.1
Sugar sweetened beverages	88.9	77.8	39.8	15.5	8.8	50.0	75.3
Alcohol	11.1	21.7	4.8	2.4	4.4	25.0	98.6
Processed/fried foods	88.9	51.7	36.7	39.1	53.5	75.0	8.2
Cakes/sweets	0.0	74.9	34.3	19.3	3.5	10.0	1.4
Modern mixed dishes	22.2	1.5	3.2	6.2	7.9	5.0	1.4
Condiments	88.9	53.7	26.7	10.5	12.3	10.0	0.0
Staples	77.8	62.1	51.4	42.1	54.4	95.0	12.3
Legumes/pulses	66.7	29.1	21.5	9.7	13.2	40.0	1.4
Nuts/seeds	77.8	36.9	15.1	12.3	5.3	5.0	2.7
Traditional dishes	22.2	4.4	19.5	31.6	31.6	75.0	19.2
Fruits	22.2	2.5	16.3	17.7	14.0	10.0	0.0
Vegetables	33.3	15.3	33.1	30.0	29.8	55.0	17.8

For peer review only

Table C6: Percentage of foods and beverages advertised by outlet type.

	Supermarket	Shop	Kiosk	Stand/table top	Local vendor	Restaurant	Bar/pub
Fats/oils	0.0	6.1	6.6	3.3	5.3	0.0	0.0
Sugar sweetened spreads	25.0	5.1	4.9	0.0	0.0	0.0	0.0
Fresh meat/poultry	0.0	5.1	6.6	13.3	36.8	45.5	2.0
Fresh fish/shellfish	0.0	3.0	3.3	6.7	26.3	18.2	0.0
Milk	50.0	42.4	32.8	6.7	5.3	0.0	0.0
Eggs	12.5	3.0	6.6	10.0	0.0	9.1	0.0
Sugar sweetened beverages	62.5	55.6	52.5	36.7	21.1	45.5	39.2
Alcohol	12.5	15.2	3.3	6.7	10.5	54.5	98.0
Processed/fried foods	37.5	6.1	4.9	20.0	26.3	27.3	0.0
Cakes/sweets	37.5	14.1	9.8	3.3	0.0	0.0	0.0
Modern mixed dishes	12.5	4.0	3.3	6.7	5.3	9.1	0.0
Condiments	12.5	9.1	6.6	30.0	36.8	0.0	0.0
Staples	50.0	18.2	9.8	16.7	31.6	18.2	0.0
Legumes/pulses	12.5	1.0	1.6	3.3	0.0	9.1	0.0
Nuts/seeds	25.0	1.0	3.3	0.0	0.0	0.0	0.0
Traditional dishes	0.0	1.0	6.6	10.0	36.8	18.2	3.9
Fruits	25.0	1.0	0.0	3.3	0.0	0.0	0.0
Vegetables	12.5	1.0	4.9	13.3	5.3	0.0	2.0

For peer review only

Table C7: Percentage of foods and beverages advertised by advert type.

	Billboard	Poster	Onsite	Painting
Fats/oils	22.2	3.1	3.6	5.8
Sugar sweetened spreads	11.1	3.6	3.6	1.9
Fresh meat/poultry	11.1	5.7	15.5	13.5
Fresh fish/shellfish	0.0	2.6	10.0	7.7
Milk	22.2	23.8	28.2	21.2
Eggs	11.1	4.7	2.7	1.9
Sugar sweetened beverages	44.4	57.5	49.1	28.8
Alcohol	44.4	34.2	23.6	42.3
Processed/fried foods	0.0	6.2	10.9	13.5
Cakes/sweets	22.2	9.8	10.0	9.6
Modern mixed dishes	11.1	5.7	2.7	3.8
Condiments	11.1	6.2	10.9	17.3
Staples	11.1	15.0	19.1	13.5
Legumes/pulses	0.0	2.6	3.6	0.0
Nuts/seeds	11.1	2.6	0.9	0.0
Traditional dishes	0.0	5.7	10.0	9.6
Fruits	11.1	1.0	0.9	3.8
Vegetables	11.1	3.1	2.7	9.6

For peer review only

Appendix D – Evaluation of the number of latent classes for each model

Latent Class Analysis is an exploratory approach used when no theoretical background can determine what the expected latent classes are. Since no previous studies have undertaken a classification of the food environment in Africa, there was no understanding of what latent classes to expect particularly how many classes best describe the data. To select the number of latent classes in our data, we ran multiple models and compared their model fit. We searched for the parsimonious solution. Each additional latent class in our model would likely result in improved model fit, however additional classes introduce added complexity to the model making it harder to interpret.

We ran latent class analyses on our data varying the number of classes between 1 and 10. We did not consider greater than 10 classes since such models would not form much of a data reduction and be difficult to interpret (making any typology less useful). We then compared three model fit statistics (AIC, BIC and G^2) to assess how well they could explain the data. We searched for distinct 'knee points' in graphs of model fit whereby additional classes did not result in significant model improvement (i.e. added complexity in additional classes resulted in minimal gains in model fit). We also compared the model results where it was not clear to aid our interpretation of which model to select.

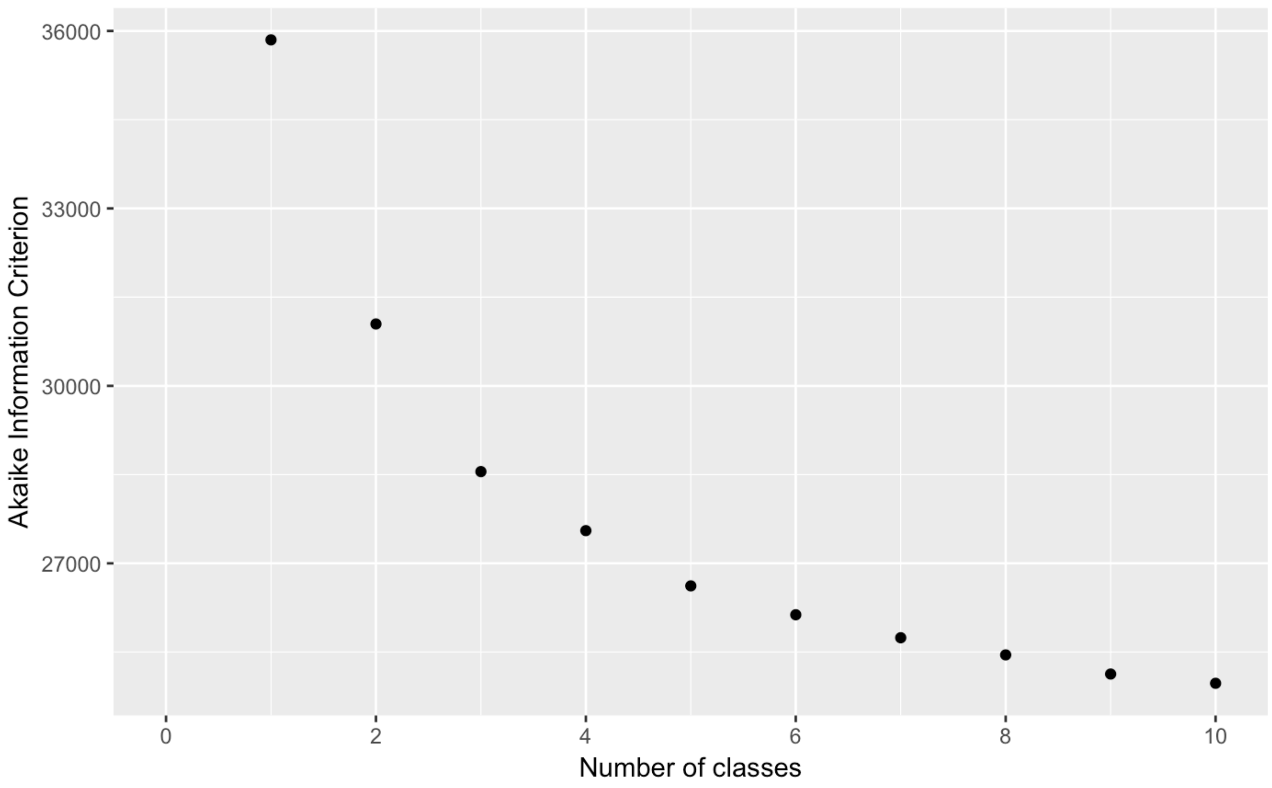

Figure D1: Comparison of Akaike Information Criterion (AIC) for latent class analyses with varying number of classes (1 to 10).

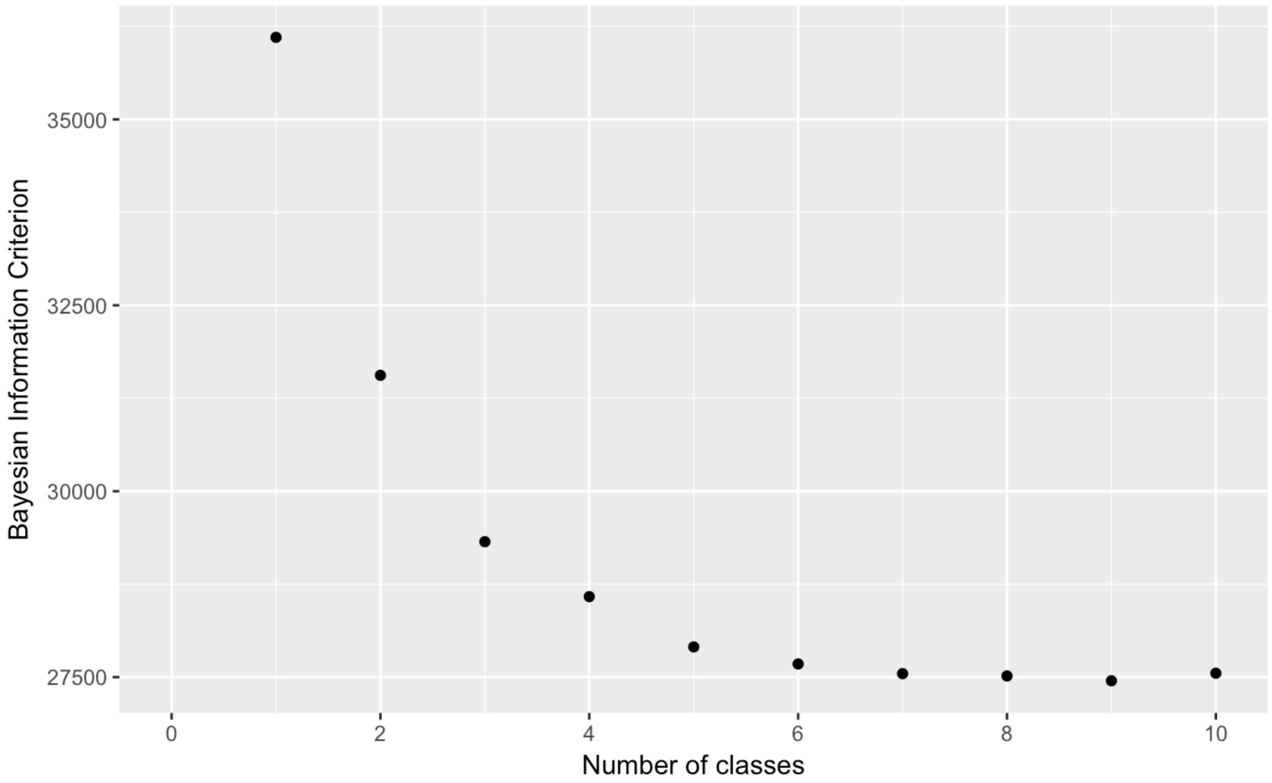

Figure D2: Comparison of Bayesian Information Criterion (BIC) for latent class analyses with varying number of classes (1 to 10).

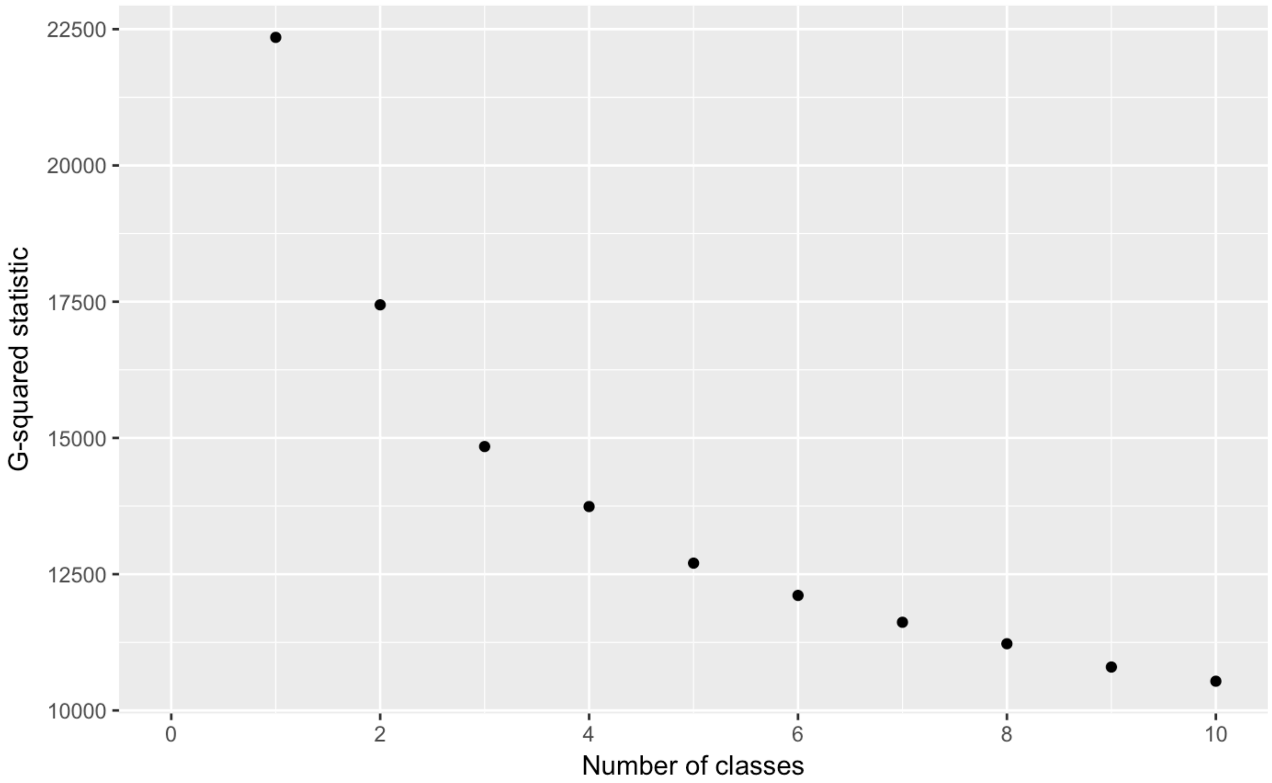

Figure D3: Comparison of G^2 statistic for latent class analyses with varying number of classes (1 to 10).

For peer review only - http://bmjopen.bmj.com/site/about/guidelines.xhtml

The AIC and G^2 models produced similar patterns (Figures D1 and D3). For the BIC the pattern was clearer (Figure D2). An increasing number of groups produces better fitting models, but a decreasing rate of improvement. There is no clear knee point though. Following a 5 class solution there is little improvement with subsequent additional class added. Since we are looking for the parsmonious solution, a 5 class solution might work best given that additional classes are not associated with large improvements in model fit. The results for BIC are clearer (Figure D2). We broadly see an improving solution upto 5 classes, whereby model performance is flat onwards. Based on these metrics, a 5 class solution was selected as the final model. Exploring model interpretation of additonal class solutions does not reveal any distinct classes, merely splitting up established classes into less similar classes that differ on small characteristic.

For peer review only

References

1. Kahle D, Wickham H. 2013. ggmap: Spatial Visualization with ggplot2. *The R Journal* 5: 144-161.
2. Drewnowski, A. 2005. Concept of a nutritious food: toward a nutrient density score. *American Journal of Clinical Nutrition* 82: 721–732.

For peer review only

STROBE 2007 (v4) Statement—Checklist of items that should be included in reports of *cross-sectional studies*

Section/Topic	Item #	Recommendation	Reported on page #
**Title and abstract**	1	(*a*) Indicate the study's design with a commonly used term in the title or the abstract	1
		(*b*) Provide in the abstract an informative and balanced summary of what was done and what was found	2
**Introduction**			
Background/rationale	2	Explain the scientific background and rationale for the investigation being reported	4-5
Objectives	3	State specific objectives, including any prespecified hypotheses	5
**Methods**			
Study design	4	Present key elements of study design early in the paper	5-7 and Appendix B
Setting	5	Describe the setting, locations, and relevant dates, including periods of recruitment, exposure, follow-up, and data collection	5-6 and Appendix A-B
Participants	6	(*a*) Give the eligibility criteria, and the sources and methods of selection of participants	N/A
Variables	7	Clearly define all outcomes, exposures, predictors, potential confounders, and effect modifiers. Give diagnostic criteria, if applicable	6 and Appendix B
Data sources/ measurement	8*	For each variable of interest, give sources of data and details of methods of assessment (measurement). Describe comparability of assessment methods if there is more than one group	5-7 and Appendix B
Bias	9	Describe any efforts to address potential sources of bias	None
Study size	10	Explain how the study size was arrived at	5-6
Quantitative variables	11	Explain how quantitative variables were handled in the analyses. If applicable, describe which groupings were chosen and why	5-7
Statistical methods	12	(*a*) Describe all statistical methods, including those used to control for confounding	7 and Appendix C
		(*b*) Describe any methods used to examine subgroups and interactions	N/A
		(*c*) Explain how missing data were addressed	N/A
		(*d*) If applicable, describe analytical methods taking account of sampling strategy	7 and Appendix C
		(*e*) Describe any sensitivity analyses	None
**Results**			

For peer review only

Participants	13*	(a) Report numbers of individuals at each stage of study—eg numbers potentially eligible, examined for eligibility, confirmed eligible, included in the study, completing follow-up, and analysed	7
		(b) Give reasons for non-participation at each stage	N/A
		(c) Consider use of a flow diagram	N/A
Descriptive data	14*	(a) Give characteristics of study participants (eg demographic, clinical, social) and information on exposures and potential confounders	7-10
		(b) Indicate number of participants with missing data for each variable of interest	None
Outcome data	15*	Report numbers of outcome events or summary measures	7-10
Main results	16	(*a*) Give unadjusted estimates and, if applicable, confounder-adjusted estimates and their precision (eg, 95% confidence interval). Make clear which confounders were adjusted for and why they were included	7-12
		(*b*) Report category boundaries when continuous variables were categorized	N/A
		(*c*) If relevant, consider translating estimates of relative risk into absolute risk for a meaningful time period	N/A
Other analyses	17	Report other analyses done—eg analyses of subgroups and interactions, and sensitivity analyses	None
**Discussion**			
Key results	18	Summarise key results with reference to study objectives	12-13
Limitations	19	Discuss limitations of the study, taking into account sources of potential bias or imprecision. Discuss both direction and magnitude of any potential bias	15-16
Interpretation	20	Give a cautious overall interpretation of results considering objectives, limitations, multiplicity of analyses, results from similar studies, and other relevant evidence	12-16
Generalisability	21	Discuss the generalisability (external validity) of the study results	16
**Other information**			
Funding	22	Give the source of funding and the role of the funders for the present study and, if applicable, for the original study on which the present article is based	16-17

*Give information separately for cases and controls in case-control studies and, if applicable, for exposed and unexposed groups in cohort and cross-sectional studies.

Note: An Explanation and Elaboration article discusses each checklist item and gives methodological background and published examples of transparent reporting. The STROBE checklist is best used in conjunction with this article (freely available on the Web sites of PLoS Medicine at http://www.plosmedicine.org/, Annals of Internal Medicine at http://www.annals.org/, and Epidemiology at http://www.epidem.com/). Information on the STROBE Initiative is available at www.strobe-statement.org.

For peer review only
